# Measuring protected-area effectiveness using vertebrate distributions from leech iDNA

**DOI:** 10.1038/s41467-022-28778-8

**Published:** 2022-03-23

**Authors:** Yinqiu Ji, Christopher C. M. Baker, Viorel D. Popescu, Jiaxin Wang, Chunying Wu, Zhengyang Wang, Yuanheng Li, Lin Wang, Chaolang Hua, Zhongxing Yang, Chunyan Yang, Charles C. Y. Xu, Alex Diana, Qingzhong Wen, Naomi E. Pierce, Douglas W. Yu

**Affiliations:** 1 State Key Laboratory of Genetic Resources and Evolution and Yunnan Key Laboratory of Biodiversity and Ecological Security of Gaoligong Mountain, Kunming Institute of Zoology, 650223 Kunming, Yunnan China; 2 Museum of Comparative Zoology and Department of Organismic & Evolutionary Biology, Harvard University, 26 Oxford Street, Cambridge, MA 02138 USA; 3 Department of Biological Sciences and Sustainability Studies Theme, Ohio University, 107 Irvine Hall, Athens, OH 45701 USA; 4 Center for Environmental Studies (CCMESI), University of Bucharest, 1 N. Balcescu Blvd., Bucharest, Romania; 5 Center for Integrative Conservation, Xishuangbanna Tropical Botanical Garden, Chinese Academy of Sciences, 666303 Mengla, China; 6 Center of Conservation Biology, Core Botanical Gardens, Chinese Academy of Sciences, 666303 Mengla, China; 7 Yunnan Forestry Survey and Planning Institute, 289 Renmin E Rd, 650028 Kunming, Yunnan China; 8 Redpath Museum and Department of Biology, McGill University, 859 Sherbrooke Street West, Montreal, PQ H3A2K6 Canada; 9 School of Mathematics, Statistics and Actuarial Science, University of Kent, Sibson Building, Canterbury, Kent CT27FS UK; 10 Center for Excellence in Animal Evolution and Genetics, Chinese Academy of Sciences, 650201 Kunming, Yunnan China; 11 School of Biological Sciences, University of East Anglia, Norwich Research Park, Norwich, Norfolk NR47TJ UK; 12 Present Address: US Army ERDC Cold Regions Research and Engineering Laboratory, 72 Lyme Road, Hanover, NH 03755 USA

**Keywords:** Conservation biology, Ecological modelling, Molecular ecology, Biodiversity

## Abstract

Protected areas are key to meeting biodiversity conservation goals, but direct measures of effectiveness have proven difficult to obtain. We address this challenge by using environmental DNA from leech-ingested bloodmeals to estimate spatially-resolved vertebrate occupancies across the 677 km^2^ Ailaoshan reserve in Yunnan, China. From 30,468 leeches collected by 163 park rangers across 172 patrol areas, we identify 86 vertebrate species, including amphibians, mammals, birds and squamates. Multi-species occupancy modelling shows that species richness increases with elevation and distance to reserve edge. Most large mammals (e.g. sambar, black bear, serow, tufted deer) follow this pattern; the exceptions are the three domestic mammal species (cows, sheep, goats) and muntjak deer, which are more common at lower elevations. Vertebrate occupancies are a direct measure of conservation outcomes that can help guide protected-area management and improve the contributions that protected areas make towards global biodiversity goals. Here, we show the feasibility of using invertebrate-derived DNA to estimate spatially-resolved vertebrate occupancies across entire protected areas.

## Abstract (web summary)

Invertebrate-derived eDNA (iDNA) is an emerging tool for taxonomic and spatial biodiversity monitoring. Here, the authors use metabarcoding of leech-derived iDNA to estimate vertebrate occupancy over an entire protected area, the Ailaoshan Nature Reserve, China.

## Introduction

In 2010, the signatories to the Convention on Biological Diversity (CBD) agreed to the twenty Aichi Biodiversity Targets for 2011–2020^1^. Aichi Target 11 concerns the safeguarding of biodiversity, and sets the goal of placing 17% of terrestrial and inland water habitats into a system of protected areas (e.g. national parks and other reserves) that is ecologically representative, well-connected, equitably managed, and effective. The world has nearly achieved the areal goal, with 15% of global land area protected under national jurisdiction^2–4^. Contributing to this total, China, a CBD signatory, has placed 15% (1.43 million km^2^) of its own land area into a reserve system^5,6^.

Chinese’s reserve system demonstrates considerable institutional capacity for achieving Aichi Target 11. In western China, for example, the reserves cover most ecoregions, biodiversity priority areas, and natural vegetation types^7^. Landsat imagery shows that the reserves successfully prevent deforestation^8^. But in southern and eastern China, the reserves are not so ecologically representative^9^, many reserves are isolated^7^, there is little information on the impact of reserves on local human populations and, most importantly, we know little about whether the reserves are effective at protecting their biodiversity.

Measuring the effectiveness of protected areas is challenging. Worldwide, it has proven so difficult to assess directly whether protected areas are achieving positive biodiversity outcomes that a recent review deemed their efficacy ‘unknown’^4^. Indirect measures, such as evaluations of staffing and budget adequacy (‘input evaluation’^4^), or evaluations of biodiversity threats like pollution and human pressures (‘threat-reduction evaluation’^4^), are often used as proxies for conservation outcomes, especially where high-throughput technologies such as remote sensing can be employed^2,4,10,11^. However, indirect measures assume that management inputs and/or the reduction of known threats successfully result in positive biodiversity outcomes^4^, are unable to detect whether conservation outcomes differ across taxa, and cannot identify new threats.

In this study, we ask whether we can use environmental DNA (eDNA) to quantify vertebrate biodiversity on a scale large enough for use as a direct measure of protected-area conservation outcomes. We focus on vertebrates (mammals, birds, amphibians and squamates) because one of the most important threats to vertebrate populations in China is overexploitation^12^; this threat is undetectable using remote-sensing methods and is thus especially difficult to measure. Ideally, biodiversity assessments should achieve high spatial and taxonomic resolution. They should allow frequent updates over large areas so that changes in wildlife populations can be detected quickly, allowing causes to be inferred and potentially mitigated. Assessments should be able to be validated rigorously by independent stakeholders and neutral third parties such as courts, and the assessments should be direct – i.e. be based on species detections rather than proxies – both of which are necessary for dispute resolution and for directing and incentivizing effective management. Finally, biodiversity measures should be efficient and simple to understand for decision-makers and the public, contributing to political sustainability and legitimacy^13–15^.

Advances in technologies such as camera traps and bioacoustic recorders allow broad biodiversity monitoring on relatively large scales. Nevertheless, the costs of buying, deploying and monitoring such equipment still imposes some limit on the spatial resolution or extent of monitoring that is feasible. For example, Beaudrot et al.^16^ recently reported on multi-year camera-trap surveys of 511 populations of terrestrial mammals and birds in fifteen tropical-forest protected areas. But while their camera-trap sets covered between 140 and 320 km^2^ in each protected area, this represented only 1–2% of the largest parks in their dataset, reflecting the difficulty and expense of setting up and maintaining a camera-trap network to cover large, difficult-to-access areas, exacerbated by theft and vandalism in some settings^17,18^. Furthermore, both camera traps and acoustic recorders may systematically miss portions of vertebrate biodiversity. For example, amphibians, squamates, and many birds are not readily captured on camera traps; likewise many mammals, amphibians, and squamates may be missed via bioacoustic monitoring.

eDNA has the potential to complement camera traps and bioacoustic recorders^19^, while avoiding some issues of deployment logistics, loss of field equipment, and taxonomic biases. In this study, we focus on iDNA, which is a subset of eDNA^20^, as an emerging sample type for broad taxonomic and spatial biodiversity monitoring. iDNA is vertebrate DNA collected by invertebrate ‘samplers,’ including haematophagous parasites (leeches, mosquitoes, biting flies, ticks) and dung visitors (flies, dung beetles)^21–23^. iDNA methods are rapidly improving, with research focused on documenting the ranges of vertebrate species and their diseases that can be efficiently detected via iDNA^24–29^, comparisons with camera trapping and other survey methods^30–32^, and pipeline development^33,34^.

**Fig. 1:**
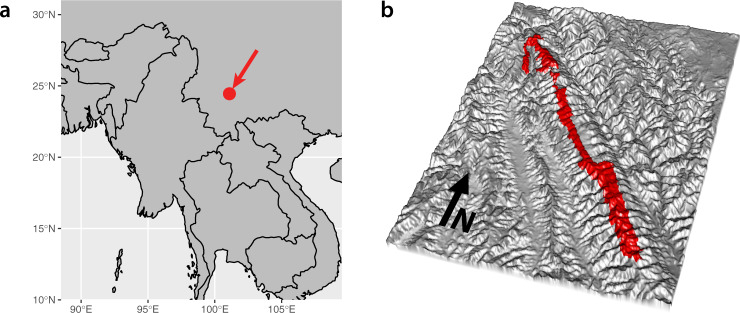
Study site location and layout. **a** The Ailaoshan reserve is located in Yunnan Province, southwest China. Map shows location of reserve with red arrow. **b** The Ailaoshan reserve runs northwest-to-southeast along a ridgeline for ~125 km, but averages just 6 km across along its entire length. Three-dimensional rendering shows reserve with red shading.

We report on the use of iDNA to estimate spatially resolved vertebrate occupancies on the scale of an entire protected area: the 677 km^2^ Ailaoshan reserve in Yunnan province, China (Fig. 1). After the reserve’s establishment in 1981, a 1984–85 survey generated a species list of 86 mammal, 323 bird, 39 (non-avian) reptile and 26 amphibian species/subspecies^35^. Investigators have since carried out one-off targeted surveys^36–38^ and individual-species studies^39–43^. A recent camera-trap study by the Yunnan Forestry Service^44^ detected 10 mammal species and 10 bird species, but was not comprehensive enough to serve as a general vertebrate biodiversity assessment, surveying just 2 of 172 patrol areas in the reserve. Thus, an updated synoptic survey of vertebrate biodiversity remains lacking and, consequently, the current statuses and population trends of vertebrates in the park are largely unknown.

Our study tests the feasibility of employing iDNA surveys within a real protected-area management setting. We had several reasons to explore leech-derived iDNA as a promising broad-scale monitoring technology. First, personnel collecting leeches require little specialised training. The Ailaoshan reserve is divided into 172 patrol areas, each visited monthly by park rangers from neighbouring villages. We contracted these rangers to collect terrestrial, haematophagous leeches during their rainy-season patrols. We were thus able to sample across the reserve in three months at relatively low cost. Second, leech sampling provides an efficient way to correct for imperfect detection, which may include false negatives (i.e. failure to detect species that are present at a site) and false positives (i.e. detecting or appearing to detect a species’ DNA when that species is absent). With leeches, false negatives can arise when, for example, a species was not fed upon by leeches at a site; leeches containing that species’ DNA were not captured from that site; or the species’ DNA was not successfully amplified and associated with the correct taxon. Sources of false positives may include leech movement between sites; sample contamination in the field or lab; and errors in sequencing or bioinformatic processing.

Statistical models can be used to account for imperfect detection. In this project, we analyzed our DNA sequencing results using hierarchical site-occupancy models^45,46^, which distinguish between the detection of a species’ DNA at a site, and the true presence or absence of the species, which is not directly observed. The goal of site-occupancy modelling is to infer where each species is truly present, by separately estimating the probability that a species is present at a site, and the probability that a species is detected if it is present^45,47^. Separating these probabilities relies on a replicated sampling design, with replicates taken in sufficiently close spatial and/or temporal proximity that the underlying distribution of species presences or absences may be treated as fixed. We achieved replicate samples per patrol area in just one patrol by issuing each ranger with multiple, small plastic bags, each containing small tubes with preservative, inducing subsets of leeches to be stored in separate bags^23^, which we processed separately.

A third advantage of leech-derived iDNA is the potential to yield inferences about a broad range of taxa, as leeches are known to feed on small and large mammals, birds, squamates, and amphibians, including arboreal species. This provides a taxonomic breadth that is not typically captured via methods such as camera traps or bioacoustic surveys^27,28,48^. DNA sequences can also potentially distinguish some visually cryptic species^30^ (although iDNA methods can also suffer from a lack of species-level resolution). Finally, leeches can yield PCR-amplifiable DNA for at least four months after their last blood meal^49^, improving the efficiency of leech iDNA by increasing the proportion of collected leeches that can yield information on their previous bloodmeal. On the other hand, leech iDNA persistence could also decrease the spatio-temporal resolution of vertebrate detections, since a long period between leech capture and the previous feed affords more opportunity for leeches or vertebrate hosts to have moved between sampling areas^23^.

In this study, we use metabarcoding^50^ to detect vertebrate species in the blood meals of wild leeches sampled from the Ailaoshan reserve in Yunnan Province, China. We use occupancy modelling to estimate the spatial distributions of the vertebrates throughout the reserve, and identify environmental factors correlated with those distributions. We find that leech-derived iDNA data can identify informative occupancy patterns for a wide range of vertebrates, including species that are less likely to be detected with camera traps and bioacoustic surveys. We conclude that iDNA may be a useful tool for quantifying vertebrate biodiversity, providing a direct measure of protected-area effectiveness and helping achieve conservation outcomes by informing improvements to management strategies.

## Results

### Sampling and metabarcoding

The Ailaoshan reserve runs northwest-to-southeast for around 125 km along a ridgeline (approx. 24.9° N 100.8° E to 24.0° N 101.5° E), averaging just 6 km wide along its length, with elevation between 422 and 3157 m, and annual precipitation between 1000 and 1860 mm depending on altitude^51^ (Fig. 1 and Supplementary Fig. 1a, b). Vegetation is subtropical, evergreen broadleaf forest, and the reserve is flanked by agricultural land on lower-elevation slopes in all directions. There are 261 villages within 5 km of the reserve^52^, with an estimated human population of >20,000.

A total of 30,468 leeches were collected during the rainy season, from July to September 2016, by 163 rangers across 172 ranger patrol areas. These constituted 893 replicate samples after collected leeches were partially pooled in the field or laboratory as described in the Methods section.

We extracted DNA from each replicate sample and PCR-amplified two mitochondrial markers: one from the 16S rRNA gene (*MT-RNR2*), and one from the 12S rRNA gene (*MT-RNR1*). We refer to these two markers as LSU and SSU, respectively, denoting the ribosomal large subunit and small subunit that these genes code for. (We do this to avoid confusion with the widely-used bacterial 16S gene, which is homologous to our 12S marker, rather than our 16S.) After bioinformatic processing of our sequence data, we estimated multispecies site-occupancy models for the LSU and SSU datasets using parameter-expanded data augmentation^46,53^ to accommodate imperfect detection and identify ecological patterns in our datasets.

### Vertebrate species

**Fig. 2:**
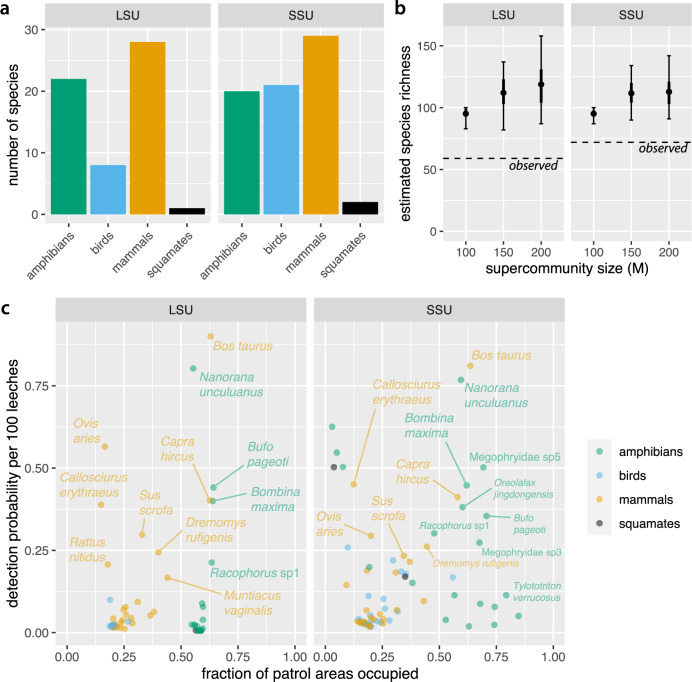
Species richness, occupancy and detection. **a** Distribution of species detected in each dataset by taxonomic group. **b** Estimated species richness over the whole reserve was around 119 species in the LSU dataset and 113 species in the SSU dataset. Plot shows posterior mean (dot), interquartile range (thick line) and 95% Bayesian confidence interval (BCI; thin line with crossbars) from LSU and SSU models based on *n* = 893 replicate samples with different supercommunity size (M) assumptions. Results suggest that the supercommunity size of 200 used for our final models is not materially constraining our estimates. **c** Estimated site occupancy and detection probabilities for each species. Taxa with low occupancy and detection probabilities are unlabelled for clarity; see Supplementary Data 1 for full listing of results.

We identified 86 vertebrate species across the LSU and SSU datasets, in addition to humans. The LSU dataset included 59 species, and the SSU dataset contained 72 species. Although the LSU primers target mammals, both the LSU and SSU primers amplified amphibians, birds, mammals, and squamates, with the general-vertebrate SSU primers amplifying more bird species (Fig. 2a). Forty-five species were common to both datasets, including those identified by their distribution across replicate samples (Supplementary Fig. 2), leaving 14 species unique to LSU and 27 species unique to SSU. We could assign taxonomic names to species level for 58 of our 86 species (45 LSU, 50 SSU). Tables 1 and 2 list the top 20 species in each dataset by estimated occupancy.

**Table 1:** Top species by estimated occupancy in the LSU dataset.

Rank	Scientific name	Common name	IUCN category	Occupancy (95% BCI)
1	*Bufo pageoti*	Tonkin toad (缅甸溪蟾)	NT	0.642 (0.541–0.761)
2	*Bombina maxima*	Yunnan firebelly toad (大蹼铃蟾)	–	0.639 (0.541–0.751)
3	*Rhacophorus* sp1	–	–	0.635 (0.478–0.833)
4	*Bos taurus*	Domestic cattle (黄牛)	–	0.630 (0.545–0.713)
5	*Capra hircus*	Domestic goat (山羊)	–	0.626 (0.493–0.766)
6	*Nanorana yunnanensis*	Yunnan spiny frog (云南棘蛙)	EN	0.597 (0.330–0.842)
7	Megophryidae sp5	–	–	0.596 (0.301–0.890)
8	*Glyphoglossus yunnanensis*	Yunnan small narrow-mouthed frog (云南小狭口蛙)	LC	0.595 (0.234–0.904)
9	*Tylototriton verrucosus*	Himalayan salamander (棕黑疣螈)	LC	0.593 (0.378–0.823)
10	*Nanorana maculosa*	piebald spiny frog (花棘蛙)	VU	0.589 (0.196–0.909)
11	Megophryidae sp4	–	–	0.587 (0.167–0.923)
12	*Leptobrachium ailaonicum*	Ailao moustache toad (哀牢髭蟾)	NT	0.587 (0.182–0.923)
13	*Cynops cyanurus*	cyan newt (蓝尾蝾螈)	LC	0.586 (0.172–0.914)
14	*Kurixalus* sp1	–	–	0.586 (0.182–0.900)
15	Megophryidae sp1	–	–	0.585 (0.182–0.909)
16	*Kurixalus* sp2	–	–	0.584 (0.167–0.909)
17	Megophryidae sp6	–	–	0.580 (0.158–0.923)
18	*Theloderma bicolor*	Chapa bug-eyed frog (双色棱皮树蛙)	EN	0.577 (0.134–0.928)
19	Megophryidae sp2	–	–	0.575 (0.144–0.895)
20	*Amolops mantzorum*	Mouping sucker frog (四川湍蛙)	LC	0.570 (0.196–0.900)

Occupancy represents the posterior mean for the fraction of patrol areas occupied by each species, with 95% Bayesian confidence intervals (BCIs) shown in parentheses. Taxonomic information and IUCN Red List category are based on classification generated by PROTAX. Supplementary Data 1 provides a complete list of species.

*IUCN categories: LC* least concern, *NT* near threatened, *EN* endangered.

**Table 2:** Top species by estimated occupancy in the SSU dataset.

Rank	Scientific name	Common name	IUCN category	Occupancy (95% BCI)
1	Megophryidae sp6	–	–	0.847 (0.541–1.000)
2	*Tylototriton verrucosus*	Himalayan salamander (棕黑疣螈)	LC	0.793 (0.545–1.000)
3	*Leptobrachium ailaonicum*	Ailao moustache toad (哀牢髭蟾)	NT	0.743 (0.383–1.000)
4	*Cynops cyanurus*	cyan newt (蓝尾蝾螈)	LC	0.742 (0.167–1.000)
5	*Bufo pageoti*	Tonkin toad (缅甸溪蟾)	NT	0.707 (0.574–0.852)
6	Megophryidae sp5	–	–	0.693 (0.550–0.847)
7	*Rana chaochiaoensis*	Chaochiao brown frog (昭觉林蛙)	LC	0.679 (0.325–0.995)
8	Megophryidae sp3	–	–	0.676 (0.531–0.833)
9	*Bos taurus*	domestic cattle (黄牛)	–	0.636 (0.550–0.718)
10	*Glyphoglossus yunnanensis*	Yunnan small narrow-mouthed frog (云南小狭口蛙)	LC	0.630 (0.057–1.000)
11	*Bombina maxima*	Yunnan firebelly toad (大蹼铃蟾)	–	0.620 (0.512–0.737)
12	*Oreolalax jingdongensis*	Jingdong toothed toad (景东齿蟾)	VU	0.602 (0.483–0.727)
13	*Nanorana unculuanus*	Yunnan Asian frog (棘肛蛙)	VU	0.595 (0.498–0.694)
14	*Capra hircus*	domestic goat (山羊)	–	0.580 (0.455–0.718)
15	*Nanorana yunnanensis*	Yunnan spiny frog (云南棘蛙)	EN	0.567 (0.249–0.995)
16	Leiothrichidae sp1	–	–	0.559 (0.354–0.823)
17	Anura sp1	–	–	0.528 (0.067–1.000)
18	*Rhacophorus* sp1	–	–	0.478 (0.325–0.660)
19	*Dremomys rufigenis*	red-cheeked squirrel (红颊长吻松鼠)	LC	0.445 (0.306–0.622)
20	*Muntiacus vaginalis*	northern red muntjac (褜麂)	LC	0.432 (0.239–0.766)

Occupancy represents the posterior mean for the fraction of patrol areas occupied by each species, with 95% Bayesian confidence intervals (BCIs) shown in parentheses. Taxonomic information and IUCN Red List category are based on classification generated by PROTAX. Supplementary Data 1 provides a complete list of species.

*IUCN categories: LC* least concern, *NT* near threatened, *EN* endangered.

With the supercommunity size of *M* = 200 that we used for our final occupancy models, estimated total species richness in Ailaoshan was 119 species in the LSU dataset and 113 species in the SSU dataset (Fig. 2b). Setting *M* = 150 produced similar results, while *M* = 100 clearly constrained the species richness estimates.

Domesticated species featured heavily in our data (Supplementary Data 1), consistent with observed grazing of these species in the reserve (DWY, pers. obs.). Domestic cattle (*Bos taurus*) were the most frequently detected taxon in both datasets, being detected in almost half of all patrol areas; domestic goats (*Capra hircus*) were also common, being detected in just under a third of patrol areas, and domestic sheep (*Ovis aries*) were detected in ca. 6% of patrol areas. The *O. aries* detections were concentrated in the reserve’s southeastern section (Xinping county), located near to Shiping town and the main breeding area of the dark-haired Shiping Qin sheep breed.

Several wild taxa detected in our survey are listed as Threatened or Near Threatened by the IUCN (Table 3). Among mammals, four species have IUCN Vulnerable status: Asiatic black bear (*Ursus thibetanus*), mainland serow (*Capricornis milneedwardsii*), sambar (*Rusa unicolor*), and stump-tailed macaque (*Macaca arctoides*). Among amphibians, the Yunnan spiny frog (*Nanorana yunnanensis*) and Chapa bug-eyed frog (*Theloderma bicolor*) are listed as Endangered, while the piebald spiny frog (*Nanorana maculosa*), Yunnan Asian frog (*Nanorana unculuanus*) and Jingdong toothed toad (*Oreolalax jingdongensis*) have Vulnerable status. Some of these taxa, especially the amphibians, were widespread in Ailaoshan (Table 3 and Supplementary Data 1), highlighting the value of this reserve for protecting these species.

**Table 3:** Threatened and near-threatened species.

Group	Scientific name	Common name	IUCN category	LSU occupancy	SSU occupancy
Amphibians	*Bufo pageoti*	Tonkin toad (缅甸溪蟾)	NT	0.642 (0.541–0.761)	0.707 (0.574–0.852)
Amphibians	*Leptobrachium ailaonicum*	Ailao moustache toad (哀牢髭蟾)	NT	0.587 (0.182–0.923)	0.743 (0.383–1.000)
Amphibians	*Nanorana maculosa*	piebald spiny frog (花棘蛙)	VU	0.589 (0.196–0.909)	–
Amphibians	*Nanorana unculuanus*	Yunnan Asian frog (棘肛蛙)	VU	0.553 (0.450–0.656)	0.595 (0.498–0.694)
Amphibians	*Nanorana yunnanensis*	Yunnan spiny frog (云南棘蛙)	EN	0.597 (0.330–0.842)	0.567 (0.249–0.995)
Amphibians	*Oreolalax jingdongensis*	Jingdong toothed toad (景东齿蟾)	VU	–	0.602 (0.483–0.727)
Amphibians	*Theloderma bicolor*	Chapa bug-eyed frog (双色棱皮树蛙)	EN	0.577 (0.134–0.928)	–
Birds	*Cyanoptila cumatilis*	Zappey’s flycatcher (白腹暗蓝鹟)	NT	0.204 (0.014–0.584)	0.244 (0.038–0.794)
Birds	*Syrmaticus humiae*	Mrs Hume’s pheasant (黑颈长尾雉)	NT	–	0.197 (0.024–0.641)
Mammals	*Capricornis milneedwardsii*	mainland serow (中华鬣羚)	VU	0.199 (0.019–0.603)	0.191 (0.019–0.651)
Mammals	*Catopuma temminckii*	Asiatic golden cat (金猫)	NT	–	0.151 (0.010–0.536)
Mammals	*Elaphodus cephalophus*	tufted deer (毛冠鹿)	NT	0.203 (0.029–0.536)	–
Mammals	*Macaca arctoides*	stump-tailed macaque (短尾猴)	VU	0.259 (0.043–0.622)	–
Mammals	*Rusa unicolor*	sambar (水鹿)	VU	0.203 (0.014–0.593)	–
Mammals	*Ursus thibetanus*	Asiatic black bear (亚洲黑熊)	VU	0.287 (0.038–0.718)	0.182 (0.014–0.660)

Detected species categorised as threatened or near-threatened by the International Union for Conservation of Nature (IUCN). LSU occupancy and SSU occupancy provide mean posterior estimates in the two datasets for the fraction of sites occupied at Ailaoshan (95% Bayesian confidence intervals in parentheses). Dashes indicate species that were not detected in one of the two datasets. Taxonomic information and IUCN Red List category are based on classification generated by PROTAX. Supplementary Data 1 provides a complete list of species.

*IUCN categories: NT* near threatened, *EN* endangered, *VU* vulnerable.

Leech iDNA appeared more successful at detecting Ailaoshan’s mammals and amphibians than its birds and squamates, based on our comparison with species lists from the Kunming Institute of Zoology (Supplementary Data 2). Among mammals, 34 of the 127 species in Ailaoshan were detected, with nearly half the detections in the larger-bodied orders: Artiodactyla (8 of 11 species), Carnivora (7 of 18), and non-human primates (1 of 4). Of the smaller-bodied orders, we detected 14 of 41 Rodentia species (including two porcupine species, *Atherurus macrourus* and *Hystrix brachyura*), 2 of 24 Eulipotyphla species (shrews and allies), and no bats (0 of 25), rabbits (0 of 1), pangolins (0 of 1) or treeshrews (0 of 1). We also detected two unnamed species assigned to Rodentia. Among amphibians, 12 of the 25 frog species (order Anura) known from Ailaoshan were detected, and so were both of the salamander species (family Salamandridae). We detected 13 more anuran species that could not be assigned to species, including two assigned to the genus *Kurixalus*, which has not been reported from Ailaoshan but which has a distribution that overlaps Yunnan (Supplementary Data 3). Among squamates, we detected only 3 unnamed species, compared to 39 species known from Ailaoshan. One of our species was assigned only to Squamata, and the others to families Scincidae and Viperidae respectively. Finally, among birds, 12 of the 462 bird species known from Ailaoshan were detected, plus 10 more species that were assigned to genus or higher. Interestingly, of the 12 species identified to species level, five are in the ground-feeding and terrestrial Phasianidae (pheasants and allies), out of 14 species known from Ailaoshan, and the other seven are known to be part-time ground and understorey feeders. Given that our LSU and SSU primers both had high amplification success *B*_*c*_ for mammals and birds (see *Laboratory Processing* in the Methods section), we tentatively attribute the difference in detection rates to the leeches – which were predominantly collected by rangers at ground level – having been more likely to have parasitised frogs than non-ground-feeding birds.

The most common taxa had occupancy estimates of around 0.6 in the LSU dataset and 0.8 in the SSU dataset (Tables 1 and 2). Most taxa, however, were observed infrequently (median number of detections: 2 and 3 patrol areas in the LSU and SSU datasets, respectively). This was reflected in low occupancy and detection estimates for many taxa (Fig. 2c) (median fraction of sites occupied: 0.33 and 0.24 in LSU and SSU, respectively; median detection probability per 100 leeches: 0.02 and 0.08 in LSU and SSU, respectively).

### Species richness

Per patrol area, estimated median species richness was 32 in the LSU dataset and 27 in the SSU dataset, compared to observed median species richnesses of 3 and 4 species per patrol area respectively (Supplementary Fig. 3a, b). Per replicate, observed median species richness was 1 and 2 in the LSU and SSU datasets respectively, from a median of 3 and 4 replicates per patrol area in each dataset.

**Fig. 3:**
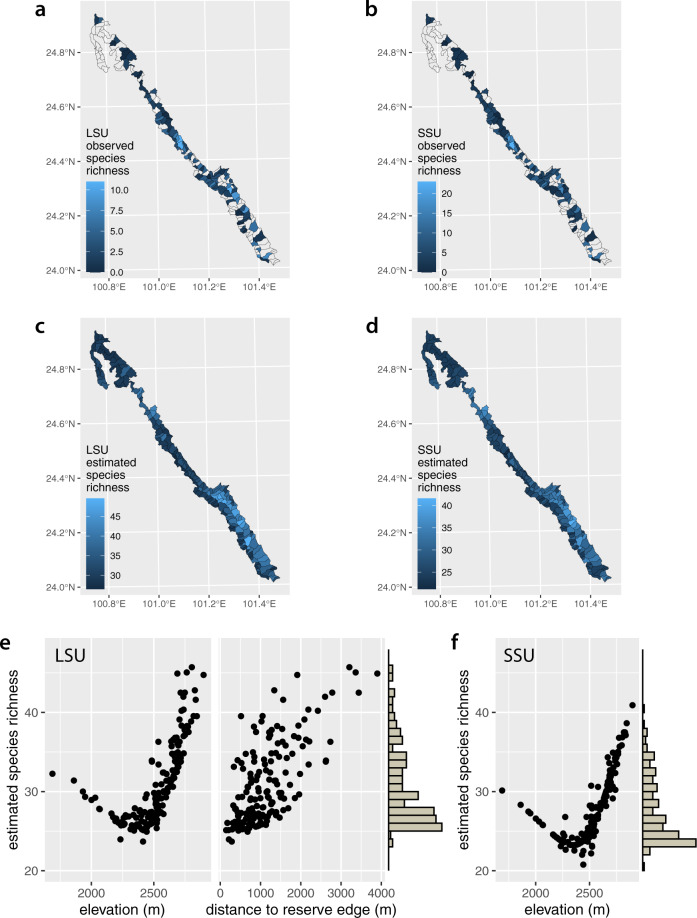
Species richness by patrol area. **a**, **b** Observed species richness in each patrol area in the LSU and SSU datasets respectively. Note missing data (no shading) in approximately half of the patrol areas. Data with missing patrol area IDs are not represented in this figure, though they are incorporated in our occupancy model. **c**, **d** Estimated species richness for each patrol area in the LSU and SSU datasets respectively. Note that our occupancy model provides estimates for patrol areas with missing data, in addition to augmenting observed values to account for false negatives. **e**, **f** Scatterplots of estimated species richness against environmental covariates in the LSU and SSU models respectively. Histograms along the *y*-axes show the distribution of species richness estimates across the patrol areas.

The substantial gap between observed and estimated species richness per patrol area in both datasets highlights the extent to which imperfect detection of vertebrate species may bias biodiversity estimates. Although estimated detection varied widely among species, most species had very low detection probabilities, especially in replicates containing few leeches (Fig. 3c–f). These results underscore the importance of correcting for false negatives when using iDNA to conduct biodiversity surveys.

Almost half of all patrol areas had no associated species observations, either because they were not sampled, or because samples were inadequately labelled (Fig. 3a, b; though note that this map does not display samples without location information, which were still used as data in our model). Our occupancy models impute missing data and therefore provided species-richness estimates for all patrol areas, both with and without observed values (Fig. 3c, d). Both datasets indicated that species richness is highest in the southern third of the Ailaoshan reserve.

**Fig. 4:**
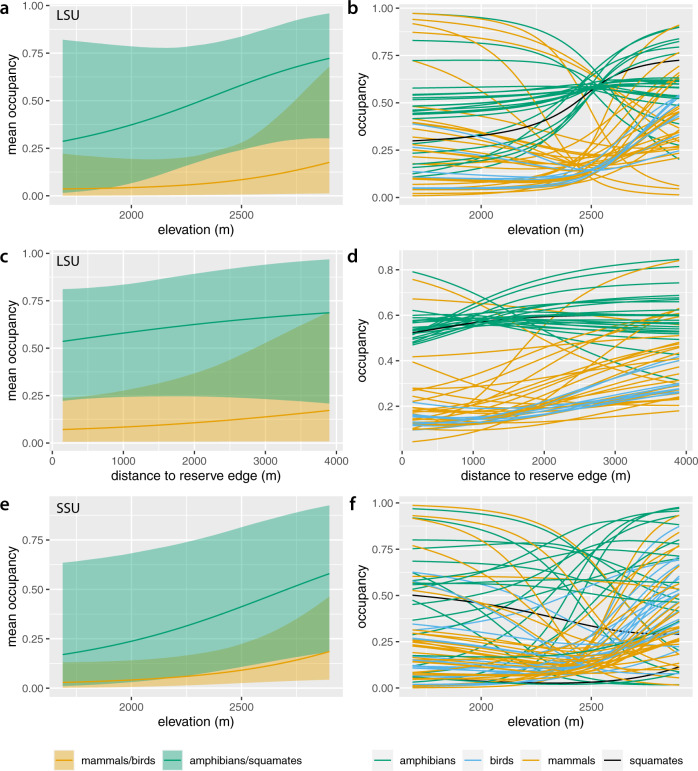
Occupancy estimates versus environmental covariates. **a** Community mean occupancy estimates and **b** occupancy estimates for each species as a function of elevation in the LSU dataset, holding distance to reserve edge fixed at its mean value. **c** Community mean occupancy estimates and **d** occupancy estimates for each species as a function of distance to reserve edge in the LSU dataset, holding elevation fixed at its mean value. **e** Community mean occupancy estimates and **f** occupancy estimates for each species as a function of elevation in the SSU dataset, holding distance to reserve edge fixed at its mean value. Lines in all panels show posterior means. Shaded areas in panels **a**, **c** and **e** show 95% Bayesian confidence intervals from models based on *n* = 893 replicate samples.

At the community level, species were more likely to occur at higher elevation and (to a lesser extent) further from the reserve edge. This can be seen in two ways. Firstly, estimated species richness in the reserve increased with elevation (both datasets) and with distance to reserve edge (LSU dataset) (Fig. 3e, f). Secondly, community mean occupancy (Eqs. (11) and (12)) increased with elevation in both datasets, holding distance to reserve edge constant in the LSU dataset (Fig. 4a, e). On the other hand, community mean occupancy showed limited increase with distance to reserve edge in the LSU dataset, with elevation held constant (Fig. 4c).

There was good agreement on species richness between the LSU and SSU datasets. Observed species richness in the two datasets was positively correlated at the grain of individual replicates (Supplementary Fig. 4a) and of patrol areas (Supplementary Fig. 4c). Unsurprisingly, estimated species richness was also tightly and positively correlated between the two datasets (Supplementary Fig. 4e). Sampling effort increased species detections: replicates with more leeches tended to contain more species (Supplementary Fig. 4b), as did patrol areas with more replicates (Supplementary Fig. 4d). However, as expected, estimated species richness did not increase with sampling effort, because our model compensates for variation in leech quantity and replicate number (Supplementary Fig. 4f).

At the species level, the effects of elevation (both datasets) and distance to reserve edge (LSU only) varied in both direction and strength (Fig. 4b, d, f). Among mammals over 10 kg, domestic cow (*B. taurus*), domestic sheep (*O. aries*), domestic goat (*C. hircus*) and muntjak (*Muntiacus vaginalis*) showed decreasing occupancy probability with elevation (Supplementary Figs. 5 and 7). Lower elevation sites in turn tend to be closer to the reserve edge; however, as for community mean occupancy, the independent effect of distance to reserve edge was small (Supplementary Fig. 6). In contrast, species such as tufted deer (*Elaphodus cephalophus*), sambar (*R. unicolor*), serow (*C. milneedwardsii*), Asiatic black bear (*U. thibetanus*) and wild boar (*Sus scrofa*) showed increasing occupancy probability with elevation and were thus more likely to occur in higher-elevation forest toward the centre of the reserve (Supplementary Figs. 5 and 7).

Most species of mammal below 10 kg were also estimated to have greater occupancy in more central, higher-elevation forest, including the Asian red-cheeked squirrel (*Dremomys rufigenis*) and the shrew gymnure (*Neotetracus sinensis*) (Supplementary Figs. 5 and 7). Birds likewise tended to have higher occupancy in higher elevation sites. On the other hand, a few small-mammal species such as the Himalayan field rat (*Rattus nitidus*) fared better in reserve-edge, lower-elevation forest. Amphibians showed a mix of responses, with some species such as the Tonkin toad (*Bufo pageoti*; IUCN Near Threatened) and the Jingdong toothed toad (*O. jingdongensis*; IUCN Vulnerable) more common in less accessible areas at higher elevations, but others such as the fire-bellied toad (*Bombina maxima*) more common in reserve-edge, lower-elevation forest.

### Community composition

**Fig. 5:**
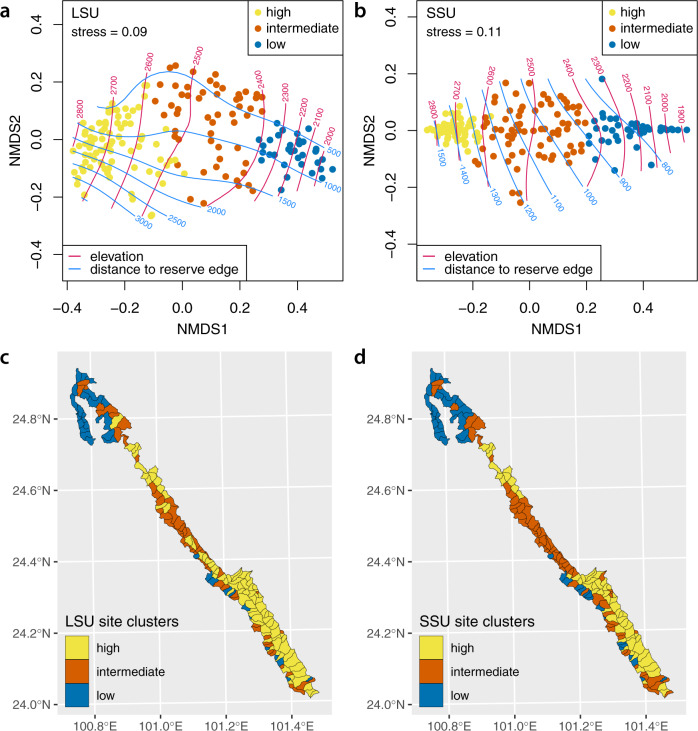
Vertebrate community composition by patrol area. **a**, **b** Non-metric multidimensional scaling plots representing mean pairwise Jaccard distances among patrol areas. Each point represents a single patrol area, coloured according to the cluster that it falls into (see Supplementary Fig. 8). Red and blue contours show elevation and distance to the reserve edge respectively (both in metres). Clusters correspond broadly to high-, intermediate- and low-elevation sites. **c**, **d** Maps showing distribution of clusters across the Ailaoshan reserve.

In both datasets, hierarchical clustering separated patrol areas into three groups, corresponding to low-, intermediate- and high-elevation sites (Fig. 5a, b and Supplementary Fig. 8). These groups of sites were highly congruent across the two datasets (Cramer’s *V* = 0.79, 95% confidence interval 0.73–0.85). The higher-elevation areas tend to be located in the interior of the reserve, especially in the south, and contain larger amounts of relatively inaccessible forest compared to lower-elevation areas (Supplementary Fig. 1a, i; mean ± s.d. distance to reserve edge 1540 m ± 850 m for top quartile of sites by elevation, compared to 830 m ± 390 m for the bottom quartile).

**Fig. 6:**
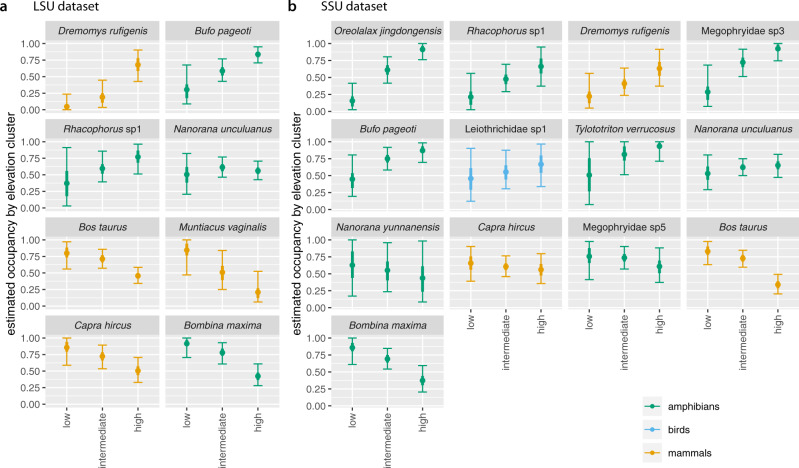
Occupancy for selected species by site cluster. Estimated occupancy in low-, intermediate- and high-elevation patrol areas for selected species in **a** the LSU dataset and **b** the SSU dataset. For each species, figure shows posterior mean (dot), interquartile range (thick line) and 95% Bayesian confidence interval (BCI; thin line with crossbars) for fraction of sites occupied from models based on *n* = 893 replicate samples. Patrol areas were divided into low-, intermediate- and high-elevation by clustering based on posterior mean Jaccard distances as shown in Fig. 5 and Supplementary Fig. 8. Species shown are those with posterior mean occupancy ≥0.4 and posterior mean detection ≥0.1 calculated across all patrol areas. Results for all species are shown in Supplementary Figs. 9 and 10.

Communities in low-elevation patrol areas were strongly characterised by the presence of domestic cow (*B. taurus*), domestic goat (*C. hircus*), muntjak (*M. vaginalis*) and fire-bellied toad (*B. maxima*) (Fig. 6). These species were present in the majority of low-elevation sites, but less than half of the high-elevation sites. In contrast, the Tonkin toad (*B. pageoti*) and Jingdong toothed toad (*O. jingdongensis*) showed the reverse pattern: i.e. they were absent from most of the low-elevation sites, but present in most of the high-elevation patrol areas. Indeed, many amphibians and birds occupied a larger fraction of high-elevation sites than of low-elevation sites (Supplementary Figs. 9 and 10). Nonetheless, some species, such as the Yunnan Asian frog (*N. unculuanus*), showed similar site occupancy across low-, intermediate- and high-elevation sites (Fig. 6).

Comparing the variation in composition among sites across the two datasets revealed significant co-inertia (RV coefficient^54^ 0.77, *p* ≤ 0.001), indicating that there was substantial shared signal in the two datasets. The Jaccard distances from the two datasets were also highly correlated (Pearson correlation *r* = 0.94, *p* = 0.001).

## Discussion

Here we demonstrate that metabarcoding of leech-derived iDNA permits large-scale, spatially-resolved estimation of vertebrate biodiversity. Our study is both the most granular and the broadest-scale biodiversity survey using iDNA to date. Leech surveys were conducted by untrained forest rangers for only 2–3 months and captured distribution information on mammals and amphibians, and to a lesser extent birds and squamates, across a topographically challenging, 677 km^2^ nature reserve (Fig. 1). Our results show that the Ailaoshan reserve provides protected space for vertebrate species of high conservation value, mostly in its core area. The results also highlight the vulnerability of the reserve to degradation arising from human activity (e.g. farming, livestock, and poaching) (Figs. 3 and 5). The study provides an iDNA vertebrate biodiversity baseline for Ailaoshan, and future iDNA surveys can test for changes in occupancy as a proxy for effectiveness^16^. More generally, our study functions as a progress report on the use of iDNA monitoring in real-world management settings, and highlights areas for improvement going forward.

### Vertebrate biodiversity in Ailaoshan

Our iDNA survey recovered 86 species of mammals, amphibians, birds and squamates, plus humans. Many were common wildlife species, or domesticated taxa such as cattle. The dataset also included many less common taxa that would have not been detected without targeted, taxon-specific traditional surveys, including 15 species recognised by the IUCN as Near Threatened or Threatened (Table 3).

Occupancy modelling indicated that vertebrate species richness was greatest in the higher-elevation interior of Ailaoshan. Our result likely reflects greater anthropogenic disturbance (e.g. hunting, disease transmitted from domestic animals to wildlife, and habitat alteration) in the lower, more-accessible parts of the park, causing local extinctions of many wildlife species at lower elevations. Alternatively, more mobile species may have shifted their home ranges from their previously-preferred lower-elevation areas to less suitable habitat to escape human encroachment^19^.

Elevation and distance to reserve edge were important predictors of vertebrate community richness and composition (Figs. 3e, f and 5a, b). Examining the distribution of individual taxa revealed that many species, especially birds and small mammals, had higher occupancy at higher elevation and in the reserve core area. These species include several that are IUCN Near-Threatened or Threatened species: stump-tailed macaque (*Macaca arctoides*), tufted deer (*E. cephalophus*), sambar (*R. unicolor*), serow (*C. milneedwardsii*) and Asiatic black bear (*U. thibetanus*). Some or all of these species are sensitive to habitat alteration along the reserve edge, poaching, competition with domestic animals (e.g. most ungulates), and/or may be prone to human-wildlife conflict (e.g. Asiatic black bear) in peripheral areas of the reserve, which are used heavily by livestock. In contrast, a few wild species, like the northern red muntjak (*M. vaginalis*), appear to have increased occupancy in reserve-edge areas.

### Using iDNA for biodiversity monitoring

Two key benefits of leech-iDNA surveys are (a) the ability to survey a wider range of vertebrate taxa and body sizes than is possible with other methods and (b) the feasibility of engaging large numbers of minimally-trained personnel for sampling and data collection. This results in time and cost savings, and makes regular broad-scale surveys more feasible. However, these benefits are partly offset by a greater laboratory workload (which could be mitigated by automation); challenges over the design of sampling incentives (see below); iDNA-specific sampling errors and biases; and the workload associated with bioinformatic processing and statistical modelling. We required 12 person-months to count the leeches, extract DNA, and run PCRs, and Novogene required one month to construct libraries and carry out sequencing. The consumables cost of DNA extraction, PCR, and sequencing was around RMB 210,000 (USD 30,000), with an additional RMB 80,000 (USD 12,000) for primers sufficient to run several surveys of this size.

#### Design of sampling incentives

Sampling with the assistance of forest rangers proved to be a feasible way to collect large numbers of leeches across the entire reserve. Rangers were hired locally from villages neighbouring the park. They did not report to a central location; instead, forestry officials brought boxes of hip packs to groups of rangers at locations around the park in June-July 2016, issued instructions verbally, and retrieved the packs after surveys ended in September. Provisioning the packs with tubes distributed over multiple self-sealing bags naturally enforced replicate sampling with minimal explanation^23^. This made it feasible for replicates from each patrol area to be collected at a single time point, removing the possibility that occupancy might change between temporal replicates^30^. However, for logistical reasons, collections from different patrol areas took place over a period of three months.

Collection of metadata, however, was less successful, as many samples had information on the collecting ranger but not the patrol area. In future sampling, metadata submission could be made a condition of payment, and a subset of senior rangers should be trained on metadata collection. A longer-term possibility is to outfit rangers with a GPS-enabled app on their cell phones for collecting coordinates of collection sites. On the other hand, our occupancy modelling framework deals well with moderate amounts of missing data, and we are wary of creating incentives to fabricate information. For instance, we decided against paying on a per-leech or per-tube basis, because this might incentivize rangers to collect outside the reserve. We found that a fixed payment, plus a small bonus for at least one leech collected, worked well, and we have since used this structure in other rounds of leech sampling. We expect to need to increase future payments.

#### Error and bias in iDNA sampling

There are several potential sources of error in our study. One is the time between a leech’s last feed and our sampling, which could be up to a few months^49^. While the retention of blood meal DNA facilitates detection of animals, it also means that detected DNA does not necessarily reflect occupancy at the time of leech surveys. Animal hosts may leave the patrol area between the feeding event and our sampling, and even leeches may disperse widely if carried on hosts such as birds that can travel long distances^55^, potentially blurring the spatio-temporal resolution of occupancy results. Our data show that the leeches we collected mostly feed on hosts that probably remain within one patrol area or, at most, move between adjacent areas (e.g. frogs), so our broad conclusions about the overall distributions of wild and domesticated species in Ailaoshan (Figs. 3 and 5) are unlikely to be seriously affected by this bias. Further, the collection of all replicate samples from a location within the three-month window limits the potential for leech or host movements to violate the site-occupancy model assumption that species occupancy remains constant across replicates (i.e. the ‘population closure’ assumption^23,56^). Nonetheless, the lag time restricts the suitability of leech iDNA for detecting very rapid change, e.g. occurring on the order of a few months^23^.

A second source of error could be systematic differences across patrol areas in leech communities, coupled with differing diet preferences among leech species. For instance, if leech species differ with elevation (which we did not include as a detection covariate), and high-elevation leech species tend to feed more on frogs and less on cattle, this would give the appearance of change in these species’ occupancy with elevation. The large number of leeches in our sample made it infeasible to identify them individually, but the geographic location of our field site and the uniform morphology of the leeches is consistent with all the leeches being in the genus *Haemadipsa*^28^, the taxonomy of which is poorly resolved. *Haemadipsa* are known to feed on a wide range of vertebrate species^27,28^, probably because they are opportunistic, sit-and-wait parasites, and studies suggest at most limited evidence for dietary differences^24,28,30^. Given this, we opted for a protocol that pooled leeches rather than attempting to take individual leech identity and diet into account, and we do not think it likely that differences in leech diet are likely to account for any of the major results in our study.

A third possible source of error is the choice of PCR primers and genetic markers, which may prevent some taxa from being detected even when their DNA is present, e.g. due to non-amplification at the PCR stage. We addressed this problem in part by using data from two marker genes. More than half of the species were detected by both markers, and high correlation in species richness and co-inertia of community composition between the datasets suggested that broad ecological inferences would not have been strongly affected had either marker been chosen by itself (Figs. 3 and 5). On the other hand, the primers clearly differed in their ability to amplify DNA from certain species. For example, we detected the stump-tailed macaque (*M. arctoides*) in the LSU dataset in three different patrol areas, with 2700, 170,066, and 245,477 reads. In contrast, there was no obvious SSU equivalent, with no OTUs (other than humans) assigned to the order Primates in the SSU dataset. Using additional primers would likely detect further taxa^57^, albeit with diminishing return on the additional sequencing costs. In the future, the use of nucleic-acid baits and/or metagenomic sequencing^58^, or the new CARMEN method that multiplexes CRISPR-Cas13 detection^59^, may replace PCR. Either approach could allow, for example, the use of the cytochrome *c* oxidase I (COI) barcode sequence, for which databases are more extensive^60^, while also allowing other genetic markers to be used for taxonomic groups that are not well distinguished by COI.

Finally, leech iDNA will naturally exclude taxa that are not well represented in leech blood meals. Studies have reported lower iDNA detection rates for many species compared to camera trapping, though iDNA appears to be better at detecting smaller-bodied species of mammal^19,31,32,49,61^ and, in our study, amphibians. With sufficiently large samples, taxa that are present infrequently may still be detected, and their low detection rates accounted for using site-occupancy modelling. Taxa that are never detected can still be modelled statistically (e.g. using data augmentation^46,53^), but they obviously cannot contribute data towards the model. When leech sampling is the rate-limiting step, such as in researcher-led studies, Abrams et al.^30^ recommend using leech-iDNA to supplement camera-trap data. For instance, Tilker et al.^19^ recently ran a camera-trap survey at 139 stations (17,393 trap-nights) over five protected areas in Vietnam and Laos, spanning 900 km^2^, and supplemented the camera data with iDNA from 2043 leeches from 93 of the stations. The camera-trap data were limited to 23 terrestrial mammal species, with squirrels and large rodents being the smallest organisms detected, and generally produced more species detections. However, leech iDNA provided the sole detections of marbled cat (*Pardofelis marmorata*), and doubled the detections of Owston’s civet (*Chrotogale owstoni*) and Asiatic black bear (*U. thibetanus*). On the other hand, broad ecological patterns may still be identified without necessarily detecting every species present in an area. For example, Gogarten et al. found that camera trapping and fly-derived iDNA detected largely non-overlapping communities (only 6% to 43% of species were found by both methods in any given location)^61^, but both methods tended to classify habitats similarly.

#### Multi-species site-occupancy modelling

Site occupancy modelling identified correlates of detection and occupancy at the level of the community as well as individual species. Most taxa were detected infrequently, and individually, they provided little insight into detection and occupancy rates, as it is difficult to distinguish low detection rates (i.e. crypsis) from low occupancy (i.e. rarity). However, by integrating these infrequent detections into community models of occupancy and detection, and sharing information across species and patrol areas, the entire dataset was able to produce a broad picture of vertebrate diversity across Ailaoshan. This modelling approach dealt well with missing data, demonstrating the usefulness of occupancy models in a Bayesian framework for dealing with the imperfect datasets that are to be expected with surveys across broad areas and relying on limited resources. On the other hand, the data-augmented models represented a substantial computational burden with our large dataset, with high memory requirements, long run times and much experimentation required to fit the models successfully.

While in this study we focused our modelling attention on correcting for false negatives, false positives are also possible, e.g. due to lab contamination or taxonomic misassignment. While false negatives are likely to be a more serious problem than false positives in our dataset, false positives may nonetheless cause serious bias in the estimation of biodiversity^62^. Hierarchical models may, in principle, also be used to correct for false positives, but in practice they have proven challenging to estimate without additional information about the false-positive detection process^63^. Recent advances in modelling false positives show promise (e.g. Griffin et al.^64^), but these approaches are not yet available for multi-species metabarcoding datasets.

As iDNA surveys are increasingly used for large-scale studies, an important study design consideration will be the degree to which leeches are pooled. Pooling reduces the cost and complexity of the collecting task, since putting leeches into individual tubes requires a larger collecting kit. (Leeches regurgitate into the preservative fluid, such that leeches collected into the same tube cannot be treated as independent replicates; separate tubes for individual leeches would be needed.) Pooling also reduces lab costs and workload. On the other hand, occupancy models such as the one employed here work best when provided with data from unpooled samples. Potentially valuable information about leech host preferences is also lost when samples are pooled: for example, if collected individually, leeches could be DNA-barcoded, and this information used as a detection covariate in occupancy modelling. Development of automated, high-throughput laboratory protocols (e.g. Ackerman et al.^59^) would help make individual sequencing of leeches more practical in large sample sets such as ours (i.e. >30,000 individuals). At the collection stage, a compromise could be to issue collectors with smaller collecting tubes than we used (e.g. 2 mL), in order to lower leech numbers per replicate but not necessarily to the level of individual leeches.

### iDNA: a promising biodiversity monitoring tool

As we prepare to replace the Aichi Biodiversity Targets with a new post-2020 framework, there has been a call to focus on directly evaluating conservation outcomes using biodiversity measures such as occupancy, abundance, and population trends^4,65,66^. However, many protected areas are under-resourced and under-staffed^2^, and biodiversity monitoring may be difficult to prioritise^4^. In this study, we show the feasibility of using iDNA metabarcoding as a cost-effective way to estimate spatially-resolved vertebrate occupancies across entire protected areas and with broad taxonomic coverage. Our work thus demonstrates the potential for iDNA to facilitate direct measurements of biodiversity conservation outcomes.

In addition to yielding occupancy estimates, our work can also guide future monitoring to identify underlying sources of environmental change, anthropogenic influences, and overall wildlife community dynamics. We recommend using our results to guide the design of targeted scat-collection, camera-trap, and bioacoustic monitoring surveys of Ailaoshan, both to independently test our results with species that are amenable to being recorded with these other methods (e.g. mammals, ground-dwelling birds), and to improve the accuracy of occupancy and detection estimates^30^. These monitoring methods could also be used to estimate population sizes and population trends for some species using an occupancy modelling framework^67–69^. We further propose that iDNA may be used to survey other dimensions of biodiversity, such as zoonotic disease. Recent work has demonstrated the exciting possibility of using leech-derived bloodmeals, sampled from the wild, to screen for both viruses and their vertebrate hosts^29,70^. The 2020 SARS-CoV-2 pandemic has underscored the urgency of better understanding zoonotic disease in wildlife reservoirs – a need that is likely to become even more pressing as global climate and land use changes continue^71^.

## Methods

This section provides an overview of methods. The Supplementary Information provides additional detailed descriptions of the leech collections, laboratory processing, bioinformatics pipeline, and site-occupancy modelling. Code for our bioinformatics pipeline is available at Ji^72^ and Yu^73^. Code for our site-occupancy modelling and analysis is available at Baker et al.^74^.

### Leech collections

Samples were collected during the rainy season, from July to September 2016, by park rangers from the Ailaoshan Forestry Bureau. The nature reserve is divided into 172 non-overlapping patrol areas defined by the Yunnan Forestry Survey and Planning Institute. These areas range in size from 0.5 to 12.5 km^2^ (mean 3.9 ± sd 2.5 km^2^), in part reflecting accessibility (smaller areas tend to be more rugged). These patrol areas pre-existed our study, and are used in the administration of the reserve. The reserve is divided into six parts, which are managed by six cities or autonomous counties (NanHua, ChuXiong, JingDong, ZhenYuan, ShuangBai, XinPing) which assign patrol areas to the villages within their jurisdiction based on proximity. The villages establish working groups to carry out work within the patrol areas. Thus, individual park rangers might change every year, but the patrol areas and the villages responsible for them are fixed.

Each ranger was supplied with several small bags containing tubes filled with RNAlater preservative. Rangers were asked to place any leeches they could collect opportunistically during their patrols (e.g. from the ground or clothing) into the tubes, in exchange for a one-off payment of RMB 300 ( ~USD 45) for participation, plus RMB 100 if they caught one or more leeches. Multiple leeches could be placed into each tube, but the small tube sizes generally required the rangers to use multiple tubes for their collections.

A total of 30,468 leeches were collected in 3 months by 163 rangers across all 172 patrol areas. When a bag of tubes contained <100 total leeches, we reduced our DNA-extraction workload by pooling leeches from all tubes in the same plastic bag and treating them as one replicate. However, when a bag contained ≥100 total leeches, we selectively pooled some of the tubes in that bag to create five approximately equally sized replicates from the bag, to avoid any replicates containing an excessive number of leeches. Eighty-one per cent of bags contained <100 leeches, and 78% of patrol areas consisted only of bags below the threshold. Each patrol area typically returned multiple replicates, in the form of multiple bags below the threshold and/or multiple tubes from the bags above the threshold. After this pooling, the mean number of leeches per replicate was 34 (range 1–98), for a total of 893 replicates across the entire collection.

### Environmental characteristics

We used ArcGIS Desktop 9.3 (Esri, Redlands, CA) and R v3.4.0^75^ to calculate characteristics of each patrol area. We created 30 m raster layers for elevation, topographic position index (i.e. difference between each pixel and its surrounding pixels^76^), distance to nearest road, and distance to nearest stream. We then calculated the median of the raster values for each patrol area for use as predictors in our statistical modelling (Table 4 and Supplementary Fig. 1). We also calculated distance to the Ailaoshan reserve edge as the distance of each patrol-area centroid to the nearest nature-reserve edge.

**Table 4:** Summary of environmental covariates.

Variable	Description	Mean ± SD	Min	Max
Elevation	Median elevation (m)	2510 ± 210	1690	2900
TPI	Median topographic position index	0.6 ± 3.5	−12.0	20.0
Road	Median distance to road (m)	840 ± 640	60	2870
Stream	Median distance to stream (m)	360 ± 180	90	1010
Reserve	Centroid distance to reserve edge (m)	1110 ± 670	150	3900

### Laboratory processing

We extracted DNA from each replicate and then PCR-amplified two mitochondrial markers: one from the 16S rRNA gene (*MT-RNR2*; primers: *16Smam1* 5′-CGGTTGGGGTGACCTCGGA-3′ and *16Smam2* 5′-GCTGTTATCCCTAGGGTAACT-3′^77^), and the other from the 12S rRNA gene (*MT-RNR1*; primers: 5′-ACTGGGATTAGATACCCC-3′ and 5′-YRGAACAGGCTCCTCTAG-3′ modified from Riaz et al.^78^). We refer to these two markers as LSU (16S, 82–150 bp) and SSU (12S, 81–117 bp), respectively, referring to the ribosomal large subunit and small subunit that these genes code for. A third primer pair targeting the standard cytochrome *c* oxidase I marker^79^ was tested but not adopted, as it co-amplified leech DNA and consequently returned few vertebrate reads.

The LSU primers are designed to target mammals, and the SSU primers to amplify all vertebrates. We ran ecoPCR v0.5^80^ with three allowed mismatches on the Tetrapoda in the MIDORI database^81^ to estimate expected amplification success, *B*_*c*_, for our primers. *B*_*c*_ is the proportion of species in the reference database that can be amplified in silico. The *16Smam* primers returned high *B*_*c*_ values for Mammalia (99.3%), as expected, and also for Aves (96.2%), a moderate value for Amphibia (79%), and a low value for species grouped under “Reptilia" in the MIDORI database (=Crocodylia + Sphenodontia + Squamata + Testudines) (39.9%). The 12S primers returned high *B*_*c*_ values ( > 98%) for Mammalia, Amphibia, and Aves, and a moderate *B*_*c*_ value (79.8%) for “Reptilia”. We therefore expected most or all Ailaoshan mammals, birds, and amphibians to be amplifiable by one or both primers, and a lower success rate for snakes and lizards.

Primers were ordered with sample-identifying tag sequences, and we used a twin-tagging strategy to identify and remove ‘tag jumping’ errors^82^ using the DAMe protocol^83^. From our 893 replicate tubes, we successfully PCR-amplified in triplicate 661 samples using our LSU primers and 745 samples using our SSU primers. Successful PCR amplifications were sent to Novogene (Beijing, China) for PCR-free library construction and 150 bp paired-end sequencing on an Illumina HiSeq X Ten.

Negative controls were included for each set of PCRs, and the PCR set was repeated, or ultimately abandoned, if agarose gels revealed contamination in the negative controls. We also sequenced the negative controls, because gels do not always detect very low levels of contamination. Sequences assigned to human, cow, dog, goat, pig, chicken and some wild species appeared in our sequenced negative controls, but with low PCR replication and at low read number. We used these negative controls to set DAMe filtering stringency in our bioinformatics pipeline (see next section and Supplementary Information) for all samples to levels that removed these contaminants: -y 2 for both markers (minimum number of PCRs out of 3 in which a unique read must be present), and -t 9 for LSU and -t 20 for SSU (minimum number of copies per PCR at which a unique read must appear). We also amplified and sequenced a set of positive controls containing DNA from two rodent species, *Myodes glareolus* and *Apodemus flavicollis*, along with negative controls that we verified to be contamination-free using agarose gel electrophoresis. *M. glareolus* and *A. flavicollis* have European and Western Asian distributions, and we did not detect either species in our leech samples.

### Bioinformatics pipeline

The three key features of our bioinformatics pipeline were the DAMe protocol^83^, which uses twin-tagging and three independent PCR replicates to identify and remove tag-jumped and erroneous reads, the use of two independent markers, which provides an independent check on taxonomic assignments (Supplementary Fig. 2), and the PROTAX statistical ‘wrapper’ for taxonomic assignment^84,85^, which reduces overconfidence in taxonomic assignment when reference databases are incomplete, as they always are. In this case, around half of the known Ailaoshan taxa were present in the reference databases (Supplementary Data 2). Mammals and amphibians were relatively well represented: 73% of mammals and 83% of amphibians were in the LSU database, respectively 70% and 67% in the SSU database. Birds and squamates were less well captured, with 42% of birds and 53% of squamates present in the LSU database, respectively 35% and 34% in the SSU database. For OTUs that do not have reference sequences, PROTAX assigns them to higher ranks and flags them as ‘unknowns,’ allowing us to assign those OTUs to morphospecies and potentially supply taxonomy based on other information such as correlations between the datasets as described here.

After DAMe filtering, we removed residual chimeras using VSEARCH v2.9.0^86^, clustered sequences into preliminary operational taxonomic units (‘pre-OTUs’) using Swarm v2.0^87^, and then used the R package LULU v0.1.0^88^ to merge pre-OTUs with high similarity and distribution across samples. We then used PROTAX to assign taxonomy to representative sequences from the merged pre-OTUs^33,84,85^, in which we benefited from recent additions to the mitochondrial reference database for Southeast Asian mammals^89^. The full pipeline is described in detail in the Supplementary Information (*Assigning taxonomy to preliminary operational taxonomic units* and following sections). We shared taxonomic information between the LSU and SSU datasets by making use of correlations between the datasets. To do this, we calculated pairwise correlations of LSU and SSU pre-OTUs across the 619 replicates for which both markers had been amplified and visualised the correlations as a network (Supplementary Fig. 2). If an LSU and an SSU pre-OTU occurred in (mostly) the same subset of replicates and were assigned the same higher-level taxonomies, the two pre-OTUs were deemed likely to have been amplified from the same set of leeches feeding on the same species. We manually inspected the network diagram and assigned such correlated pre-OTU pairs the same taxonomy.

We eliminated any pre-OTUs to which we were unable to assign a taxonomy; these pre-OTUs only accounted for 0.9% and 0.2% of reads in the LSU and SSU datasets respectively, and most likely represent sequencing errors rather than novel taxa. Within the LSU and SSU datasets, we merged pre-OTUs that had been assigned the same taxonomies, thus generating a final set of operational taxonomic units (OTUs) for each dataset. Finally, we removed the OTU identified as *Homo sapiens* from both datasets prior to analysis. Although it would be informative to map the distribution of humans across the reserve, we expect that most of the DNA came from the rangers themselves, not from other humans using the reserve.

Our final OTUs are intended to be interpreted as species-level groups, even though some cannot yet be assigned taxonomic names to species level (most likely due to incomplete reference databases). Thus, for example, the two frog OTUs *Kurixalus* sp1 and *Kurixalus* sp2 in the LSU dataset should be interpreted as two distinct *Kurixalus* species. Likewise, the frog OTU Megophryidae sp3 in the LSU and SSU datasets should be interpreted as a single species within Megophryidae. We therefore refer to our final OTUs as species throughout this study.

After excluding humans, the final LSU and SSU datasets comprised 18,502,593 and 84,951,011 reads respectively. These reads represented a total of 59 species across 653 replicates and 126 patrol areas in the LSU dataset, and 72 species across 740 replicates and 127 patrol areas in the SSU dataset. To assess the degree to which our iDNA approach was able to capture the breadth of vertebrate biodiversity in the park, we compared the list of species that we detected against unpublished, working species lists maintained by researchers at the Kunming Institute of Zoology.

We also attached additional metadata to our species list: we attached International Union for Conservation of Nature (IUCN) data for individual species by using the R package rredlist v0.6.0^90^ to search for scientific names assigned by PROTAX. For this purpose, we treated *Capricornis milneedwardsii* as synonymous with *Capricornis sumatraensis*, in line with recent research and the latest IUCN assessment^91,92^. For mammals, we used the PanTHERIA database^93^ to obtain data on adult body mass for each species; where species-level information was not available, we used the median adult body mass from the database for the lowest taxonomic group possible.

### Site-occupancy modelling

We estimated separate multispecies site-occupancy models for the LSU and SSU datasets using parameter-expanded data augmentation^46,53^. These models assume that the *n*_LSU_ = 59 and *n*_SSU_ = 72 species observed in each dataset are, respectively, subsets of larger communities of size *N*_LSU_ and *N*_SSU_ species that are present in the vicinity of Ailaoshan and vulnerable to capture (e.g. fed on by leeches and amplified by the LSU and SSU primers). Although *N*_LSU_ and *N*_SSU_ are unknown, these communities can be modelled by embedding them in a larger ‘supercommunity’ of fixed size *M*. We set *M* = 200 for our final model. Values from *M* = 150 up to *M* = 474 (the latter being the total species richness for mammals, birds, non-avian reptiles and amphibians in the 1984-85 survey of Ailaoshan^35^) produced similar estimates for *N*_LSU_ and *N*_SSU_.

For each species in the supercommunity, our models explicitly capture (i) a ‘community process’ governing whether the species is in the Ailaoshan community or not; (ii) an ‘ecological process’ governing the presence or absence of the species in each patrol area, given that it is in the community; and (iii) an ‘observation process’ governing whether we detect the species’ DNA in each of our replicate samples, given that it is present in the patrol area. The community-, ecological- and observation processes for individual species are linked by imposing community-level parameters and priors as described below.

For the community process, each species *i* was assumed to be either a member of the Ailaoshan community or not. We denote this unobserved state with *w*_*i*_, which was assumed to be a Bernoulli random variable governed by the community membership parameter ${{{\Omega }}}_{{g}_{i}}$, i.e. the probability that species *i* was in the Ailaoshan community:

1 $${w}_{i} \sim {{{{{{{\rm{Bernoulli}}}}}}}}({{{\Omega }}}_{{g}_{i}}).$$

For the community process, we separated the species into two natural groupings – homeothermic mammals and birds, and poikilothermic amphibians and squamates – and allowed them to have different probabilities of being in the Ailaoshan community. This is denoted by the subscript on the ${{{\Omega }}}_{{g}_{i}}$ parameter, in which *g*_*i*_ represents which of these two groupings species *i* belongs to. This approach reflected our expectation that these groupings would differ systematically in their community probabilities, and we employed the same grouping for parameters governing the ecological and detection processes (see below for further discussion).

For the ecological process, each species *i* was assumed to be either present or absent in each patrol area *j*, and we used *z*_*ij*_ to denote this unobserved ecological state. We assumed the *z*_*ij*_ to be constant across all replicates taken from patrol area *j*, consistent with the samples being taken at essentially the same point in time. Any species present were assumed to be members of the Ailaoshan community (i.e. *w*_*i*_ = 1), so we modelled *z*_*ij*_ as a Bernoulli random variable governed by both *w*_*i*_ and an occupancy parameter *ψ*_*ij*_, i.e. the probability that a species *i* in the community was present in patrol area *j*:

2 $${z}_{ij}| {w}_{i} \sim {{{{{{{\rm{Bernoulli}}}}}}}}({w}_{i}{\psi }_{ij}).$$

We modelled occupancy *ψ*_*ij*_ as a function of elevation and distance from the reserve edge in the LSU dataset

3 $${{{{{{{\rm{logit}}}}}}}}({\psi }_{ij})={\beta }_{0i}+{\beta }_{1i}{{{{{{{{\rm{elevation}}}}}}}}}_{j}+{\beta }_{2i}{{{{{{{{\rm{reserve}}}}}}}}}_{j}$$

and as a function of elevation in the SSU dataset

4 $${{{{{{{\rm{logit}}}}}}}}({\psi }_{ij})={\beta }_{0i}+{\beta }_{1i}{{{{{{{{\rm{elevation}}}}}}}}}_{j}$$

where elevation_*j*_ is the median elevation for patrol area *j*, and reserve_*j*_ is the distance from the centroid of patrol area *j* to the nature reserve edge. We chose these specifications by running a ‘full’ model for each dataset with all five environmental covariates, and retaining only those covariates for which the 95% Bayesian confidence interval on the slope coefficient excluded zero.

We modelled observation as a Bernoulli process assuming imperfect detection but no false positives:

5 $${y}_{ijk}| {z}_{ij} \sim {{{{{{{\rm{Bernoulli}}}}}}}}({z}_{ij}{p}_{ijk}),$$

where *y*_*ijk*_ is the observed data, i.e. detection or non-detection of species *i*’s DNA in replicate *k* from patrol area *j*.

We allowed the conditional detection probability *p*_*ijk*_ to vary as a function of the conditional detection probability for species *i* per 100 leeches, *r*_*i*_, and the number of leeches in the replicate, leeches_*jk*_:

6 $$\kern0.3pc {p}_{ijk}=1-{(1-{r}_{i})}^{{{{{{{{{\rm{leeches}}}}}}}}}_{jk}/100}$$

7 $${{{{{{{\rm{logit}}}}}}}}({r}_{i})={\gamma }_{0i}$$

We allowed *r*_*i*_, and its logit-scale equivalent *γ*_0*i*_, to vary among species to capture e.g. variation in leech feeding preferences among taxa. We used leeches_*jk*_/100 rather than leeches_*jk*_ to avoid computational problems arising from rounding.

Note that the detection probability *p*_*ijk*_ is conditional on species *i* being present in patrol area *j*, and not on species *i*’s DNA being present in replicate *k* from that site. *p*_*ijk*_ therefore subsumes multiple sources of imperfect detection, including those that result in species *i*’s DNA being absent from the replicate (e.g. the leeches in replicate *k* did not feed on species *i*, or they did so long ago and the DNA has since been digested), as well as those that result in apparent non-detection of species *i* DNA when it is present (e.g. failure to PCR amplify sufficiently, PCR or sequencing errors, or problems arising during bioinformatic processing). The multiple PCRs that we performed for each replicate (see *Laboratory processing* above, and Supplementary Information) could in principle have been used to decompose *p*_*ijk*_ into (i) a per-replicate probability that species *i*’s DNA is present in the replicate when the species is present at the site, and (ii) a per-PCR probability that species *i*’s DNA is detected when it present in the replicate, by adding another hierarchical level to our model^94–97^. However, we instead chose to combine the results from the multiple PCRs using DAMe^83^ prior to modelling, since DAMe is specifically designed to detect and remove errors arising in PCR and sequencing, and offers filtering options specialised to this task that we found useful.

Finally, whereas Eqs. (1) through (7) define a site-occupancy model for species *i* alone, we united these species-specific models with a community model for both ecological and detection processes:

8 $${\beta }_{1i} \sim {{{{{{{\rm{N}}}}}}}}({\mu }_{{\beta }_{1}},{\sigma }_{{\beta }_{1}})$$

9 $${\beta }_{2i} \sim {{{{{{{\rm{N}}}}}}}}({\mu }_{{\beta }_{2}},{\sigma }_{{\beta }_{2}})\quad ({{{{\rm{for}}}}\;{{{\rm{the}}}}\;{{{\rm{LSU}}}}\;{{{\rm{model}}}}\;{{{\rm{only}}}}})$$

10 $$({\beta }_{0i},{\gamma }_{0i}) \sim {{{{{{{\rm{MVN}}}}}}}}\left([{\mu }_{{\beta }_{0}{g}_{i}},{\mu }_{{\gamma }_{0}{g}_{i}}],\left[\begin{array}{cc}{\sigma }_{{\beta }_{0}{g}_{i}}^{2}&\rho {\sigma }_{{\beta }_{0}{g}_{i}}{\sigma }_{{\gamma }_{0}{g}_{i}}\\ \rho {\sigma }_{{\beta }_{0}{g}_{i}}{\sigma }_{{\gamma }_{0}{g}_{i}}&{\sigma }_{{\gamma }_{0}{g}_{i}}^{2}\end{array}\right]\right)$$

where N() and MVN() denote normal and multivariate normal distributions. These distributions were characterised by community hyperparameters *μ*_•_ and *σ*_•_, with separate distributions for each parameter as denoted by the first subscript. We used a multivariate normal prior for (*β*_0*i*_, *γ*_0*i*_) to allow non-zero covariance between species’ occupancy and detection probabilities, as we might expect if, for example, variation in abundance affects both probabilities^46^.

These community models allow rare species effectively to borrow information from more common ones, producing a better overall ensemble of parameter estimates, though at the cost of shrinkage on the individual parameters^46,98,99^. As for the community process described above, we separated the species into two groups – homeothermic mammals and birds, and poikilothermic amphibians and squamates – and allowed them to have different community distributions. This is denoted by the subscripts on the *μ*_•_ and *σ*_•_ community hyperparameters for the occupancy and detection intercepts, in which *g*_*i*_ represents which of these two groupings species *i* belongs to. This approach reflected our expectation that these groupings would differ systematically in occupancy probabilities (e.g. due to different habitat preferences) and in detection probabilities (e.g. due to different encounter rates with leeches, or leech feeding preferences). Alternative groupings could also be justified on biological grounds: for example, separating mammals and birds on the basis that many of the mammals are terrestrial while many of the birds are arboreal; or grouping birds and squamates together to better reflect phylogeny. Such alternative groupings did not perform well in our datasets, as most birds and squamates were observed too infrequently to provide much information on these groups by themselves, but this aspect of the model would be worth revisiting in future work.

We estimated our models using a Bayesian framework with JAGS v4.3.0^100^. We used 5 chains of 100,000 generations, including a burn-in of 50,000. We retained all rounds (i.e. without thinning) for the posterior sample, except for where we needed to save the *z* matrix for beta diversity and cluster occupancy calculations (see *Statistical analyses* below); memory limitations prevented us from retaining all posterior samples for the *z* matrix, and we thinned tenfold in order to make these calculations feasible. The Supplementary Information provides details of the prior distributions used for the model parameters. From the model results we calculated posterior means and quantiles for all model parameters of interest, as well as estimated species richness for each patrol area, and number of sites occupied for each species.

### Statistics

#### Species richness

For each dataset, we obtained estimates of overall species richness for Ailaoshan directly from the model, by summing the *w*_*i*_. To assess our choice of *M*, we compared these overall species richness estimates for *M* = 100, 150 and 200.

After examining occupancy and detection estimates for each species, we used histograms to visualise the distribution of estimated species richness per patrol area (obtained for each patrol area *j* by summing the *z*_*ij*_). We calculated median estimated species richness across the patrol areas for comparison with median observed species richness per patrol area and per replicate. We drew choropleths to visualise the spatial distribution of both observed and estimated species richness across the nature reserve.

We examined community mean occupancy and detection probabilities (see e.g. Section 11.7.2 in Kéry and Royle^101^) to help understand the effects of the site and sample covariates. For each species group *g* = 1, 2 (representing mammals/birds and amphibians/squamates, respectively), we calculated the posterior mean and 95% Bayesian confidence interval for community mean occupancy and detection as functions of the covariates:

11 $${\psi }_{g}({{{{{{{\rm{elevation}}}}}}}})=logi{t}^{-1}({\mu }_{{\beta }_{0}g}+{\mu }_{{\beta }_{1}}{{{{{{{\rm{elevation}}}}}}}})$$

12 $${\psi }_{g}({{{{{{{\rm{reserve}}}}}}}})=logi{t}^{-1}({\mu }_{{\beta }_{0}g}+{\mu }_{{\beta }_{2}}{{{{{{{\rm{reserve}}}}}}}})\quad ({{{{\rm{for}}}}\;{{{\rm{the}}}}\;{{{\rm{LSU}}}}\;{{{\rm{model}}}}\;{{{\rm{only}}}}})$$

13 $${p}_{g}({{{{{{{\rm{leeches}}}}}}}})=1-{(1-{{{{{{{{\rm{logit}}}}}}}}}^{-1}({\mu }_{{\gamma }_{0}g}))}^{{{{{{{{\rm{leeches}}}}}}}}/100}$$

This approach effectively holds distance from reserve edge at zero in *ψ*_*g*_(elevation), and elevation at zero in *ψ*_*g*_(reserve), corresponding to the mean values for these covariates in our data, since predictors were normalised prior to modelling. To visualise variation among species in occupancy and detection response to covariates, we repeated these calculations using each species’ estimates for *β*_0_, *β*_1_, *β*_2_ and *γ*_0_ in place of the community hyperparameters to obtain the posterior means for each species.

We compared three measures of species richness between the two datasets in order to assess the extent to which the two datasets agreed on variation in richness within Ailaoshan. First, the observed species richness in each replicate; second, the observed species richness in each patrol area; and third, the estimated species richness in each patrol area (i.e. the posterior mean number of species, calculated from *z*_*ij*_). For each of these measures, we computed the Pearson correlation between the datasets and tested the correlation coefficient against zero with a *t*-test. We also used Poisson GLMs to examine the relationship between each of these species richness measures and sampling effort: we regressed observed species richness per replicate against the log-transformed number of leeches per replicate, and we regressed both the observed and estimated species richness per patrol area against the log-transformed number of replicates per patrol area, testing the significance of the slope coefficients with *t*-tests.

#### Community composition

We explored variation in vertebrate community composition among patrol areas using posterior mean Jaccard similarities calculated from the estimated occupancy states *z*_*ij*_ (see Dorazio^53^ and Kéry and Royle^101^ for other examples of this approach). We visualised the pairwise Jaccard distances (i.e. distance = (1 − similarity)) using non-metric multidimensional scaling ordinations, overlaying environmental covariates using the vegan::ordisurf function. We clustered patrol areas based on the Jaccard distances using Ward’s criterion (R function hclust(., method = “ward.D2”)). We used this clustering to split the patrol areas into three groups, which turned out to correspond to low-, intermediate-, and high-elevation sites. We used Cramer’s *V* to quantify the extent to which these clusters matched across the two datasets. We visualised the spatial variation in community composition within the reserve by drawing maps of Ailaoshan with patrol areas coloured by these three clusters. To help understand how vertebrate communities varied among the clusters, we used the posterior sample of the occupancy states *z*_*ij*_ to calculate posterior means and 95% Bayesian confidence intervals for the occupancy (i.e. fraction of patrol areas occupied) of each species in the low-, intermediate- and high-elevation site clusters.

To assess the extent to which the two datasets identified common patterns of variation in community composition across the patrol areas, we performed a co-inertia analysis on the matrices of predicted species in each patrol area in each dataset using ade4::coinertia in R. We used the RV coefficient^54^ to quantify coinertia, testing its significance with the permutation test in ade4::RV.rtest with 999 permutations. We also tested for correlation between the posterior mean Jaccard distances from the two datasets using a Mantel test with 999 permutations.

### Reporting summary

Further information on research design is available in the Nature Research Reporting Summary linked to this article.

## Supplementary information

Supplementary Information

Description of Additional Supplementary Files

Supplementary Dataset 1

Supplementary Dataset 2

Supplementary Dataset 3

Supplementary Dataset 4

Supplementary Dataset 5

Supplementary Dataset 6

Reporting Summary

## Data Availability

The Illumina HiSeq/MiSeq read data generated in this study have been deposited in the NCBI Sequence Read Archive under BioProject accession number PRJNA624712. Processed data in the form of OTU- and metadata tables are provided as Supplementary Data 6, and are also included in the GitHub repository containing our occupancy modelling code (https://github.com/bakerccm/leeches-public/releases/tag/v1.1; 10.5281/zenodo.5914708). The MIDORI databases that we used are available from http://www.reference-midori.info. The mitogenomes from Mohd Salleh et al. 2017 (GigaScience 6(8): gix053) are available from GenBank under the accession numbers provided in Tables 1 and 2 of that publication (https://academic.oup.com/gigascience/article/6/8/gix053/3958782). The PanTHERIA database is available from 10.6084/m9.figshare.c.3301274.v1. Working species lists from Kunming Institute of Zoology researchers are provided in Supplementary Data 2 and 3.

## Code availability

Our pipeline for processing the Illumina read data is available at https://github.com/jiyinqiu/ailaoshan_leeches_method_code^72^. Bioinformatic scripts for processing the output of this pipeline, including taxonomic reference datasets, are available at https://github.com/dougwyu/screenforbio-mbc-ailaoshan/releases/tag/1.3^73^. The code for our analysis, including site occupancy modelling, is available at https://github.com/bakerccm/leeches-public/releases/tag/v1.1 (10.5281/zenodo.5914708)^74^.
