# Supplementary material for: Measuring protected-area effectiveness using vertebrate distributions from leech iDNA
Source: Nat Commun. 2022 Mar 23;13:1555. doi: 10.1038/s41467-022-28778-8 (PMC8943135; doi:10.1038/s41467-022-28778-8)
Supplement: Supplementary file 1 — Supplementary Information [file 41467_2022_28778_MOESM1_ESM.pdf]

Measuring Protected-Area Effectiveness using Vertebrate Distributions from Leech iDNA

Yinqiu Ji*, Christopher CM Baker*, Viorel D Popescu, Jiaxin Wang, Chunying Wu, Zhengyang Wang, Yuanheng Li, Lin Wang, Chaolang Hua, Zhongxing Yang, Chunyan Yang, Charles CY Xu, Alex Diana, Qingzhong Wen, Naomi E Pierce, and Douglas W Yu

*These authors contributed equally to this work.

Contents

1 Supplementary Methods: Laboratory Processing **1**

2 Supplementary Methods: Bioinformatics Pipeline **2**

3 Supplementary Methods: Site-occupancy Modelling **4**

4 Supplementary Figures **9**

5 Supplementary Note: Chinese Language Main Text **20**

1 Supplementary Methods: Laboratory Processing

DNA extraction. We extracted DNA from each replicate sample following the protocol in [1]. Leeches were transferred to a new tube to remove the preservative, soaked in a volume of digestion buffer (10 mM Tris-HCl, 10 mM NaCl, 2% SDS, 5 mM $CaCl_2$, 2.5 mM EDTA, 40 mM dithiothreitol, and 0.2 mg/mL Proteinase K) equal to 5 times the volume of each sample's leeches, and incubated at 55 °C (rotating) until all the leeches were dissolved. Following this incubation, we aliquoted 0.6 mL of digestion buffer from each sample for purification with the QIAquick PCR purification kit (Qiagen, Hilden, Germany). To detect any DNA cross-contamination, negative controls were created in both steps, digestion and purification.

PCR amplification. We PCR-amplified two mitochondrial markers: one from the 16S rRNA gene (*MT-RNR2*; primers *16Smam1*: 5'-CGGTTGGGGTGACCTCGGA-3' and *16Smam2*: 5'-GCTGTTATCCCTAGGGTAACT-3' [2]), and the other from the 12S rRNA gene (*MT-RNR1*; primers forward: 5'-ACTGGGATTAGATACCCC-3' and reverse: 5'-YRGAACAGGCTCCTCTAG-3' modified from [3]). Target fragments were 81 to 117 bp and 82 to 150 bp respectively, excluding primers. Hereafter, and throughout the manuscript, we refer to these two markers as LSU (16S) and SSU (12S), referring to the ribosomal large subunit and small subunit that these genes code for. (We do this in part to avoid confusion with the widely used bacterial 16S gene, which is homologous to our 12S marker, rather than our 16S.) The LSU primers are designed to target mammals, and the SSU primers to amplify all vertebrates. A third primer pair targeting the standard cytochrome *c* oxidase I marker [4] was tested but not adopted

in this study as it co-amplified leech DNA and consequently returned few vertebrate reads. We also tried using human blocking primers, to help avoid wasting read depth on human amplicons. However, in our initial trials, we found that over 70% of our samples failed in PCR, and even successful amplifications had low yields. We did not pursue this approach further, and instead chose to compensate for the presence of human reads by increasing sequencing depth.

Primers were ordered with sample-identifying tag sequences. We used 8-bp tags with a minimum difference of 3 nucleotides. The file 8bp_Tags_leeches.txt lists all tag sequences used in this study and is available at `https://github.com/jiyinqiu/ailaoshan_leeches_method_code`. To identify (and remove) 'tag jumping' errors [5], we used a 'twin-tagging strategy,' meaning that both forward and reverse primers used the same tag sequence for a sample (e.g. F1/R1, F2/R2, F3/R3). Thus, if a library contained tag combinations F1/R1, F2/R2, and F3/R3, an F1 tag-jump would produce F1/R2 or F1/R3, which could be detected and removed, since these combinations were not used in this library. We used the DAMe protocol [6] to remove these tag-jumped Illumina reads and to identify and remove reads containing PCR and/or sequencing errors. The DAMe protocol PCR-amplifies each sample three times per marker, each time with a different twin-tag pair, which allows the PCRs to be individually identified after sequencing. Reads containing errors are more likely to show up in only one PCR and at low copy numbers, which allows them to be filtered out bioinformatically (see below). Different libraries sent for sequencing at the same time (see below for details of library construction), and thus potentially sequenced in the same lane, used different sets of tag pairs so that we could also identify and remove any mis-assigned samples due to index hopping [7].

We used the same PCR conditions for both markers. The 20 μL PCR reactions consisted of 1X buffer, 1.5 mM $MgCl_2$, 0.2 mM dNTPs, 0.2 μM per primer (synthesized by Invitrogen, Shanghai, China), 5% DMSO (Amresco, Solon, Ohio, USA), 0.6 U ExTaq HotStart DNA polymerase (TAKARA Biosystems, Dalian, China), and 1 μL of template DNA, with a thermal cycling profile of 95 °C for 5 min, then 40 cycles of 95 °C for 30 s, 59 °C for 30 s, and 72 °C for 45 s, with a final extension time of 7 min at 72 °C. PCR products were visualized on 2.5% agarose gels, and samples that failed to produce a band at the expected size were reattempted at least three times. The successfully amplified samples were quantified using the Quant-iT PicoGreen dsDNA Assay kit (Invitrogen, New York, USA), and equal masses pooled into a total of 13 LSU and 14 SSU libraries. The number of twin-tagged replicates pooled into each library ranged from 100 to 245. For most samples, all PCR replicates were pooled into the same libraries. The main exceptions were reattempted PCRs, since some reattempts happened after the successful PCR replicates had been sent out for sequencing. LSU and SSU amplicons were never pooled into the same library. Libraries were purified with QIAquick gel extraction kit (QIAGEN, Hilden, Germany), and sent to Novogene (Beijing, China) for library construction using the PCR-free NEBNext Ultra II DNA Library Prep Kit (Ipswich, MA, USA), and 150 bp paired-end sequencing on an Illumina Hiseq X.

2 Supplementary Methods: Bioinformatics Pipeline

Preprocessing. Sequencing of the 27 libraries yielded a total of 1.354×10^9 paired-end reads. We used AdapterRemoval v2.1.7 [8] to remove adapter sequences from reads and Sickle v1.33 [9] to trim reads of low quality nucleotides. We then used BFC v181 (parameters: `-s 3g -k 25`) [10] to de-noise the reads, and we merged the read pairs with Pandaseq v2.11 [11]. Except for BFC, we used default parameters.

Demultiplexing and DAMe quality filtering. To filter out tag-jumping events and to remove artifactual reads arising from PCR or sequencing errors, we used the DAMe pipeline [6]. DAMe's `sort.py` function was used to remove reads with unused tag combinations, and the `filter.py` function was used to keep only the haplotypes that appeared in ≥2 PCRs, with ≥9 (LSU) or ≥20 (SSU) copies per PCR, using the logic that sequences which appear in multiple, independent PCRs and in multiple copies per PCR are more likely to be true sequences (`filter.py` parameters for 12S: `-x 3 -y 2 -p 14 -t 20 -l 81`; for 16S: `-x 3 -y 2 -p 13 -t 9 -l 82`). Filtering parameters were chosen after inspection of the control samples. After DAMe filtering, each PCR replicate yielded about 105,000 sequences.

De novo chimera removal. DAMe filtering also removes the chimeric sequences that can result from incomplete PCR extension, but we also used the *de novo* chimera detection function `uchime_denovo` in VSEARCH v2.9.0 [12] to remove any remaining chimeras after dereplicating with the `derep_fulllength` function.

Clustering into preliminary operational taxonomic units. We used Swarm v2.0 [13] to cluster the filtered sequences into preliminary OTUs ('pre-OTUs') and then used the R package `LULU` v0.1.0 [14] to merge Swarm pre-OTUs that shared high similarity and distribution across samples (i.e. over-split OTUs) and output a representative sequence for each pre-OTU. For both, we used default values.

Assigning taxonomy to preliminary operational taxonomic units. One of the more crucial steps in the iDNA bioinformatic pipeline is taxonomic assignment. With vertebrates, exact species identity can have important management consequences because some species, but not their close relatives, are given high conservation value [15]. Existing taxonomic assignment programs are typically biased toward assigning sequences to species that happen to be in a reference database, even though we know that some of our leech-derived sequences are likely from known species that have never been sequenced, or more rarely, that are undescribed. We thus used PROTAX for taxonomic assignment of the pre-OTU sequences [16, 17]. PROTAX provides an unbiased, estimated probability of assignment at each rank, where unbiased means, for example, that 70% of all assignments given a 70% probability of accuracy are indeed correct. Thus, a PROTAX assignment of a pre-OTU to Carnivora (probability = 0.999)/Canidae (0.996)/*Nyctereutes* (0.821)/*Nyctereutes procyonoides* (0.557) means that this pre-OTU is very likely to be in the genus *Nyctereutes*, but there is a $(1 - 0.557) = 44.3\%$ probability that the species is not *N. procyonoides*. PROTAX can also estimate the probability that a pre-OTU sequence is 'unknown,' i.e. not in the reference database. Thus, PROTAX helps prevent mistaken assignments of sequences to species, potentially avoiding wasted management effort directed towards species that are not actually present.

We refer the reader to Somervuo *et al.* [16, 17] for in-depth discussions of PROTAX and to Axtner *et al.* [15] for details of the bioinformatic pipeline used to create the LSU and SSU reference databases and to train and assess the PROTAX models. We built the reference databases starting from the Midori Unique_20180221_lrRNA and Unique_20180221_srRNA databases [18], supplemented with mitogenomes from [19]. We used the R package `taxize` [20] to build a taxonomy database of all Tetrapoda and to harmonize species names between the Tetrapoda taxonomies and the sequences in the Midori + Salleh reference database, and we used SATIVA [21] to identify reference sequences mislabelled at family level and above, which we removed. With the curated reference database, we then trained PROTAX models for both LSU and SSU, setting a 90% prior probability for the set of Tetrapoda species known from Ailaoshan, thereby reducing false-positive assignments [22]. Raw similarities between each query and all reference sequences were calculated with LAST v.982 [23], after which the trained PRO-

TAX models were used to assign probabilities of assignment for pre-OTUs at class, order, family, genus, and species ranks. The bioinformatic scripts, reference datasets, trained models, and bias-accuracy plots are available for download from GitHub [24].

Using pairwise correlations between LSU and SSU OTUs to reconcile taxonomies. Different marker genes have different levels of taxonomic coverage and discrimination power [16, 17], and as a result, the same species can be assigned to different taxonomies by SSU and LSU. For instance, as described above, the SSU dataset confidently detected *Nyctereutes procyonoides*, but the LSU dataset did not, although it did assign one OTU to Carnivora (probability = 0.999)/Canidae (0.999)/*Canis* (0.475)/*Vulpes*, unknown species (0.231). Given the confident assignment to Canidae, this LSU OTU might also have derived from *Nyctereutes*. To combine taxonomic information across the two markers, we therefore calculated pairwise correlations of SSU and LSU pre-OTUs across the 619 replicates for which both markers had amplified and visualized the correlations as a network (Supplementary Fig. 2). If an SSU and an LSU pre-OTU occur in the same subset of replicates and are assigned the same higher-level taxonomies, the two pre-OTUs are likely to have been amplified from the same set of leeches feeding on the same species. We manually inspected the network diagram and assigned such correlated pre-OTU pairs the same taxonomy.

Final operational taxonomic units and dataset filtering. After using PROTAX and then searching for network correlations to assign taxonomies to pre-OTUs, we verified that the positive and negative control samples were free of any substantive contaminants before removing them from the dataset, along with one sample that had neither ranger nor patrol area information. We eliminated any pre-OTUs to which we were unable to assign a taxonomy; these pre-OTUs only accounted for 0.9% and 0.2% of reads in the LSU and SSU datasets respectively, and most likely represent erroneous sequences rather than novel taxa. Within the LSU and SSU datasets, we merged pre-OTUs that had been assigned the same taxonomies, thus generating a final set of OTUs for each dataset. Finally, we removed the OTU identified as *Homo sapiens* from both datasets prior to analysis. As expected, since the leeches were collected with bare hands and might have in some cases been feeding on the rangers themselves, human DNA was obtained from the majority of samples in both datasets.

Our final OTUs are intended to be interpreted as species-level groups, even though some could not be assigned taxonomic names to species level. We therefore refer to our final OTUs as species throughout the main text. After excluding humans, the final LSU and SSU datasets comprised 18,502,593 and 84,951,011 reads respectively. These reads were assigned to a total of 72 species across 740 replicates and 127 patrol areas in the SSU dataset, and 59 species across 653 replicates and 126 patrol areas in the LSU dataset. We attached IUCN data for individual species by using the R package `rredlist` v0.6.0 [25] to search for scientific names assigned by PROTAX (or synonyms where we were aware of nomenclature changes). For mammals, we used the PanTHERIA database [26] to obtain data on adult body mass for each species; where species-level information was not available, we used the median adult body mass from the database for the lowest taxonomic group possible.

3 Supplementary Methods: Site-occupancy Modelling

Overview. We used hierarchical multispecies site-occupancy models to analyze our data, using parameter-expanded data augmentation [27, 28], an extension of the single-season occupancy model in [29]. We estimated separate models for the LSU and SSU

data.

These models assume that the $n_{\text{LSU}} = 59$ and $n_{\text{SSU}} = 72$ species observed in each dataset are, respectively, subsets of larger communities of size N_{LSU} and N_{SSU} species that are present in the vicinity of Ailaoshan and vulnerable to capture (e.g. fed on by leeches and amplified by the LSU and SSU primers). Although N_{LSU} and N_{SSU} are unknown, these communities can be modelled by embedding them in a larger 'supercommunity' of fixed size M. We wanted to choose a value of M that was as small as possible to minimize computational effort, but large enough that it did not materially constrain model estimates. We therefore estimated models with values of M ranging from 100 to 474 (the latter being the total species richness for mammals, birds, non-avian reptiles and amphibians in the 1984-85 survey of Ailaoshan [30], which might be regarded as a reasonable upper bound on true species richness). Estimates of N_{LSU} and N_{SSU} were similar for $M \geq 150$, and we chose to set $M = 200$ for our final models.

For each species in the supercommunity, our models explicitly capture (i) a 'community process' governing whether the species is in the Ailaoshan community or not; (ii) an 'ecological process' governing the presence or absence of the species in each patrol area, given that it is in the community; and (iii) an 'observation process' governing whether we detect the species' DNA in each of our replicate samples, given that it is present in the patrol area. The community-, ecological- and observation processes for individual species are linked by imposing community-level parameters and priors as described here.

In addition to the detailed model description provided here, the data and code to produce our model results are available on GitHub at [31].

Community process. Each species i was assumed to be either a member of the Ailaoshan community or not. We denote this unobserved state with w_i, which was assumed to be a Bernoulli random variable governed by the community membership parameter Ω_{g_i}, i.e. the probability that species i was in the Ailaoshan community:

$$w_i \sim \text{Bernoulli}(\Omega_{g_i}). \tag{1}$$

For the community process, we separated the species into two natural groupings – homeothermic mammals and birds, and poikilothermic amphibians and squamates – and allowed them to have different probabilities of being in the Ailaoshan community. This is denoted by the subscript on the Ω_{g_i} parameter, in which g_i represents which of these two groupings species i belongs to. This approach reflected our expectation that these groupings would differ systematically in their community probabilities, and we employed the same grouping for parameters governing the ecological and detection processes (see *Community model* below for further discussion). We assigned unobserved species to these two groups such that the assumed size of each group in the supercommunity varied linearly with M between the observed values from the LSU dataset in our study (i.e. 36 mammals + birds, and 23 amphibians + squamates) when $M = n_{LSU} = 59$, and the observed values in the 1984-85 survey of Ailaoshan (i.e. 409 mammals + birds, and 65 amphibians + squamates [30]) when $M = 474$, which was the total observed richness in the 1984-85 survey. (We used the LSU data to anchor the group sizes for both datasets in our analysis so that the assumed supercommunity for any M was the same for both datasets.)

Ecological process. Each species i was assumed to be either present or absent in each patrol area j. We used z_{ij} to denote this unobserved ecological state, with values of 1 and 0 corresponding to presence and absence respectively. We assumed that the z_{ij} are constant across all replicates taken from patrol area j – sometimes referred to as the 'closure' assumption – consistent with all the leech samples for any patrol area

being collected at essentially the same point in time. Any species present were assumed to be members of the Ailaoshan community (i.e. $w_i = 1$), so we modelled z_{ij} as a Bernoulli random variable governed by both w_i and an occupancy parameter ψ_{ij}, i.e. the probability that a species i in the community was present in patrol area j:

$$z_{ij}|w_i \sim \text{Bernoulli}(w_i\psi_{ij}). \tag{2}$$

We allowed the occupancy probability ψ_{ij} to vary among species as well as among patrol areas, to capture e.g. preferences of different species for particular habitat types. In particular, we modelled ψ_{ij} as a function of environmental covariates that varied over the patrol areas, scaled by species-specific coefficients:

$$\text{logit}(\psi_{ij}) = \beta_{0i} + \beta_{1i}\text{elevation}_j + \beta_{2i}\text{TPI}_j + \beta_{3i}\text{road}_j + \beta_{4i}\text{stream}_j + \beta_{5i}\text{reserve}_j \tag{3}$$

where elevation_j, TPI_j, road_j, stream_j and reserve_j are, respectively, the median values of elevation, topographic position index, distance to nearest road, distance to nearest stream, and the distance from centroid to nature reserve boundary for patrol area j, and the $\beta_{\bullet i}$ are the usual logit-scale slope coefficients. All occupancy covariates were normalized to a mean of 0 and a standard deviation of 1 prior to modelling.

We began by estimating the full model in (3), but ultimately reduced the set of occupancy covariates to elevation + reserve for the LSU dataset, and elevation for the SSU dataset. See *Model selection* below for details.

Observation process. Although we cannot directly observe the true ecological state z_{ij}, we do know whether we detected DNA from species i in each replicate k from patrol area j. But this is an imperfect proxy for the true ecological state. For replicate k from patrol area j, we assumed that we detected DNA from species i with probability p_{ijk} when i was truly present in patrol area j, and with probability 0 when i was absent:

$$y_{ijk}|z_{ij} \sim \text{Bernoulli}(z_{ij}.p_{ijk}), \tag{4}$$

where the y_{ijk} are the observed data (i.e. detection or non-detection of species i's DNA in each replicate). Our model therefore assumes that false positives do not occur, i.e. that we never falsely detect species i's DNA through lab contamination or through incorrectly assigned sequence reads. On the other hand, since p_{ijk} may be less than one, it allows for the possibility of false negatives, i.e. that we failed to detect species i's DNA when species i was actually present. Although false positives probably do occur, we focused mainly on lab procedures (e.g. use of negative controls) and the taxonomic assignment pipeline (e.g. use of DADA2 [32] to filter out OTUs not observed in ≥ 2 technical replicates) to address these, and we expect false negatives to far outstrip false positives in our final datasets.

We modelled the conditional detection probability p_{ijk} as a function of the conditional detection probability for species i per 100 leeches, r_i, and the number of leeches in the replicate, leeches_{jk}:

$$p_{ijk} = 1 - (1 - r_i)^{\text{leeches}_{jk}/100} \tag{5}$$
$$\text{logit}(r_i) = \gamma_{0i} \tag{6}$$

We allowed r_i (and its logit-scale equivalent, γ_{0i}) to vary among species, to capture e.g. variation in leech feeding preferences for different taxa. We used $\text{leeches}_{jk}/100$ rather than leeches_{jk} to avoid computational problems arising from rounding that prevented fitting the model.

Community model. Equations (1) through (6) define a site-occupancy model for each species i. We united these species-specific models with community models for both ecological and observation processes, by assuming that the species-level β and γ parameters come from community-level distributions:

$$\beta_{mi} \sim \mathrm{N}(\mu_{\beta_m}, \sigma^2_{\beta_m}) \qquad m = 1, 2, 3, 4, 5 \tag{7}$$

$$(\beta_{0i}, \gamma_{0i}) \sim \mathrm{MVN}([\mu_{\beta_0 g_i}, \mu_{\gamma_0 g_i}], \begin{bmatrix} \sigma^2_{\beta_0 g_i} & \rho\sigma_{\beta_0 g_i}\sigma_{\gamma_0 g_i} \\ \rho\sigma_{\beta_0 g_i}\sigma_{\gamma_0 g_i} & \sigma^2_{\gamma_0 g_i} \end{bmatrix}) \tag{8}$$

where N( ) and MVN( ) denote normal and multivariate normal distributions respectively. These distributions were characterized by community-level hyperparameters $\mu_\bullet$ and $\sigma_\bullet$, with separate distributions for each parameter as denoted by the first subscript. We used a multivariate normal prior for $(\beta_{0i}, \gamma_{0i})$ to allow non-zero covariance between species' occupancy and detection probabilities, as we might expect if, for example, variation in abundance affects both probabilities [27].

These community models allow rare species effectively to borrow information from more common ones, producing a better overall ensemble of parameter estimates [27, 33, 34]. As for the community process described above, we separated the species into two groups – homeothermic mammals and birds, and poikilothermic amphibians and squamates – and allowed them to have different community distributions. This is denoted by the subscripts on the $\mu_\bullet$ and $\sigma_\bullet$ community hyperparameters for the occupancy and detection intercepts, in which g_i represents which of these two groupings species i belongs to. This approach reflected our expectation that these groupings would differ systematically in occupancy probabilities (e.g. due to different habitat preferences) and in detection probabilities (e.g. due to different encounter rates with leeches, or leech feeding preferences).

Missing data. Incompletely labelled data points (i.e. sequence data without records of which patrol areas they came from) were retained in the model by including these data points without accompanying environmental covariates. Since the identity of the collecting ranger was known and could be used to identify replicates that came from the same unknown location, this allowed these data to contribute to both detection and occupancy estimates. At the same time, we generated occupancy estimates for patrol areas without accompanying data by augmenting the data matrix with rows of missing values and including their environmental covariates.

Choice of priors. For the Ω_{g_i} parameters in (1), our initial exploration with broad priors (e.g. uniform [0,1]) and different values of M revealed that N_{LSU} and N_{SSU} were likely to be in the order of 100 to 200 species. We thereafter focused on models estimated with $M = 100$, 150 and 200. To facilitate comparisons between these different values of M, we switched from using uniform [0,1] priors on Ω_{g_i} to using a beta(5,b) distribution where $b = 0.6$ when $M = 100$, $b = 3.3$ when $M = 150$, and $b = 6.1$ when $M = 200$. This choice of distributions had the effect of keeping the expected species richness at around 90 species for all three values of M without constraining species richness unduly.

For the μ and σ hyperparameters in (7) and (8), our intention was to use priors that would be uninformative on the probability scale. We chose the t-distribution with $\sigma = 1.566267$ and $\nu = 7.763179$ proposed in [28] for each of the $\mu_{\beta_0} g_i$ and μ_{β_b} $(b = 1, ..., 5)$ hyperparameters; the half-Cauchy $\nu = 1$ distribution proposed by Gelman [35] for each of the $\sigma_{\beta_0 g_i}$, $\sigma_{\gamma_0 g_i}$ and σ_{β_b} $(b = 1, ..., 5)$ hyperparameters; and a uniform [-1,1] distribution for ρ.

Model selection. Our final models, as reported in the main text of this paper, used a reduced set of occupancy covariates: elevation + reserve for the LSU dataset, and ele-

vation for the SSU dataset. To arrive at these model selections, we began by estimating the full model in (3), and examined the posterior distributions for the slope parameters. We retained in our final model those covariates for which the 95% Bayesian confidence interval excluded zero.

Model estimation. We estimated our models using a Bayesian framework with JAGS v4.3.0 [36] in R v3.5.1 [37] via `rjags` v4.8 [38] and `jagsUI` v.1.5.1 [39]. We ran 5 Markov chains of 100,000 generations, including burn-in of 50,000. We retained all rounds (i.e. without thinning) for the posterior sample, except for where we needed to save the z matrix for beta diversity or cluster occupancy calculations; memory limitations prevented us from retaining all posterior samples for the z matrix, and we thinned tenfold in order to make these calculations feasible. We assessed convergence and MCMC mixing by inspecting trace plots, and confirmed convergence by ensuring that the $\hat{R}$ statistics were close to 1 [40, 41].

4 Supplementary Figures

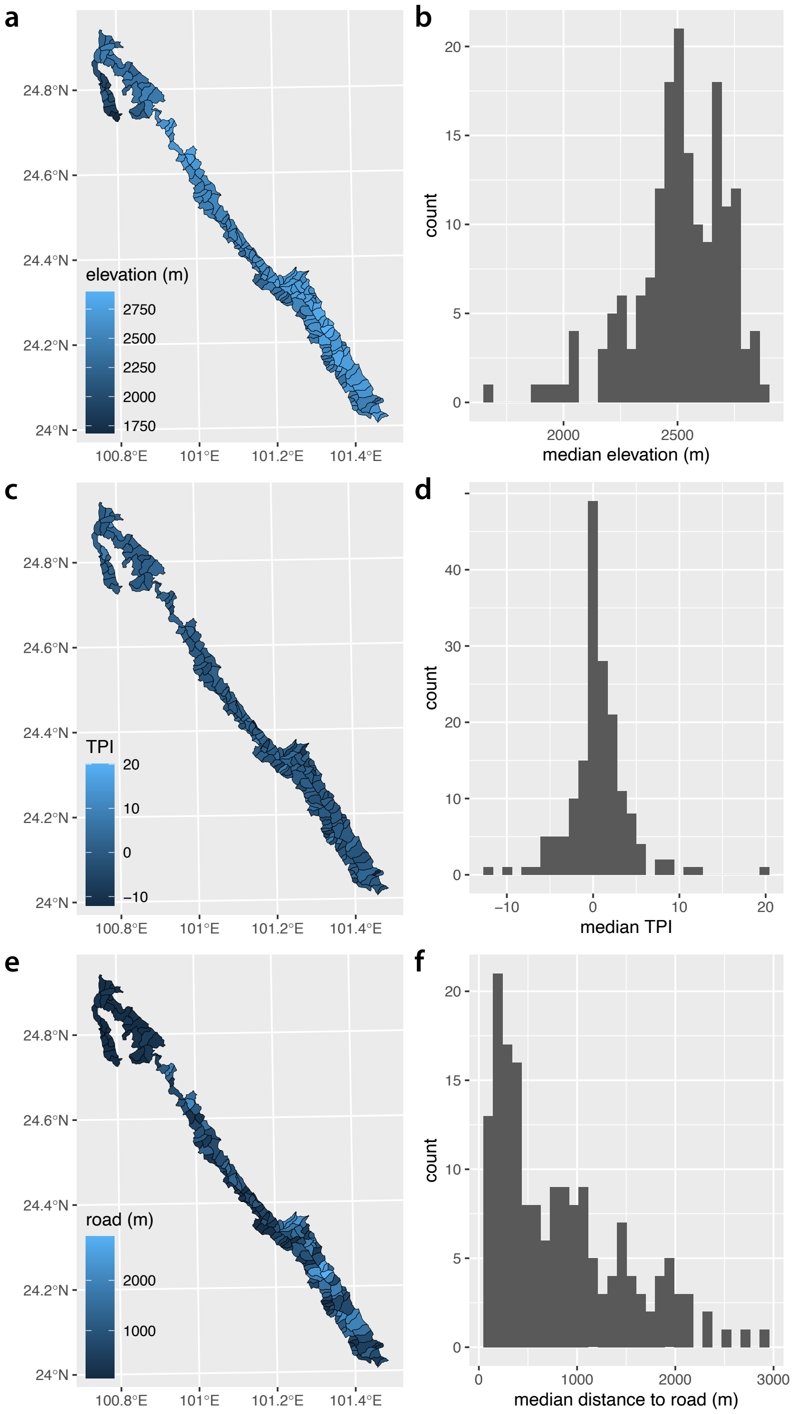

Supplementary Figure 1: Environmental covariates. Maps and histograms for environmental covariates used in occupancy modelling. **(a,b)** Median elevation. **(c,d)** Median topographic position index (TPI). **(e,f)** Median distance to nearest road.

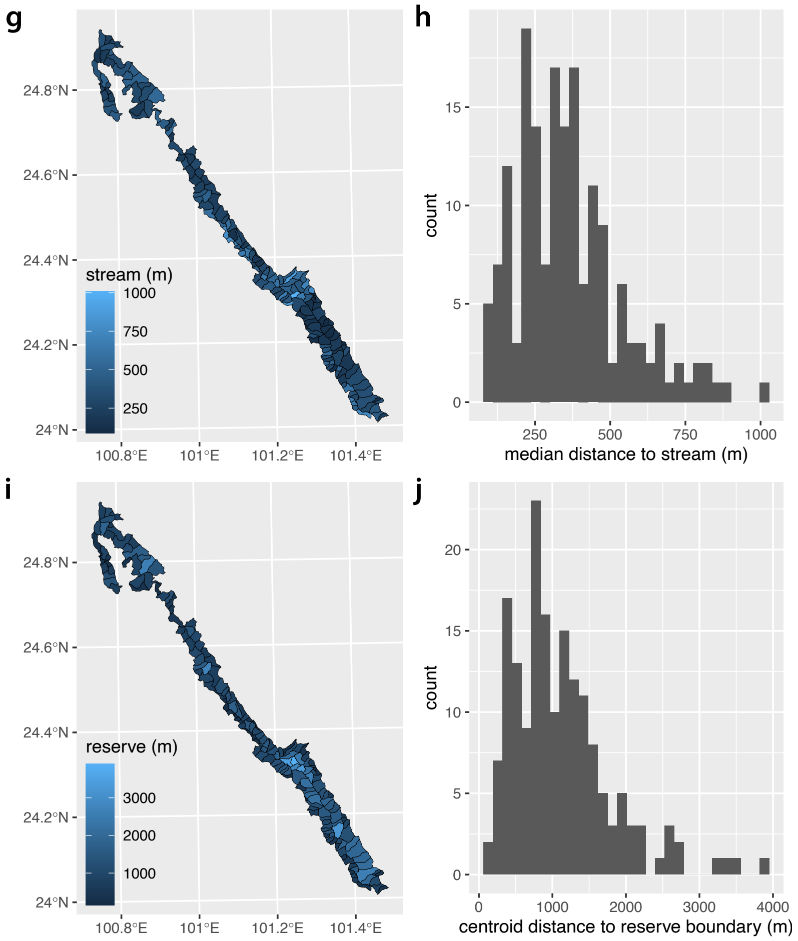

Supplementary Figure 1: Environmental covariates (continued). Maps and histograms for environmental covariates used in occupancy modelling. **(g,h)** Median distance to nearest stream. **(i,j)** Distance from patrol area centroid to nearest reserve edge.

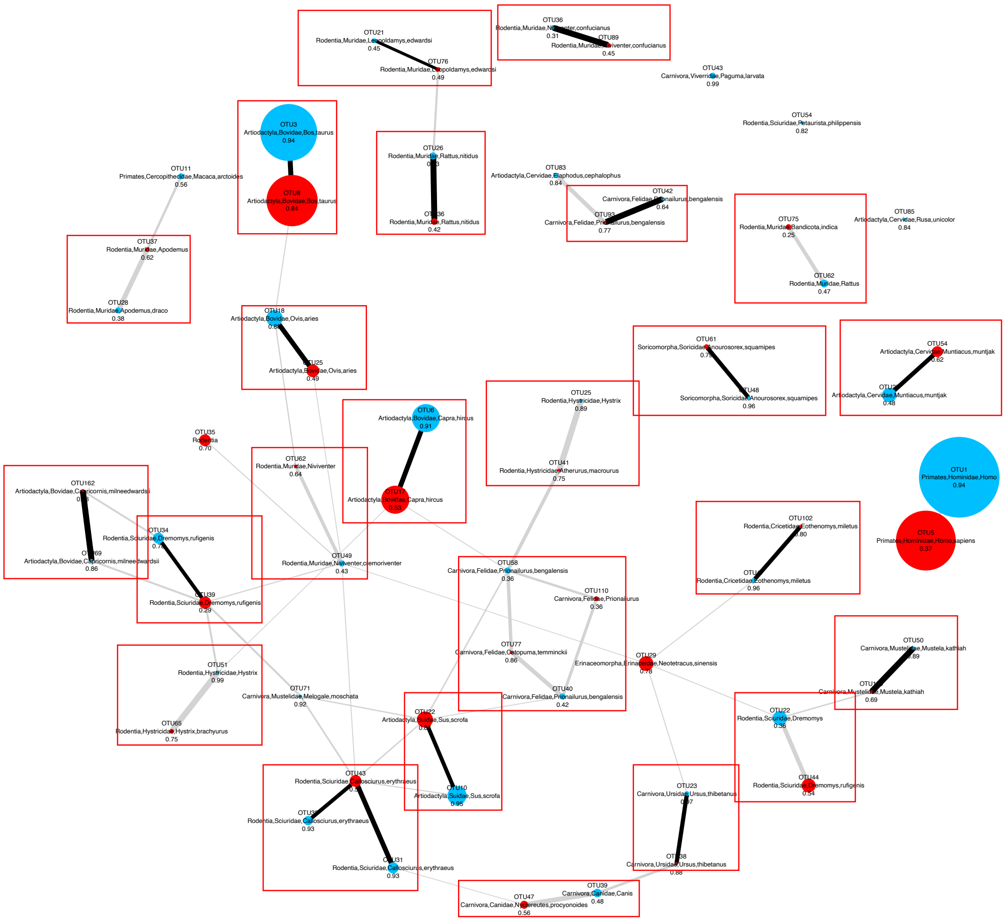

Supplementary Figure 2: Pre-OTU correlations between datasets. Bipartite network visualization of pairwise Spearman correlations between mammal LSU and SSU pre-OTUs across lab replicates. Blue and red nodes represent pre-OTUs from the LSU and SSU datasets respectively. The size of each node is proportional to the square-root transformed occupancy of the pre-OTU calculated across lab replicates (i.e. the fraction of replicates in which the pre-OTU was detected). Each node is labelled with the lowest taxonomic assignment that was not missing or unknown, as well as the PROTAX probability for that assignment. For every pair of LSU and SSU pre-OTUs, we calculated the Spearman correlation of read counts across lab replicates. We discarded any correlations that were < 0.1, or that were not significant at $\alpha = 0.5$ after false discovery rate correction. We drew a bipartite graph using the package `igraph` [42] with the remaining correlations as edge weights connecting nodes representing the pre-OTUs. Thicker edges thus indicate higher correlation coefficients. Edges are shown in black where they join nodes with the same lowest taxonomic assignment, and are otherwise shown in grey. Red boxes show manually assigned groupings of pre-OTUs that were deemed to be the same taxon. For example, at the bottom of the figure, pre-OTU38 (SSU) and pre-OTU23 (LSU) were both assigned to the Asiatic black bear, *Ursus thibetanus*, and the thick line indicates that these OTUs were found in (nearly) the same subset of replicates, as expected if the two OTUs were amplified from the same bloodmeals and thus from the same individual mammals. Also at the bottom of the figure, pre-OTU47 (SSU) was assigned to Canidae, *Nyctereutes procyonoides*, but pre-OTU39 (LSU) was assigned to Canidae, *Canis*. Given that these OTUs were also found in nearly the same subset of replicates, we conclude that pre-OTU39 is also *N. procyonoides*.

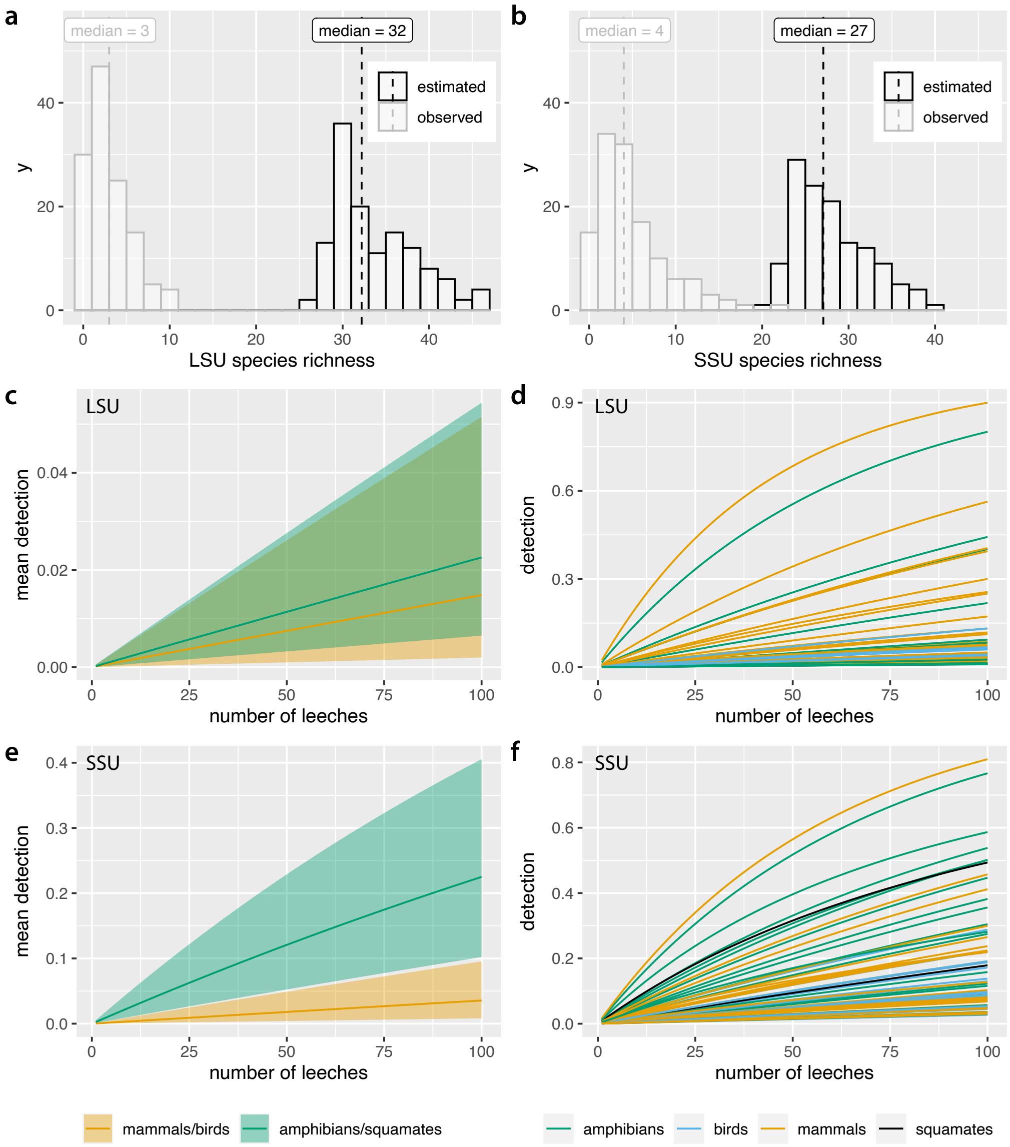

Supplementary Figure 3: Species richness and detection estimates. Histograms of observed and estimated species richness per patrol area in **(a)** the LSU and **(b)** the SSU datasets respectively. Dashed lines in panels (a) and (b) show median values. **(c)** Community mean detection estimates and **(d)** detection estimates for each species as a function of number of leeches per replicate in the LSU dataset. **(e)** Community mean detection estimates and **(f)** detection estimates for each species as a function of number of leeches per replicate in the SSU dataset. Lines in panels (c) through (f) show posterior means. Shaded areas in panels (c) and (e) show 95% Bayesian confidence intervals from models based on $n = 893$ replicate samples.

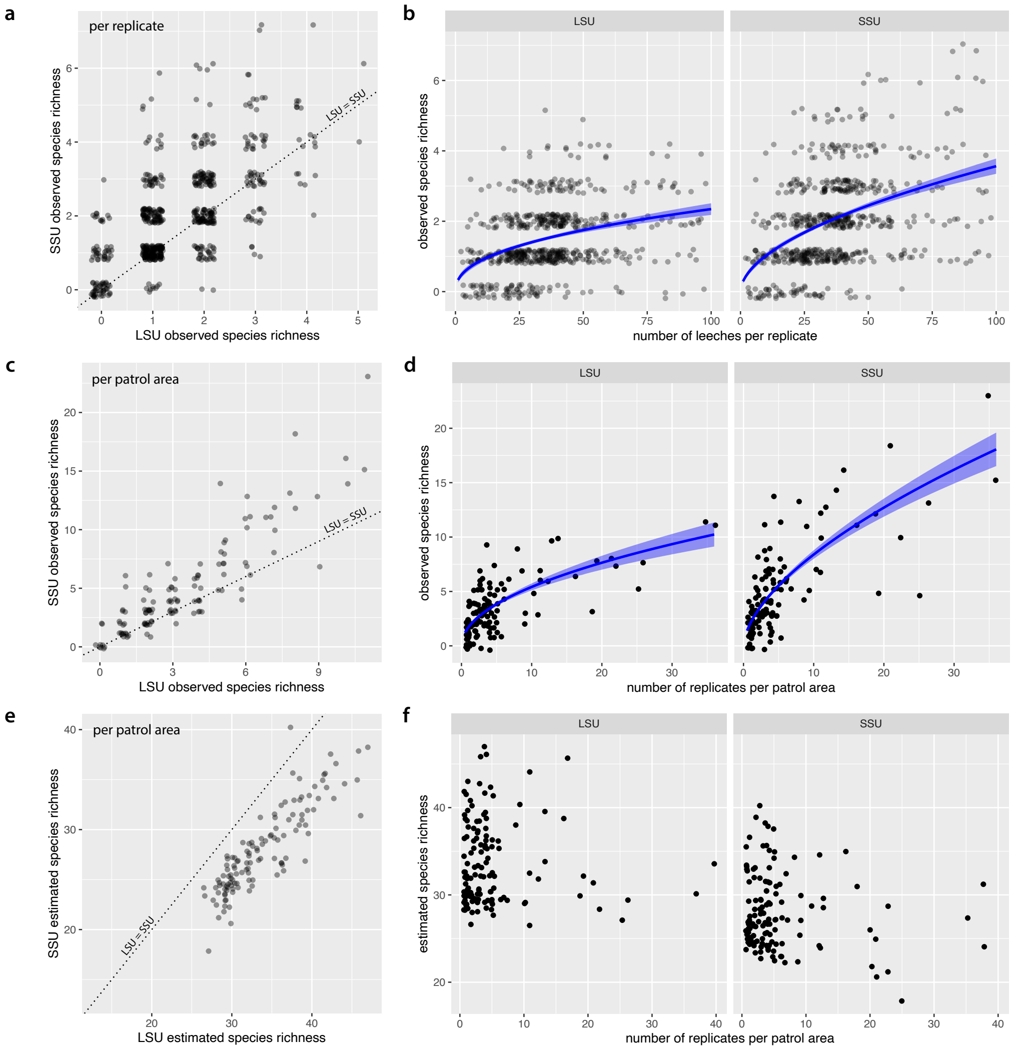

Supplementary Figure 4: Species richness correlations and relationships to sampling intensity. **(a)** Observed species richness per replicate was positively correlated between LSU and SSU datasets (Pearson's $r = 0.65$; $t_{616} = 21.2$, one-tailed $p < 0.001$). **(b)** More species were detected in replicates with more leeches. Blue curves show predicted values from Poisson GLMs of species richness against log-transformed number of leeches per replicate (LSU: slope $z-$ratio $= 6.9$, one-tailed $p < 0.001$; SSU: slope $z-$ratio $= 10.0$, one-tailed $p < 0.001$); shaded areas show $\pm$ standard error. **(c)** Observed species richness per patrol area was positively correlated between LSU and SSU datasets (Pearson's $r = 0.89$; $t_{120} = 20.8$, one-tailed $p < 0.001$). **(d)** More species were detected in patrol areas with more replicates. Blue curves show predicted values from Poisson GLMs of species richness against log-transformed number of replicates per patrol area (LSU: slope $z-$ratio $= 10.2$, one-tailed $p < 0.001$; SSU: $z-$ratio $= 14.9$, one-tailed $p < 0.001$); shaded areas show $\pm$ standard error. **(e)** Estimated species richness per patrol area was generally higher in the LSU dataset than the SSU dataset, and positively correlated between the two datasets (Pearson's $r = 0.87$; $t_{120} = 19.5$, one-tailed $p < 0.001$). **(f)** In contrast to observed species richness, estimated species richness did not increase with number of replicates per patrol area, as the occupancy model corrects for variation in sampling effort. Slope coefficients for least-squares regressions of estimated species richness against log-transformed number of replicates per patrol area were non-significant (LSU: $F_{1,124} = 0.006$, $p = 0.94$; SSU: $F_{1,125} = 1.5$, $p = 0.22$). Points in all plots are jittered to allow overlapping points to be visualized. p-values are not adjusted for multiple comparisons.

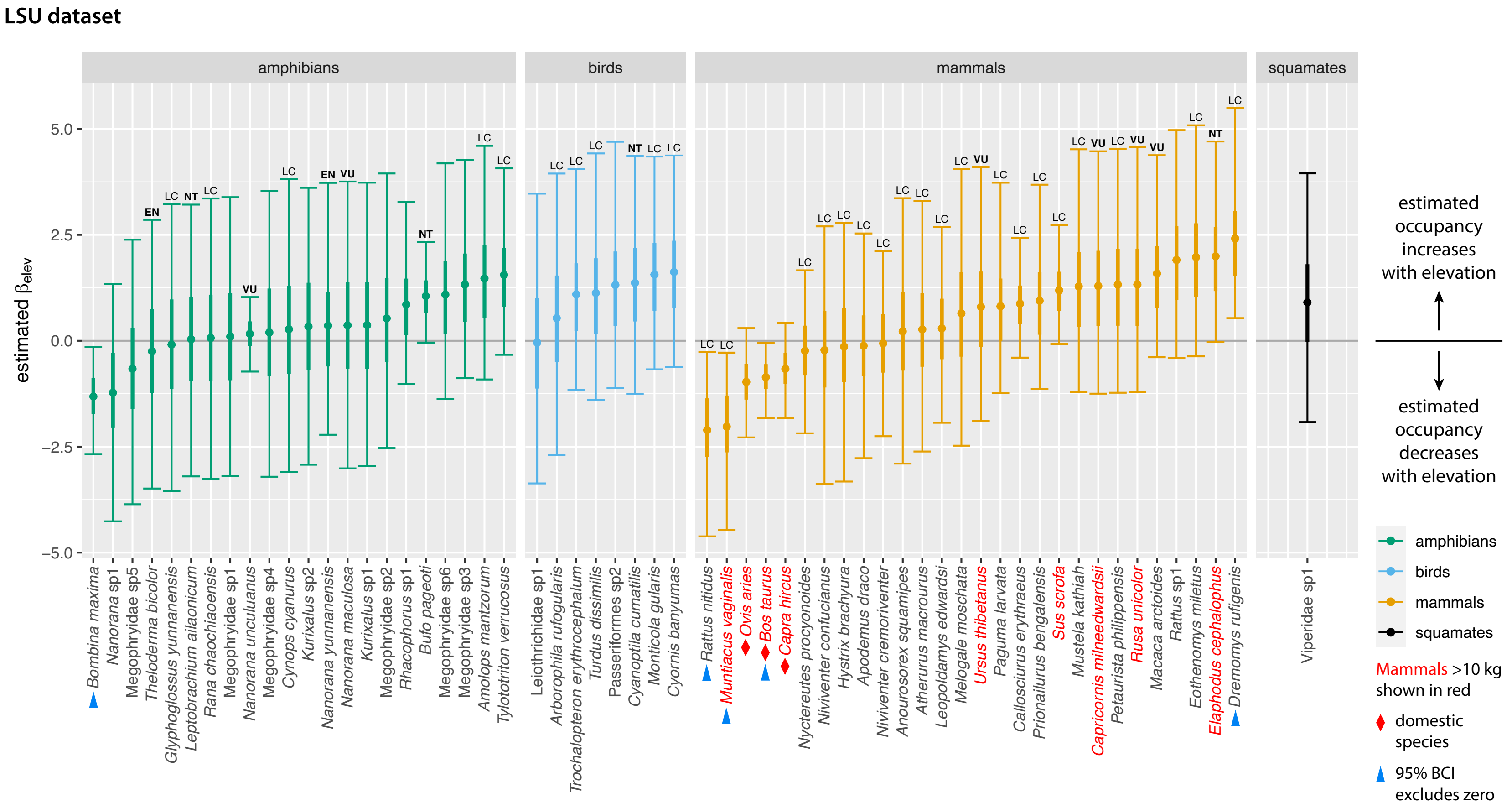

Supplementary Figure 5: LSU occupancy slope estimates (elevation) by species. Estimated occupancy slope coefficients on elevation from the LSU model. For each species, plot shows posterior mean (dot), interquartile range (thick line) and 95% Bayesian confidence interval (BCI; thin line with crossbars) from model based on $n = 893$ replicate samples. Slope coefficients are shown on the logit scale, so positive coefficients correspond to occupancy increasing with elevation. Within taxonomic groups, species are ordered by slope coefficient. Blue triangles mark species whose 95% BCI excludes zero. Annotations above bars denote IUCN categories: LC = Least Concern; NT = Near Threatened; VU = Vulnerable; EN = Endangered. Categories NT and above are shown in bold. Taxa without annotations have not been assigned a category by the IUCN. Species names for mammals over 10 kg adult body mass are shown in red. Domestic species are denoted with red diamonds.

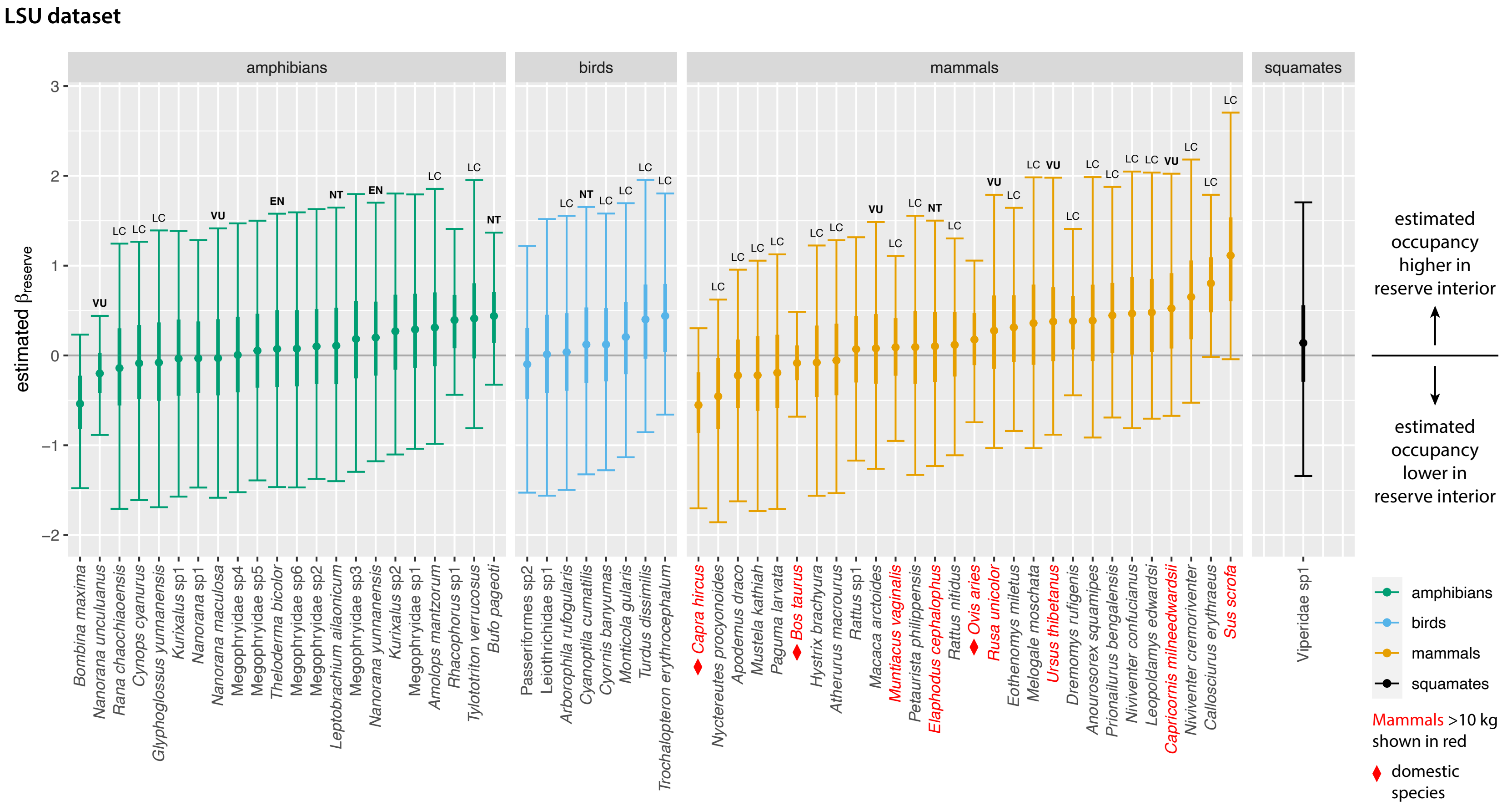

Supplementary Figure 6: LSU occupancy slope estimates (reserve edge distance) by species. Estimated occupancy slope coefficients on distance to reserve edge from the LSU model. For each species, plot shows posterior mean (dot), interquartile range (thick line) and 95% Bayesian confidence interval (BCI; thin line with crossbars) from model based on $n = 893$ replicate samples. Slope coefficients are shown on the logit scale, so positive coefficients correspond to occupancy increasing with distance to reserve edge. Within taxonomic groups, species are ordered by slope coefficient. No species had a 95% BCI that excluded zero. Annotations above bars denote IUCN categories: LC = Least Concern; NT = Near Threatened; VU = Vulnerable; EN = Endangered. Categories NT and above are shown in bold. Taxa without annotations have not been assigned a category by the IUCN. Species names for mammals over 10 kg adult body mass are shown in red. Domestic species are denoted with red diamonds.

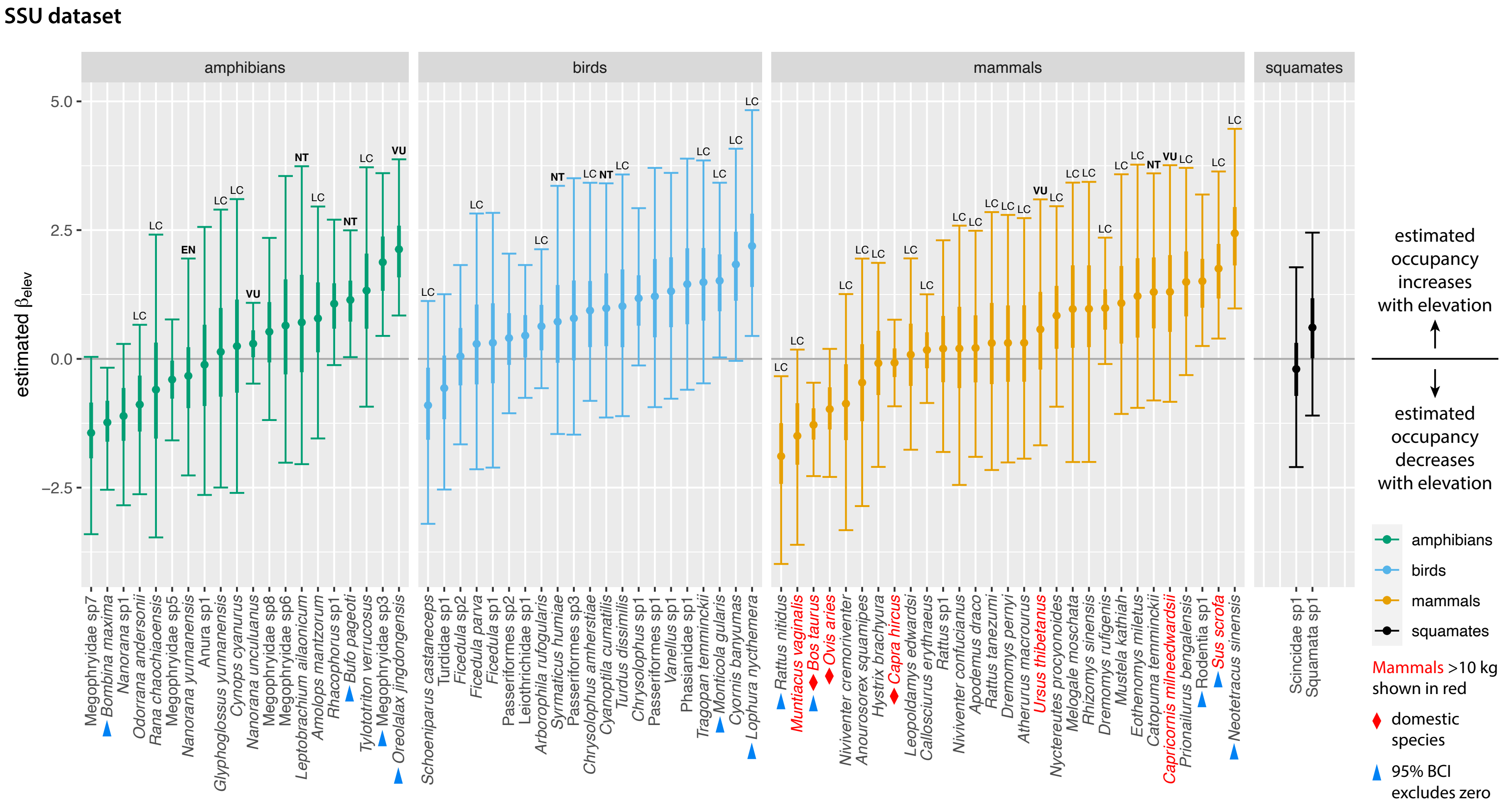

Supplementary Figure 7: SSU occupancy slope estimates (elevation) by species. Estimated occupancy slope coefficients on elevation from the SSU model. For each species, plot shows posterior mean (dot), interquartile range (thick line) and 95% Bayesian confidence interval (BCI; thin line with crossbars) from model based on $n = 893$ replicate samples. Slope coefficients are shown on the logit scale, so positive coefficients correspond to occupancy increasing with elevation. Within taxonomic groups, species are ordered by slope coefficient. Blue triangles mark species whose 95% BCI excludes zero. Annotations above bars denote IUCN categories: LC = Least Concern; NT = Near Threatened; VU = Vulnerable; EN = Endangered. Categories NT and above are shown in bold. Taxa without annotations have not been assigned a category by the IUCN. Species names for mammals over 10 kg adult body mass are shown in red. Domestic species are denoted with red diamonds.

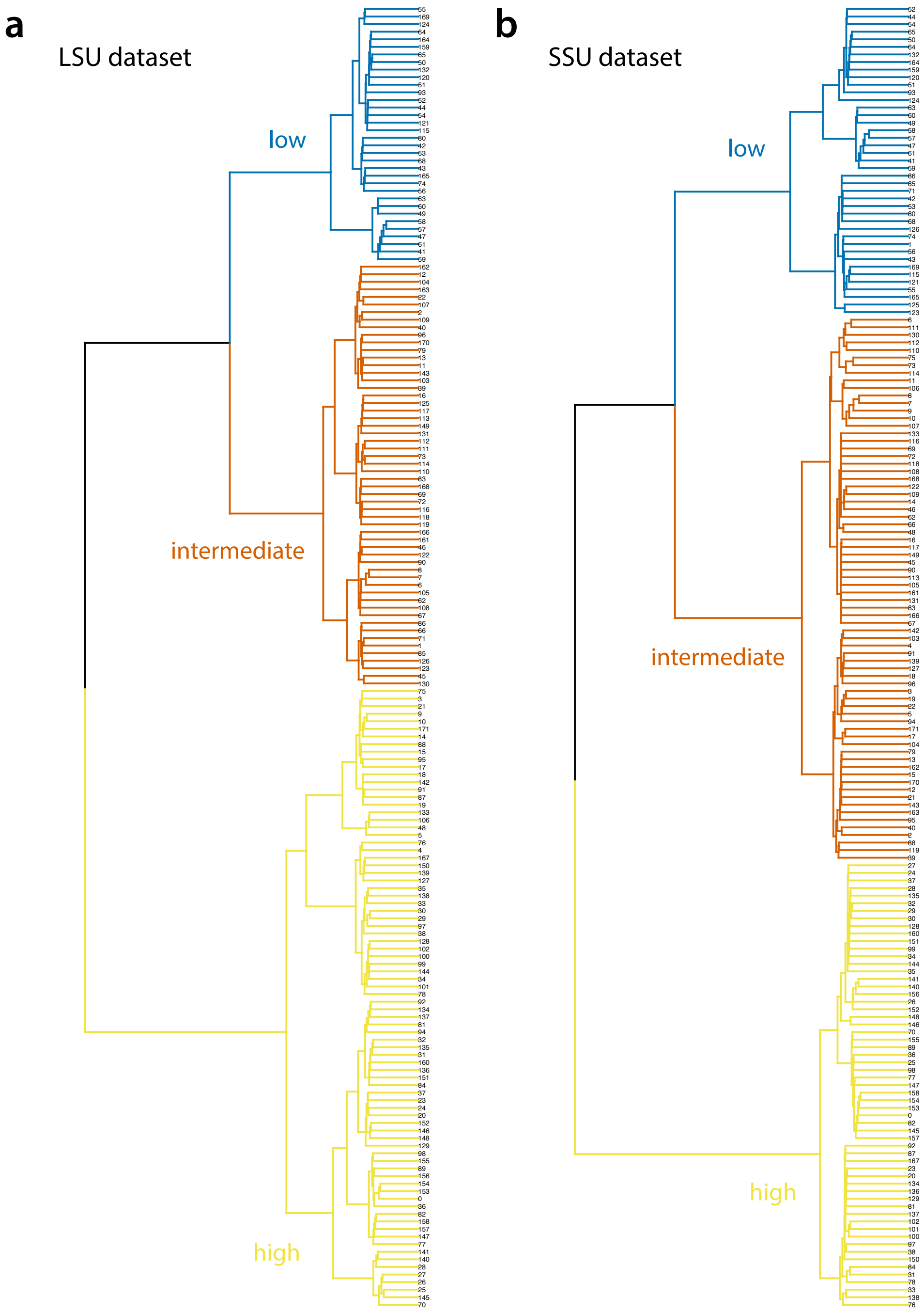

Supplementary Figure 8: Clustering of sites by community composition. Dendrogram of patrol areas in **(a)** the LSU dataset and **(b)** the SSU dataset based on posterior mean Jaccard distances clustered using Ward's criterion. Splitting the patrol areas into three groups, as shown here, produces clusters containing low-, intermediate- and high-elevation sites (see also Fig. 5 in the main text). Each branch represents a single patrol area, labelled with the same patrol area IDs used to identify sites in Supplementary Data 6.

LSU dataset

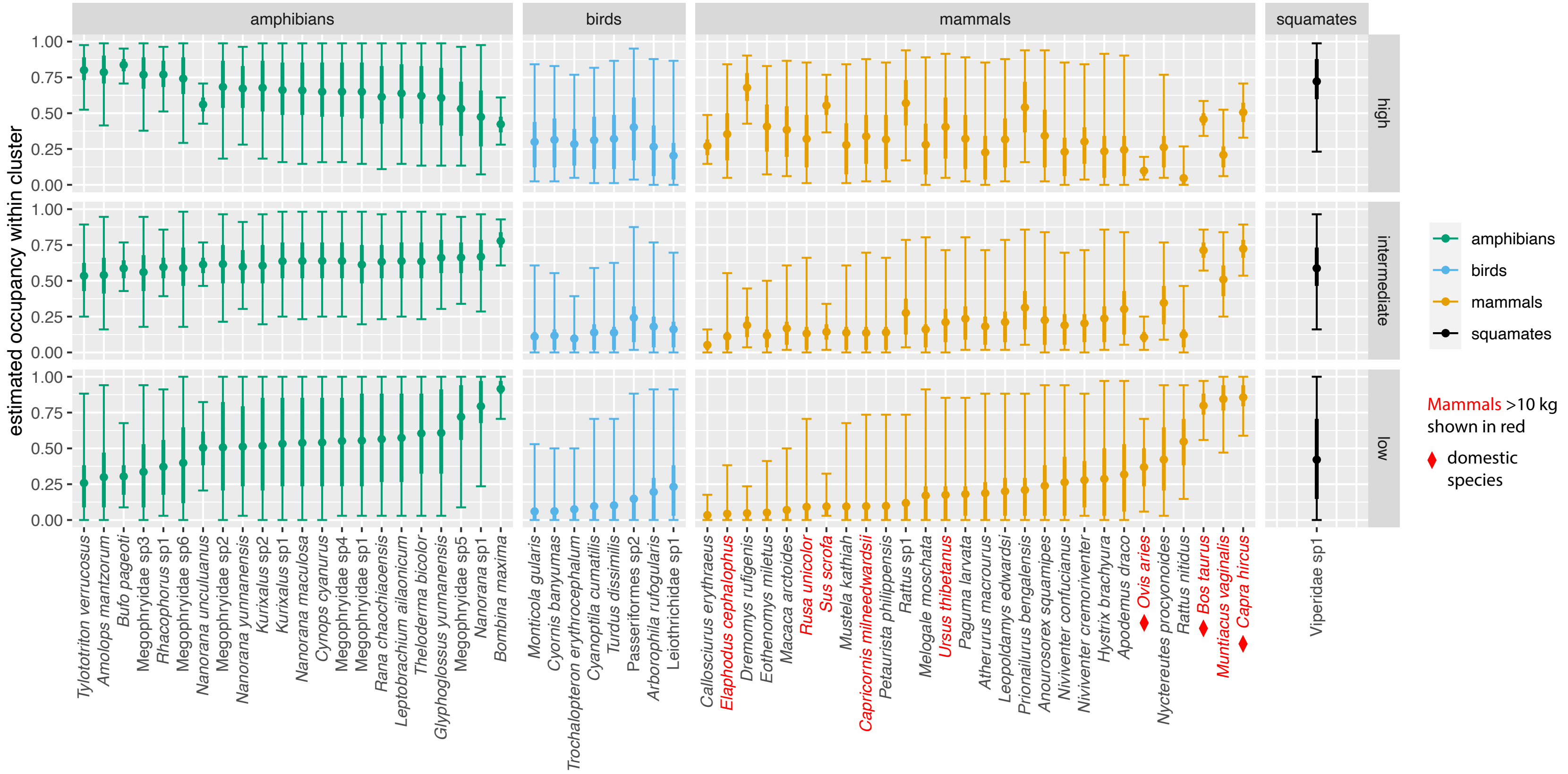

Supplementary Figure 9: LSU occupancy estimates by species. Estimated occupancy in high-, intermediate- and low-elevation patrol areas for species in the LSU dataset. For each species, plot shows posterior mean (dot), interquartile range (thick line) and 95% Bayesian confidence interval (BCI; thin line with crossbars) from model based on $n = 893$ replicate samples. Patrol areas were divided into high-, intermediate- and low-elevation by clustering based on Jaccard distances as shown in Fig. 5a,c and Supplementary Fig. 8a. Within taxonomic groups, species are ordered by occupancy in low-elevation sites. Species names for mammals over 10 kg adult body mass are shown in red. Domestic species are denoted with red diamonds.

SSU dataset

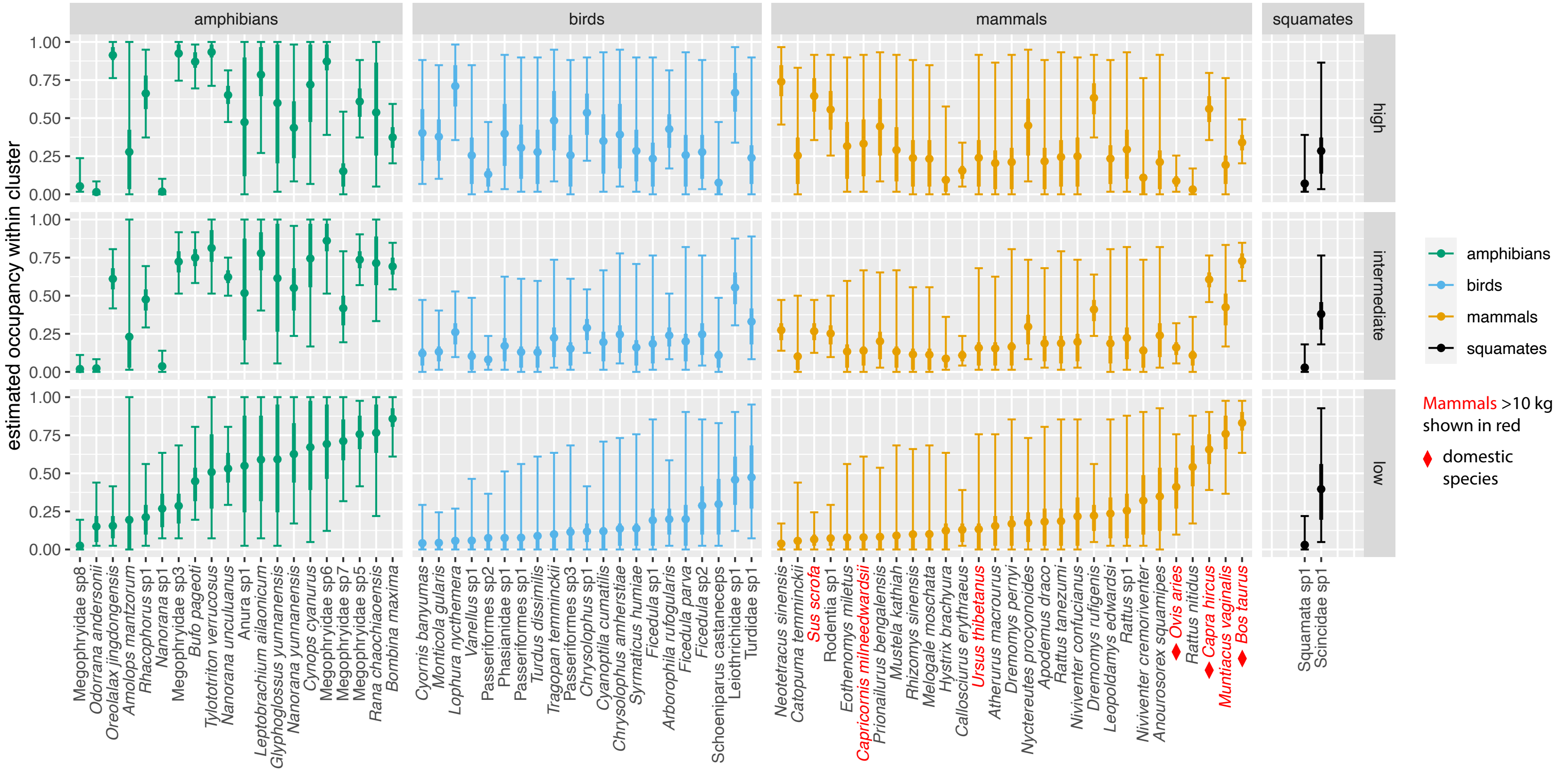

Supplementary Figure 10: SSU occupancy estimates by species. Estimated occupancy in high-, intermediate- and low-elevation patrol areas for species in the SSU dataset. For each species, plot shows posterior mean (dot), interquartile range (thick line) and 95% Bayesian confidence interval (BCI; thin line with crossbars) from model based on $n = 893$ replicate samples. Patrol areas were divided into high-, intermediate- and low-elevation by clustering based on Jaccard distances as shown in Fig. 5b,d and Supplementary Fig. 8b. Within taxonomic groups, species are ordered by occupancy in low-elevation sites. Species names for mammals over 10 kg adult body mass are shown in red. Domestic species are denoted with red diamonds.

5 Supplementary Note: Chinese Language Main Text

注：本中文版本仅包含正文的摘要、前言、结果、讨论、方法以及数据和脚本可获性的文字部分，其余部分请直接查阅英文版。

1. 摘要

建立自然保护区是实现生物多样性保护的核心措施，然而如何评估其保护效率仍然是一个难题。为了解决这一难题，我们首次利用蚂蝗吸食血液中的DNA进行了一次大规模的尝试，对占地677平方公里的位于中国西南部云南省的哀牢山国家自然保护区进行了一个全局的脊椎动物多样性的评估。在本研究中，该保护区被划分成172个巡逻区，由163位护林员在巡视过程中采集了总共30,468只蚂蝗，在这些蚂蝗的测序数据中，我们鉴定得到86个脊椎动物物种，包括两栖类、哺乳类、鸟类和爬行类。我们的多物种占据模型分析结果显示：在群落水平，物种丰富度随着海拔的升高以及与保护区边缘距离的拉长而增加;在物种水平，绝大多数大型野生哺乳动物(如水鹿、黑熊、苏门羚、黑麂)在较高海拔、靠近保护区中央的地区分布更多，而三个家养动物物种(牛、绵羊、山羊)和一个野生动物物种(赤麂)则在海拔较低的靠近保护区边缘的地区分布更多。本研究的结果显示基于融合了蚂蝗eDNA和多物种占据模型的技术可以为评估自然保护区对脊椎动物的保护效率创建一个直接的、高效的、可重复的、易于被大众接受理解的、并且可以被审计的结果指标，该指标可以用于评估保护区对脊椎动物多样性的保护效率，从而提高保护区对实现全球生物多样性目标的贡献。

2. 前言

2010年，《生物多样性公约》签署国通过了2011-2020年的爱知生物多样性目标[43]。其中，爱知目标11涉及了生物多样性的保护，要求截至2020年至少要将17%的陆地和内陆水域栖息地纳入保护区系统（如国家公园和其他自然保护区），并且该系统应当在生态上具有代表性、相互之间存在良好的关联性、以及有着公平和有效的管理。根据2018年世界自然保护联盟发布的报告显示，全球陆地面积的15%已被纳入保护区范围，这个数据已经接近爱知目标11的要求[44, 45, 46]。其中，中国作为公约签署国之一，其保护区的面积早在2008年就已经占到陆地国土面积的15%（约143万平方公里）[47, 48]。

为了实现爱知目标11，中国展示了相当大的行动力和国内的机构能力。例如，在中国西部地区，自然保护区几乎可以达到国际水平，涵盖包括了主要的生态区、生物多样性保护的优先区域以及多种天然植被类型[49]。并且基于遥感数据的研究结果显示，中国的自然保护区已经成功阻止了森林的滥砍滥发[50]。然而，在中国东南部的自然保护区其生态代表性相对较差[51]，许多保护区是隔离的[49]，并且关于这些保护区对当地人群的影响也所知甚少。更为重要的是，我们不知道这些保护区是否能够有效地保护生活在其中的物种。

然而，如何评估自然保护区的成效仍然存在巨大挑战，这个挑战是全球性的，即很难直接评估基于区域的保护工作是否取得了积极的生物多样性成果，以至于最近对保护区的审查认为其效果为“未知”[46]。目前只能采用一些间接方法，例如人员配置和预算充足性的评估（“投入评估”[46]），或者污染和人类压力等生物多样性威胁的评估（“威胁减少评估”[46]），在保护区区域，尤其是那些可以利用遥感等高通量技术的地方，进行保护区有效性的评估[44, 52, 53, 46]。然而，即使呈现了管理投入的部署和/或已知威胁的减少成功地产生了积极的生物多样性结果[46]，这些间接方法也并不能检测保护区在不同的物种类群之间是否存在不同的保护效果，也无法识别新的威胁。

因此，在本研究中，我们要解决的问题是：我们是否能够利用环境DNA（eDNA）来对脊椎动物生物多样性进行大规模的测量，并将之作为自然保护区保护效率的直接衡量标准。而我们关注脊椎动物（包括哺乳类、鸟类、两栖类和爬行类）的原因是，中国脊椎动物类群面临的最重要威胁之一就是过度开发[54]，而使用遥感方法并无法检测到这种威胁，因此特别难以评估。理想情况下，生物多样性评估应达到较高的空间和物种

分类分辨率，并允许在大范围内频繁更新，以便能够快速检测和定位野生动物种群的状态和变化，从而推断可能的原因采取补救措施，并对补救措施的有效性进行测试。其次，应该能够由独立的利益相关者和中立的第三方（比如法院）对评估结果的真实性和有效性进行严格验证，并且评估应该是直接的——即基于物种检测而不是其他间接方法——这对于争端的解决以及指导和激励有效管理都是必要的。最后，生物多样性的评估应是高效且易于被决策者和公众理解的，从而有助于当局政策的可持续性发展和管理结构的合法性[55, 56, 57]。

近年来随着红外相机和生物声学等技术的进步和广泛应用，使得我们可以在较大规模下开展生物多样性监测工作。然而，购买、部署和维护此类设备的成本仍然限制了它们能够监测的空间范围。例如，Beaudrot等人[58]利用相机技术在15个热带森林保护区对地栖哺乳类和鸟类的511个群落进行了长达3到8年的监测工作。然而，尽管他们的相机部署范围在每个保护区覆盖了140到320平方公里的面积，但这仅占其数据集中最大保护区的1-2%，推究其原因主要是因为目前的相机技术在野外动物监测的应用扩展方面存在一些限制：首先就是购买相机的成本较高，为了覆盖大规模空间尺度的保护区需要购买的相机成本往往是以百万为单位；其次就是除了先期相机网络的部署，后期还需要定期回到样点对相机进行维护，而那些地形复杂难以抵达的样地会为维护工作带来极大的困难；再就是，在整个监测过程中相机容易发生毁损和丢失，这样不仅造成财务上的损失，更为严重的是影响或破坏野外数据的采集[59, 60]。此外，相机技术和生物声学方法可能会错过相当大一部分的脊椎动物物种多样性。例如，两栖类、爬行类动物和许多鸟类都不会被相机捕捉到，而许多哺乳类、两栖类和爬行类则在生物声学监测中可能被遗漏。

正因为如此，eDNA技术在补充相机和生物声学技术方面显示出了巨大的潜力[61]，可以同时避免在监测过程中部署维护设备的困难、设备的损失、以及数据分析中物种分类偏差等后续问题。在本研究中，我们采用的是蚂蟥iDNA，作为新兴的一类eDNA样本[62]，iDNA被广泛运用于物种的检测和生物多样性的监测工作。iDNA是通过采集无脊椎动物——包括血吸性寄生虫（如蚂蟥、蚊子、螯蝇、蜱等）和以粪便为食的动物（如苍蝇、屎壳郎等）——来获得脊椎动物DNA的一种方法[63, 64, 65]。随着越来越多的研究工作通过iDNA对脊椎动物物种及其疾病的分布范围进行调查[66, 67, 68, 69, 70, 71]，加上与红外相机及其他方法的数据进行比较[72, 22, 73]，以及相关实验和分析流程的开发工作[15, 74]，iDNA技术在不断的改进和成熟。

我们在本研究中采用蚂蟥iDNA对位于中国西南部云南省、占地面积约677平方公里的哀牢山国家级自然保护区（图1）的脊椎动物分布进行了一次大规模的评估调查。该保护区建立于1981年，1984至1985年的调查公布了该保护区内86种哺乳类、323种鸟类、39种爬行类和26种两栖类动物的物种/亚物种清单[30]。此后，有研究人员进行了一次性的目标调查[75, 76, 77]和单个物种的研究[78, 79, 80, 81, 82]。该保护区近年来进行的一项基于红外相机的调查工作[83]检测到10种哺乳动物和10种鸟类，但该工作不够全面，仅调查了保护区的2个巡逻区（总共有172个巡逻区），无法对脊椎动物生物多样性进行普查评估。因此，仍然缺乏脊椎动物生物多样性的全局分布调查，保护区内脊椎动物物种的现状和种群变化趋势基本处于未知状态。

我们的研究测试了在真正的保护区管理环境中采用iDNA调查的可行性。基于蚂蟥的iDNA方法作为一种有潜力的大规模监测技术具有以下几点优势。首先，采集蚂蟥的人员不需要专门培训。哀牢山保护区分为172个“巡逻区”，每个巡逻区每月都有从邻近村庄雇佣的护林员进行巡视，这些护林员可以在雨季巡逻时采集蚂蟥样本，因此，我们能够以相对较低的成本对整个保护区进行样本采集。其次，蚂蟥iDNA数据可以为我们提供一种有效的方法来纠正物种检测过程中产生的一些错误，这主要包括假阴性（即未能检测到实际存在的物种）和假阳性（即当某物种实际不存在时却检测到其DNA）。例如，当一个物种在某个地点没有被蚂蟥吸到血时，就会出现假阴性；或者该物种的DNA没有被成功地扩增或正确地分类，也会产生假阴性。假阳性的来源可能包括样点之间的蚂蟥迁移，采集过程或实验室中的样品污染，测序或生物信息处理中产生的错误等。

统计模型可以用来解决这些假阴性和假阳性的错误检测问题。在本项目中，我们使用分层位点占据模型[29, 27]分析了iDNA的测序结果，该模型可以辨别每一个位点检测到的物种是否真实存在，并且也可以推测得到在某位点没有直接观察到的物种是否可能存在。位点占据模型的基本原理是通过分别估算每个位点存在某物种的概率以及该物种存在时能够被检测到的概率来推断每个物种真正存在的位置[29, 84]。而要估算这些概率主要依赖于重复抽样设计，需要在足够接近的空间和时间范围内进行重复采样，这样获得的数据才能反应物种分布的真实状况。在样本采集前，我们向每一个护林员发放多个密封袋，每个袋子中放置有多个样本采集管，要求在不同时间和地点采集的蚂蝗需要放置在不同的管子和袋子中[65]，通过这个方式我们可以获得重复采样的样本。

蚂蝗iDNA的第三个优势在于有可能获得更为广泛的物种类群，因为我们已经知道蚂蝗的食性范围很广，它会吸食包括小型和大型哺乳类、鸟类、爬行类和两栖类（包括树栖动物）等等动物的血液。因此，与红外相机和生物声学类方法相比，蚂蝗iDNA可以为我们提供一个范围更广的物种类群数据[85, 69, 70]。同时，DNA序列可以帮助我们区分一些视觉上难以辨别的物种[72]（虽然iDNA序列在某些类群上也存在着物种分辨的困难）。最后，有研究显示蚂蝗吸食的血液DNA在其体内保存长达4个月之后仍然能通过PCR扩增检测到[1]，从好的方面来说，这使得我们可以通过增加蚂蝗的采集量来获得其先前几个月吸食血液的信息，从而提高iDNA的检测效率，另一方面，蚂蝗iDNA的这一特性会降低脊椎动物检测的时空分辨率，因为在被采集捕获之前，这些蚂蝗以及它们几个月前所吸食血液的宿主可能会在采样区域之间发生长距离迁移[65]。

在本工作中，我们采用高通量条形码技术（metabarcoding）[86]来检测野外蚂蝗所吸食的脊椎动物宿主类群，并用占据模型来评估这些脊椎动物在中国云南省哀牢山自然保护区的整个分布情况。在分析中，我们进一步确定了与这些分布相关的环境因素。并且，我们的结果显示，通过蚂蝗iDNA数据可以获得更宽广的脊椎动物类群的分布模式，其中包括了那些很难被相机和生物声学方法检测到的物种。因此，通过本研究我们的结论是，通过蚂蝗iDNA可以获得脊椎动物物种的空间分布和环境相关信息，为我们提供一个衡量自然保护区有效性的评估指标，从而帮助我们优化保护区内的管理策略及相关措施。

3. 结果

3.1 样本采集和高通量条形码

哀牢山国家级自然保护区形状狭长，其山脊线走向由西北向东南延伸约125公里（约24.9°N 100.8°E至24.0°N 101.5°E），平均宽度仅6公里宽，海拔高度从422至3,157米，年降水量为1,000至1,860毫米，具体取决于海拔高度[87]（图1和补充材料中的图1a, b）。该保护区的主要植被为亚热带常绿阔叶林，其两侧为农业用地，位于各个方向的低海拔斜坡上。保护区边界5公里范围内有261个村庄[88]，估计人口超过20,000。

本研究中的样本包含了30,468只蚂蝗，分别由163位护林员于2016年的雨季（7月至9月）在整个保护区的172个巡逻区采集而来。这些蚂蝗最终被分成了893份重复样本，具体划分方式或划分原则请见方法部分。

我们对每一份重复样本进行了DNA提取和两个线粒体基因片段的PCR扩增：16S rRNA基因(*MT-RNR2*)和12S rRNA基因(*MT-RNR1*)。我们在下文中将分别以LSU（核糖体大亚基）和SSU（核糖体小亚基）来称呼这两个基因，我们这样做主要是为了避免和目前微生物研究广泛使用的16S基因相混淆，因为细菌的16S基因是和我们所用的12S（而非16S）基因同源。在对测序数据进行了生物信息处理后，我们使用参数扩展的数据扩充（parameter-expanded data augmentation）对LSU和SSU数据集的多物种位点占据模型（multispecies site-occupancy models ）[27, 28]分别进行估计，以排除物种检测过程中产生的假阴性和假阳性错误，从而鉴定识别我们数据中存在的真实生态模式。

3.2 脊椎动物物种

我们在LSU和SSU数据中总共检测到86种非人类的脊椎动物物种，其中LSU有59种，SSU有72种。虽然LSU引物是专门为哺乳动物设计的，但是LSU和SSU的引物都能扩增出两栖类、鸟类、哺乳类和爬行类的DNA，而SSU引物作为通用性的脊椎动物引物，扩增得到了更多的鸟类物种（图2a）。有45个物种在两个基因的数据中同时检测到，其中包括了那些通过在重复样本中的分布鉴定得到的物种（补充材料图S2），而只在LSU中检测到的物种有14个，SSU有27个。在这所有86个物种中，有58个可以给到物种水平的分类名（LSU 45个，SSU 50个）。表1和表2按照模型估算出的占有率列出了每个基因数据中的前20个物种。

我们在最终版的占据模型中使用的超级群落规模为$M = 200$，在LSU数据中推算出的哀牢山的总物种丰富度为119种，而在SSU数据中则为113种（图2b）。设置$M = 150$产生了相近的结果，而设置$M = 100$明显会对物种丰富度的估算产生限制。

在我们的数据中家养动物物种占了很大的比重（补充数据1），这与我们在实际考察中观察到的保护区内这些家养动物的放牧情况相吻合。在两个基因数据中，家牛（*Bos taurus*）都是发现频率最高的物种，几乎在半数的巡逻区中都能检测到；家养山羊（*Capra hircus*）也很常见，在近三分之一的巡逻区被检测到，家养绵羊（*Ovis aries*）在约6%的巡逻区被发现。而且绵羊的发现集中在保护区的东南部（隶属于新平县），正好与石屏青绵羊品种的主要繁殖区——石屏镇相邻。

在我们检测到的野生动物物种中，有一些被IUCN列为受威胁或接近威胁的类群（表3）。在哺乳动物中，有4个物种属于IUCN易危等级：亚洲黑熊（*Ursus thibetanus*）、中华鬣羚（*Capricornis milneedwardsii*）、水鹿（*Rusa unicolor*）和短尾猴（*Macaca arctoides*）。在两栖动物中，云南棘蛙（*Nanorana yunnanensis*）和双色棱皮树蛙（*Theloderma bicolor*）被列为濒危物种，而花棘蛙（*Nanorana maculosa*）、棘肛蛙（*Nanorana unculuanus*）和景东齿蟾（*Oreolax jingdongensis*）则处于易危状态。这些动物中的一些物种，特别是两栖类，在哀牢山分布广泛（表3和补充数据1），突出了该保护区在保护这些物种上的重要性。

我们将iDNA数据与昆明动物研究所相关专家提供的物种列表（补充数据2）进行比较，发现蚂蝗iDNA在检测哀牢山的哺乳类和两栖类方面比鸟类和爬行类更为成功。在哺乳类中，哀牢山的127个物种有34个被检测到，其中体型较大的目中均有约半数被检测到：偶蹄目（8/11）、食肉目（7/18）和非人灵长类（1/4）。而在体型较小的目中，我们检测到了41个啮齿目物种中的14种（包括2个豪猪物种，帚尾豪猪*Atherurus macrourus* 和马来豪猪*Hystrix brachyura*），在24个真盲缺目（Eulipotyphla）物种中检测到2种（及其相近种），但是没有检测到蝙蝠（0/25），兔子（0/1），穿山甲（0/1）和树（0/1）。我们还检测到应该划分到啮齿目中的2个未命名物种。在两栖类方面，已知无尾目（Anura）在哀牢山存在25个物种，其中有12种被我们检测到，而哀牢山存在的蝾螈科的2个物种也都被检测到。除此之外，我们还检测到属于无尾目的13个物种（根据现有参考数据无法鉴定给出物种名），其中2个属于树蛙属（*Kurixalus*），而树蛙属的物种并没有在之前的哀牢山调查中发现，但是该属广泛分布于云南（补充数据3）。在爬行类中，我们只发现了3种未命名物种，而哀牢山已知的爬行动物有39种。我们的物种中有一个属于有鳞目（Squamata），其它的则分别属于石龙子科（Scincidae）和蝰蛇科（Viperidae）。最后，在鸟类中，哀牢山已知鸟类有462个物种，我们只检测到12个，再加上10个只能鉴定到属或更高分类层的物种。有趣的是，在我们发现的能够鉴定到物种水平的12个物种中，有5个属于地面食性的鸟类和陆生的雉科（雉类和其相近种），另外7个属于兼有地面和林下食性的鸟类，而根据以往综合调查的报告来看，具有这种特征的鸟类在哀牢山一共也就发现了14种。鉴于我们的LSU和SSU引物对哺乳动物和鸟类都具有高扩增成功率B_c（详见方法中的分子实验部分），我们初步将不同物种类群之间检测率的差异归因于蚂蝗，即它们主要是由护林员在地面采集的，因此与非地面觅食的鸟类相比，本研究采集的蚂蝗吸食到蛙类血液的概率更高。

检测频率最高的物种在LSU和SSU数据中的估算占有率分别约为0.6和0.8（表1和表2）。然而，大多数分物种观察到的几率较低（观察到的巡逻区中位数：在LSU和SSU数据中分别是2和3个巡逻区）。这在许多物种中反映为低占有率和低检测率（图2c）（分布位点所占比例的中位数：LSU和SSU分别为0.33和0.24；每100只蚂蟥中的检测概率中位数：在LSU和SSU分别为0.02和0.08）。

3.3 物种丰富度

根据LSU数据估算的每个巡逻区的物种丰富度中值为32，SSU为27，相比之下，每个巡逻区观察到的物种丰富度中值在两个基因数据中分别为3和4种（补充材料图3a，b）。每个重复样本中观察到的物种丰富度中值在LSU数据中为1，在SSU中为2，而每个巡逻区的重复样本数中值在两个基因数据中分别为3和4。

在这两个基因数据中，每个巡逻区的物种丰富度的观察值和估算值之间存在着巨大差距，这凸显了对脊椎动物物种监测过程中产生的检测错误可能会对生物多样性评估产生一定程度的偏差。虽然估算的检测概率在不同物种之间存在很大差异，但是大多数物种的检测概率都很低，特别是在那些蚂蟥个体数较少的重复样本中（图3c-f）。这些结果说明了在使用iDNA进行生物多样性调查时纠正假阴性错误的重要性。

几乎半数的巡逻区没有相关的物种观测数据，要么是因为没有采集到蚂蟥，要么是因为采集回来的样本没有提供充分的位置信息（图3a,b；请注意，虽然此图不显示无位置信息的样本，但是我们的模型仍然采用了这些样本的数据）。我们的占据模型估算了缺失数据，因此无论有没有观察值，都可以对所有巡逻区的物种丰富度进行估算（图3c,d）。而两个基因数据的结果都显示了，哀牢山保护区的南部约三分之一区域具有最高的物种丰富度。

在群落水平上，物种更可能出现在海拔较高且（在较小程度上）远离保护区边缘的地方。这可以从两个方面看出来。首先，两个基因的数据都显示了保护区的估算物种丰富度随着海拔的升高而增加，而仅有LSU数据显示估算的物种丰富度随着与保护区边缘距离的增加而增加（图3e,f）。其次，两个数据中的群落平均占有率（方程式11和12）随着海拔的增加而增加（图4a,e），而在LSU数据中，在海拔高度保持不变的情况下，群落平均占有率随着到保护区边缘距离的增加而出现不太明显的升高趋势（图4c）。

物种丰富度在LSU和SSU数据之间显示了很好的一致性。两个基因数据中观察到的物种丰富度在每个重复样本（补充材料图4a）和每个巡逻区（补充材料图4c）均呈正相关。因此在预料之中的，两个数据之间的估算物种丰富度也呈紧密的正相关（补充材料图4e）。采样效率可以增加物种的检测率：蚂蟥数量越多的重复样本中往往包含更多的物种（补充材料图4b），重复样本数越多的巡逻区也一样（补充材料图4d）。然而，正如预期的那样，估算的物种丰富度并没有随着采样效率而增加，因为我们的模型在蚂蟥数量和重复样本的数量变化上做了补偿修正（补充材料图4f）。

在物种水平上，海拔高度（两个基因数据）和到保护区边缘距离（仅LSU数据）的影响在方向和强度上都有所不同（图4b,d,f）。在体重超过10公斤的哺乳类动物中，家牛（*B. taurus*）、家养绵羊（*O. aries*）、家养山羊（*C. hircus*）和赤麂（*Muntiacus vaginalis*）的占有率随海拔升高而降低（补充材料图5和7）。低海拔地区往往更靠近保护区边缘；然而，就群落平均占有率而言，距离保护区边缘的独立影响很小（补充材料图6）。相比之下，如毛冠鹿（*Elaphodus cephalophus*）、水鹿（*R. unicolor*）、中华鬣羚（*C. milneedwardsii*）、亚洲黑熊（*U. thibetanus*）以及野猪（*Sus scrofa*）等物种的居住概率随着海拔的升高而增加，因此更可能出现在保护区中心海拔较高的森林中（补充材料图5和7）。

大部分体型在10公斤以下的哺乳类动物物种，包括红颊长吻松鼠（*Dremomys rufigenis*）和猬（*Neotracus sinensis*），在保护区中心海拔更高的森林中具有更高的占有率（补充材料图5和7）。鸟类也有同样的倾向，即在海拔较高的地点有较高的居住率。另一方面，一些小型哺乳动物物种，如大足鼠（*Rattus nitidus*），在保护区边缘、海

拔较低的森林中分布较多。两栖类则表现出多元变化，一些物种如缅甸溪蟾（*Bufo pageoti*；IUCN近危）和景东齿蟾（*O. jingdongensis*；IUCN易危）更常见于海拔较高、交通不便的地区，但其他物种如大蹼铃蟾（*Bombina maxima*）则更常见于保护区边缘的低海拔森林中。

3.4 群落组成

在两个基因数据中，分别对所有样本进行层次聚类分析，结果显示所有样本按照其所在巡逻区的海拔高度（低、中、高海拔位点）发生了聚类（图5a、b和补充材料图8）。这种聚类分布在两组基因数据中具有高度的一致性（Cramer's $V = 0.79$，95%置信区间0.73-0.85）。海拔较高的区域往往位于保护区内部，尤其是在南部，与低海拔地区相比，包含更多人迹罕至的森林（补充材料图1a,i；按海拔高度划分的前25%个巡逻区到保护区边缘的平均距离±标准误差为1540 m ± 850 m，后25%的为830 m ± 390 m）。

低海拔巡逻区的群落以家牛（*B. taurus*）、山羊（*C. hircus*）、赤麂（*M. vaginalis*）和大蹼铃蟾（*B. maxima*）为优势物种（图6）。这些物种存在于大多数低海拔地区，和不到一半的高海拔地区。相比之下，缅甸溪蟾（*B. pageoti*）和景东齿蟾（*O. jingdongensis*）表现出相反的分布模式：即它们在大多数低海拔地区都不存在，但在大多数高海拔巡逻区都存在。事实上，相较于低海拔地区，许多两栖类和鸟类在高海拔地区的占有率会更高（补充材料图9和10）。不过还有一些物种，如棘肛蛙（*N. unculuanus*），在低海拔、中海拔和高海拔地区表现出相近的占有率（图6）。

通过比较位点群落组成在两个基因数据之间的变化，我们发现两组数据间存在显著的协同性（RV系数[89] 0.77，$p \leq 0.001$），这表明两个数据中存在大量的共享信号。而且两个数据的相似系数——Jaccard距离也呈现高度的相关性（Pearson相关$r = 0.94$, $p = 0.001$）。

4. 讨论

通过本研究，我们证明了基于蚂蝗iDNA的高通量条形码技术可以对脊椎动物生物多样性进行大规模、高空间分辨率的评估调查。我们的研究是迄今为止使用iDNA进行的空间分辨度最高、规模最广的生物多样性调查。蚂蝗采集由未经培训的护林员在为期2-3个月中进行，最终就可以获得整个面积为677平方公里的自然保护区的哺乳类和两栖类的分布信息，并且还可以兼获少量鸟类和爬行类的分布信息（图1）。我们的研究结果表明，哀牢山自然保护区（主要在其核心区域）为具有高保护价值的脊椎动物物种提供了保护空间。研究结果还展示了保护区由于人类活动（如农业、畜牧和偷猎）而引发退化的脆弱性（图3和图5）。本研究为哀牢山保护区提供了基于iDNA的脊椎动物生物多样性基线，未来的iDNA调查可以基于这个基线测试各个物种分布的变化，作为评估保护区的效率指标[58]。总的来说，我们的研究是关于在现实管理环境中使用iDNA监测的进展报告，并为今后的相关工作提供了改进的基础。

4.1 哀牢山的脊椎动物生物多样性

我们的iDNA调查发现了86种非人脊椎动物，包括哺乳类、两栖类、鸟类和爬行类。其中许多是常见的野生动物物种，或家养物种，如牛。我们的数据中还包括了许多不太常见的物种，一般来说，如果是没有针对性的传统调查，这些不常见的物种将无法被发现，其中包括15种被IUCN认定为受威胁或接近受威胁的物种（表3）。

占据模型的结果显示，哀牢山高海拔地区的脊椎动物物种丰富度最高。我们的结果可能反映了保护区内海拔较低、较易接近的区域存在较大的人为干扰（如狩猎、家畜疾病传染给野生动物、以及栖息地的改变），这可能导致许多低海拔野生动物物种在当地灭绝。或者，更具有迁移能力的物种可能已经将它们的栖息地从以前喜欢的低海拔地区转移到不太合适的高海拔地区，以逃避人类的入侵[61]。

海拔高度和到保护区边缘的距离是脊椎动物群落丰富度和组成的重要预测因子（图3e,f和图5a,b）。对单个物种分布的研究表明，许多物种，特别是鸟类和小型哺

乳类，在较高海拔和保护区核心区域的占有率较高。这些物种包括了几种在IUCN中列为受威胁或接近受威胁的物种：短尾猴（*Macaca arctoides*）、毛冠鹿（*E. cephalophus*）、水鹿（*R. unicolor*）、中华鬣羚（*C. milneedwardsii*）和亚洲黑熊（*Ursus thibetanus*）。这些物种中的一些或所有物种对保护区边缘的栖息地变化、偷猎等人类活动反应较为敏感，并且在保护区周边地区（尤其是有大量牲畜的地域）很容易发生人类和野生动物之间的相互冲突（如亚洲黑熊）。不过相比之下，也有一些野生物种，如赤麂（*M. vaginalis*），似乎在保护区边缘地区具有更高的居住率。

4.2 使用iDNA进行生物多样性监测

采用蚂蝗iDNA进行调查有2个关键的优势：（a）能够调查比其他方法更为广泛的脊椎动物物种分类群；（b）可以通过雇佣大量人员，只需要经过简单培训，即可进行大量的样本采集工作，这样可以节省时间和成本，使得定期的大规模生物多样性调查更加可行。然而，这些优势会因为以下一些因素被部分地抵消：后期实验室巨大的工作量（可以考虑通过自动化操作平台解决这个问题）；样本采集激励设计的挑战（具体见下文）；iDNA特有的抽样误差和偏差；以及与生物信息处理和统计建模相关的工作量。我们需要12人*月来计数蚂蝗、提取DNA和运行PCR，测序公司需要至少一个月来构建文库和进行测序。本研究中，因DNA提取、PCR和测序产生的消耗成本约为210,000元人民币（30,000美元），这还没算上引物的额外成本约80,000元人民币（12,000美元），这些实验成本足以让我们进行几次这种规模的样本采集。

样本采集激励的设计：

事实证明，由护林员进行采样是在整个自然保护区收集大量蚂蝗的可行方法。护林员一般是从保护区邻近村庄中雇用的。他们并不是直接和我们对接，在我们准备好样本采集的耗材后，由林业规划院的合作人员提前（2016年6月至7月）将这些耗材送至保护区的各个分站点，交给当地的护林员，口头发出相关指示，并在9月采集结束后取回样本。为了方便护林员操作以及后期的重复样本处理，我们为每个巡逻区分别准备了1个腰包，每个腰包中放置有多个密封袋和一张样本采集事项说明的文件，每个密封袋中装有多个采集管（内有样本保存液RNALater）以及1张信息采集卡片（上面已经设计好需要由采集人填写采集样本的相关信息，包括采集人姓名、身份证号、采样日期、采样地点、GPS信息等），通过这个方式很自然的要求护林员进行蚂蝗的重复采样而不需要多做解释[65]。这就使得在单个时间点收集每个巡逻区的蚂蝗重复样本成为可能，消除了物种占有率信息会随着时间变化而变化，从而导致不同时间点采集的样本不具有良好重复性的问题[72]。不过由于后勤原因，从不同的巡逻区进行的样本采集总共花了3个月的时间。

然而，我们对样本信息数据的收集不太成功，因为许多样本虽然有关于采集人护林员的信息，但没有关于巡逻区域的信息。未来需要考虑将提供样本信息这项任务作为付款的条件，一部分护林员（主要是资历较深的护林员）应该接受样本信息数据收集方面的培训。一个更长期的可能性是可以在护林员的手机上安装一个支持GPS的应用程序，用于收集采样地点的坐标。另一方面，我们的占据模型建模框架能够很好地处理适当数量的缺失数据，并且我们对制造虚假信息的动机需要进行谨慎处理。例如，我们决定不按蚂蝗数量或所使用的采集管数量付费，因为这可能会促使护林员在保护区以外的区域收集蚂蝗。我们发现采用基本酬劳，加上至少采集到一只蚂蝗的小奖金，这样的效果就很好，我们已经在其他的样本采集工作中使用了这种方式。我们预计今后在样本采集方面需要适当地增加人工酬劳。

iDNA的样本误差和偏差：

在我们的研究中有几个可能的错误来源。第一个是蚂蝗最后一次进食和我们取样之间的时间可能长达几个月[1]。虽然DNA存留时间长有助于我们对动物的发现，但这也意味着检测到的DNA不一定反映采集样本时的物种分布情况。动物宿主可能会在被吸食血液后、我们采集到蚂蝗之前离开该采样巡逻区，如果携带蚂蝗的宿主（如可以长途旅行的鸟类）会广泛分散[90]，这可能会模糊检测结果的时空分辨率。不过我们的数据显示，我们采集的蚂蝗主要以可能停留在一个巡逻区内或最多在相邻区域之间

移动的宿主为食（例如青蛙），因此我们关于哀牢山野生和家养物种的总体分布结果（图3和图5）不太受这种偏差的严重影响。此外，以三个月为时间窗口在一个位置采集所有重复样本，这样就限制了蚂蝗或宿主长距离迁移的可能，因为物种长距离迁移会违反占据模型的前提假设，即物种分布在重复样本中应当保持不变（“种群闭合”假设[91, 65]）。不过也因为如此，采样时间的延长就限制了蚂蝗iDNA检测不太适用于非常快速变化的情况，例如在几个月内发生的事件[65]。

错误的第二个来源可能是蚂蝗群落在巡逻区之间的系统差异以及蚂蝗物种之间不同的饮食偏好。例如，如果蚂蝗物种随着海拔不同而发生变化（我们没有将其作为检测协变量），而高海拔的蚂蝗物种更倾向于吸食蛙血而非牛血，这将使这些物种检测到的分布会随着海拔发生变化。我们样本中蚂蝗数量过多，这使得我们无法对每一只蚂蝗进行单独鉴定，而且我们采样的地理位置和蚂蝗的统一形态与所有属于山蛭属（*Haemadipsa*）的蚂蝗一致[70]，该属的物种鉴定本就存在困难。已知山蛭以多种脊椎动物为食[70, 69]，这很可能是由于它们的习性决定（因为它们是机会主义、静坐等待的寄生虫），有研究表明，它们之间的饮食差异非常有限[70, 72, 66]。有鉴于此，我们选择了“蚂蝗汤”的方案，而不是试图将蚂蝗的个体特征和饮食偏好考虑在内，我们认为蚂蝗饮食的差异不可能解释我们研究中的任何主要结果。

第三个可能的错误来源是PCR引物和遗传标记的选择，这可能会造成某些物种虽然其DNA存在于样本中，但是因为PCR阶段无法被扩增从而无法被我们检测到。在本工作中，我们采用两个基因标记的数据部分地解决了这个问题。两个基因标记都检测到一半以上的物种，并且两个基因数据之间的物种丰富度和群落组成存在显著的协同相关性，这也就是说，即使我们只选择了其中一个基因，关于物种的总体分布结果也不会受到严重影响（图3和图5）。另一方面，不同引物在扩增特定物种DNA的能力上会出现明显差异。例如，我们在三个不同的巡逻区的LSU数据中检测到了短尾猴（*M. arctoides*），对应序列数分别为2,700、170,066和245,477条。相比之下，在SSU的相应数据中却没有发现可以分配给非人灵长目的序列。使用更多的引物或者更多的基因标记可能会检测到更多的物种分类群[92]，但是额外增加的实验/测序成本很可能和所得到回报不成正比。在未来，使用核酸探针和/或宏基因组测序[93]，或使用多重CRISPR-Cas13检测的CARMEN新方法[94]，可能会取代PCR。例如，这些方法都允许我们使用国际通用的动物条形码基因——细胞色素C氧化酶I（COI），其参考数据库更为全面[95]，同时还可以将其它遗传标记用于COI不能很好区分的物种分类群。

最后，蚂蝗iDNA会自然排除其所吸食血液宿主中没有很好代表性的物种分类群。有研究表明，与红外相机相比，许多物种的iDNA检测效率较低，尽管iDNA似乎更适合检测体型较小的哺乳类物种[1, 22, 73, 96, 61]，我们的研究表明两栖动物也属于此类情况。当然，如果有足够多的样本，检测率低的物种仍然可以被发现，它们的低检测率问题可以用位点占据模型来解决。未被检测到的物种仍然可以用于统计建模（例如，使用数据扩充[27, 28]），但它们显然无法为模型提供数据。当蚂蝗采样受到限制时，例如由研究人员进行样本采集时，Abrams等人[72]建议可以使用蚂蝗iDNA来对红外相机监测数据进行补充。例如，Tilker等人[61]最近在越南和老挝的五个保护区内的139个位点（17393 相机数*天数）进行了一次红外相机调查，调查范围为900 平方公里，并使用来自其中93个位点的2,043只蚂蝗的iDNA进行数据补充。红外相机数据检测到23种陆生哺乳动物，其中松鼠和大型啮齿动物是能够观察到的最小物种类群，而且这些类群通常会产生更多的检测数据。而在该工作中，蚂蝗iDNA检测到了相机数据中没有发现的云猫（*Pardofelis marmorata*），并将长颌带狸（*Chrotogale owstoni*）和亚洲黑熊（*U. thibetanus*）的检测率提高了近一倍。另一方面，不需要检测到一个区域中存在的每一个物种，而仍然能够识别得到广适性的生态模式。例如，Gogarten等人发现，相机数据和基于苍蝇的iDNA数据所检测到的群落基本上不重叠（在所有样点中，两种方法发现的共享物种为6% 43%）[96]，但这两类数据都对栖息地进行了相似的聚类划分。

多物种位点占据模型:

位点占据模型可以用于确定在单个物种水平上检测率和占有率之间的相关性。然而大多数物种检测率很低，在单独分析时它们对检测率和占有率能够提供的信息很少，因

为很难区分低检测率（即隐匿性）和低占有率（即稀有性）。然而，通过将这些低检测率的物种数据整合到占据和检测的群落模型中，共享物种和巡逻区域之间的信息，那么整个数据就能够为我们提供一个更为完整的哀牢山脊椎动物多样性的概貌。这种建模方式很好地处理了缺失数据，证明了在贝叶斯框架中占据模型在处理不完善数据方面的有效性。然而，另一方面，应用于大数据量的数据扩展模型就意味着对处理数据的计算机平台有较高的硬件要求，并且需要花费较长的运行时间以及先期建模时成功拟合模型所需的大量测试。

在本研究中，我们将建模重心集中在纠正假阴性错误上，当然要纠正假阳性错误也是可能的，例如由于实验室污染或分类鉴定错误造成的假阳性。虽然在我们的数据中，假阴性可能比假阳性更严重，但假阳性可能会导致生物多样性评估发生严重的偏差[97]。原则上，分层模型也可用于纠正假阳性错误，但在实践中，在没有关于假阳性检测处理的额外信息的情况下，很难对其效果进行评估[98]。虽然最近有研究显示有望进行假阳性的检测（例如[99]），但这些方法还不适用于多物种高通量条形码数据。

随着iDNA方法越来越多地用于大规模的生物多样性调查，在工作开展之前有一个需要考虑的重要实验设计问题——即如何合并蚂蝗样本。考虑到将蚂蝗按个体分开保存需要用到大量的采集管和相关耗材，因此合并样本可以减少采集成本和采集任务的复杂性。（蚂蝗会反刍到保存液中，因此收集到同一采集管中的蚂蝗不能分开作为独立的重复样本处理，如果按照个体进行分析就需要一开始为单个蚂蝗使用单独的采集管。）合并样本同样还可以减少后期的实验成本和工作量。不过从另一方面来看，使用非合并样本的数据时，占据模型效果会更好。而且如果单独处理蚂蝗，则可以对其进行DNA条形码鉴定，从而获得蚂蝗物种饮食偏好的信息，该信息可以在占据建模中用作检测协变量。开发自动化、高通量的实验方案（如[94]）将有助于解决实验工作量的问题，使得在类似我们这样的大样本（即>30,000个个体）工作中可以对蚂蝗的个体分别进行处理。在样本采集阶段也可以考虑一个折衷方案，就是向采集者提供比本研究中所用更小的采集管（例如2毫升），以降低每管的蚂蝗数量，但不一定能达到单个蚂蝗的水平。

4.3 iDNA：一个有潜力的生物多样性监测工具

当我们准备用一个2020年以后的新框架取代爱知生物多样性目标时，有呼吁要将重心放在如何开发使用生物多样性指标（如物种的占有率、丰度和种群变化趋势）来直接评估保护区的成效上[100, 46, 101]。然而，许多保护区资源不足，人力不足[44]，生物多样性的监测工作很难被优先考虑[46]。在本研究中，我们展示了使用基于iDNA的高通量条形码技术作为一种经济有效的方法来评估整个自然保护区内、具有高空间分辨率、覆盖更多物种类群的脊椎动物生物多样性的可行性。因此，我们的工作证明了iDNA可以作为一个有潜力的生物多样性监测工具，直接对保护区的保护成效进行评估。

除了得到检测物种的分布图谱外，我们的工作还可以指导未来的监测工作，以确定环境变化、人类活动的影响、以及造成整个野生动物群落动态变化的可能原因。我们建议使用我们的结果来指导在哀牢山进行其他方式的生物多样性调查工作，包括对目标物种粪便的采集、红外相机和生物声学监测调查的工作方案设计等，后两种方法都可以独立对我们的结果进行测试验证，并且通过共同能检测到的物种（如哺乳类、地栖鸟类）来提高物种占有率和检测率的估算准确性[72]。同时这些监测方法也可以通过使用占据模型框架来估算某些物种的种群规模和种群变化趋势[102, 103, 104]。此外，我们还进一步提出，iDNA可以用于调查生物多样性的其他方面，如人畜共患病。最近的研究表明可以利用蚂蝗样本（从野外取样）获取野外病毒及其脊椎动物宿主的相关信息[105, 71]。2020年的全球新冠大流行凸显了加快了解野生动物病毒库中人畜共患病的紧迫性——而随着全球气候变暖和土地利用的持续变化，这一需求可能变得更加急迫[106]。

5. 方法

本节主要是对方法的概述。补充材料中提供了蚂蝗采集、实验室处理、生物信息学分析流程和位点占据模型建模的详细说明。生物信息学分析代码见[107]和[24]。统计建模和分析代码见[31]。

5.1蚂蝗样本的采集

本研究中的样本由哀牢山自然保护区的护林员于2016年7月至9月的雨季采集。整个自然保护区被划分为172个无重叠的巡逻区。这些区域的面积从0.5平方公里到12.5平方公里（平均3.9 ± sd 2.5平方公里），面积的大小在某种程度上反映了这些巡逻区可抵达的难易度（通常较小的区域更加崎岖难以抵达）。这些巡逻区在本研究之前就已经确立，并用于日常保护区的管理。保护区根据行政区划分为6个部分，由6个市或自治县（南华、楚雄、景东、镇远、双柏、新平）管理，根据邻近程度为其管辖范围内的村庄分配巡逻区。各村庄成立工作组，在巡逻区内开展工作。因此，每个护林员可能每年都会更换，但巡逻区域和负责巡逻的村庄是固定的。

我们向每个护林员提供多个密封袋，每个密封袋都装有多个样本采集管（其中装有DNA/RNA保存剂）。护林员被要求将巡逻时看到的蚂蝗（如从地面或衣服上）放入采集管中，可以在每个采集管中放入多条蚂蝗，但采集管尺寸有限，这样通常需要护林员使用多个管子进行蚂蝗的采集。

本次工作中，总共有163名护林员在所有172个巡逻区内于3个月中收集了30,468只蚂蝗。当一个密封袋中的蚂蝗总数小于100只时，我们将其中所有采集管的蚂蝗集中起来，作为一份样本处理，从而减少实验室的工作量和成本。而当一个密封袋里装着蚂蝗的数目⩾100只时，我们将其中的蚂蝗按照采集管合并起来，生成5份蚂蝗数目大致相等的重复样本，以避免任何样本中包含有过多的蚂蝗。在本次工作中，有81%的密封袋中含有< 100只蚂蝗，有78%的巡逻区仅存在低于阈值的密封袋。各个巡逻区通常返回多个含有< 100只蚂蝗的密封袋。在经过样本的合并处理之后，每份样本的蚂蝗平均数量为34只（范围为1到98只），总共有893份样本。

5.2 环境特征

我们使用ArcGIS Desktop 9.3（Esri，Redlands，CA）和R v3.4.0[37]来计算每个巡逻区域的特征。我们为海拔高度、地形位置指数（即每个像素与其周围像素之间的差值[108]）、到最近道路的距离以及到最近河流的距离创建了30米的栅格图层。然后，我们计算每个巡逻区域的栅格值中值，作为统计建模中的预测值（表4和补充材料图1）。我们还计算了每个巡逻区质心到最近自然保护区边缘的距离。

5.3 分子实验

我们从每份样本中提取DNA，然后用PCR扩增了两个线粒体基因的标记：一个来自16S rRNA（*MT-RNR2*）基因（引物：*16Smam1* 5'-CGGTTGGGGTGACCTCGGA-3'和*16Smam2* 5'-GCTGTTATCCATCCAGGTAACT-3'[2]），另一个来自12S rRNA（*MT-RNR1*）基因（引物：5'-ACTGGGATTAGATACCCC-3'和5'-YRGAACAGGCTCCTCTAG-3'由[3]修改而得）。下文中我们将这两个标记分别称为LSU（16S，82-150bp）和SSU（12S，81-117bp），这两个标记分别指的是这些基因编码的核糖体的大亚基和小亚基。（我们这样做是为了避免与广泛使用的细菌16S基因混淆，该基因与我们的12S标记物同源，而不是与我们的16S标记物同源。）我们也对细胞色素C氧化酶I基因COX1 [4]进行了测试，但是本文中没有采用，因为该基因的引物可以扩增蚂蝗DNA，因此返回的脊椎动物数据极少。

LSU引物是专门针对哺乳动物设计的，而SSU引物则是针对所有脊椎动物设计的。我们运用`ecoPCR v0.5` [109]，以MIDORI数据库[18]中的四足动物（Tetrapoda）的序列为参考数据，在允许3个错配的情况下估算LSU和SSU引物的预期扩增成功率Bc值。B_c值是在生物信息分析中可以被扩增的物种在参考数据中所占的比例。正如预期的那样，LSU引物对哺乳类（99.3%）和鸟类（96.2%）的B_c值较高，对两栖类（79%）的B_c值适中，而对MIDORI数据库中“爬行类”分类的物种（鳄鱼目+蝶

骨目+角鲨目+龟鳖目）（39.9%）的B_c值较低。SSU引物对哺乳类、两栖类和鸟类的B_c值较高（$> 98\%$），对"爬行类"的B_c值适中（79.8%）。因此，我们预计大部分或所有哀牢山的哺乳类、鸟类和两栖动物都可以通过一个或两个引物进行扩增，而蛇和蜥蜴等动物的成功率较低。

我们在订购引物时将样本识别标签设计在引物的5'端，采用双向标签策略，使用DAMe [6]来识别和消除"标签跳跃"产生的错误[5]。在893份样本中，我们用LSU引物成功扩增了661份样本，而使用SSU引物成功扩增了745份样本。成功的PCR扩增产物被送到Novogene（中国北京）进行PCR-free文库构建，并在Illumina HiSeq X Ten上进行PE150 bp测序。

每组PCR均包括阴性对照组，如果琼脂糖凝胶检测显示阴性对照组中存在污染，则重复或最终放弃该组PCR产物。并且我们还对阴性对照进行了测序，因为凝胶并不能检测到非常低水平的污染。在我们的阴性对照产物中出现了人、牛、狗、山羊、猪、鸡和一些野生物种的序列，但PCR扩增效率极低，得到的序列数也很少。我们使用这些阴性对照的数据在生物信息学分析（见下一节和补充材料）中为所有样本的数据在DAMe中设置过滤参数，用于删除数据中可能存在的污染序列：LSU和SSU的参数`y`均设为2（即在3次PCR中至少有2次PCR都出现的序列才被保留），LSU的参数`t`设为9，SSU设为20（`t`是保留序列在每次PCR中必须出现的最小拷贝数）。我们还扩增和测序了一些阳性对照产物，其模板由两种啮齿动物的DNA组成，这两个物种是*Myodes glareolus*和*Apodemus flavicollis*，它们仅分布在欧洲和西亚，而在我们的蚂蝗数据中并没有检测到。

5.4 生物信息学分析流程

我们的生物信息学分析流程有三个关键特征：首先是DAMe方案[6]，该方案采用双向标签和3次独立的PCR扩增来识别和去除"标签跳跃"和其它可能因素产生的错误序列；其次，是我们使用两个独立的基因，对检测到的物种分类进行双重检查（补充材料图2）；最后一点，是我们采用PROTAX方法[16, 17]来进行物种分类的确定，该方法可以减少在参考数据库未完善时鉴定物种分类时很容易发生的过度自信偏差。在本工作中，大约有一半的已知哀牢山物种存在于参考数据库中（补充材料数据2）。其中，哺乳类和两栖类的代表性相对较好：在LSU数据库中，有73%的哺乳类和83%的两栖类物种存在参考序列，而在SSU数据库中哺乳类和两栖类的存在率分别为70%和67%。鸟类和爬行类物种的代表性较差，LSU数据库中仅有42%的鸟类和53%的爬行类存在，SSU数据库中有35%和34%的鸟类和爬行类。对于没有匹配到参考数据的OTU序列，PROTAX将其分配到更高等级的分类单位（如属、科、目等），并将其物种标记为"未知"，使我们可以根据其他信息（比如之前提及的调查数据或者另一个基因数据的相应信息）将这些OTU分配到可能的形态物种。

在DAMe过滤后，我们使用VSEARCH v2.9.0 [12]去除残余的PCR重组序列，使用Swarm v2.0 [13]将序列聚类成初步的可操作分类单元（"pre-OTUs"），然后使用R软件包中的`LULU` v0.1.0 [14]将这些具有高度相似性和相同样本分布的"pre-OTUs" 进行合并。然后，我们使用PROTAX将合并后的OTUs代表序列进行物种分类匹配[15, 16, 17]，其中我们所使用的参考数据是在MIDORI数据库的基础上增加了最近发表的东南亚哺乳动物线粒体基因组序列[19]。所有细节请详见补充文件。为了共享LSU和SSU数据集的物种分类信息，我们计算了619份样本（在这些样本中，LSU和SSU基因都得到了成功的扩增和测序）中两个基因"pre-OTUs"之间的两两相关系数，并将其可视化为一个网络图（补充材料图2）。如果一个LSU和一个SSU的"pre-OTUs"出现在同一批样本中，并且匹配到同一类物种分类单元中，则我们认为2个基因的该对"pre-OTUs"具有很大的几率是从同一组以同一物种为食的蚂蝗中扩增出来的。我们手动检查了网络图，并将这些相关的"pre-OTUs"代表序列对分配给同一个物种分类。

我们删除了所有无法匹配到任何物种（以及更高级别分类单位）的"pre-OTUs"，这些"pre-OTUs"仅分别占LSU和SSU数据集的0.9%和0.2%，并且极有可能是测序产生的错误序列，而非新的物种序列。在LSU和SSU数据集中，我们合并了具有相同物种分

类鉴定的"pre-OTUs"，从而为每个数据集生成最终的可操作性分类单元集（OTUs）。最后，在统计建模分析之前，我们从两个基因的数据集中删除了被鉴定为人(*Homo sapiens*)的OTUs。虽然这些人的OTUs可以为绘制整个保护区的人类分布提供信息，但我们预计大部分的人DNA是来自护林员本身，而不是分布在保护区的其他人。

虽然因为参考数据库的不完善而导致一些OTUs无法被鉴定到物种水平，但是我们仍然倾向于将最终获得的OTUs在物种水平上进行诠释。因此，我们采用sp来指代物种名，例如，LSU数据集中的2个蛙的OTUs—*Kurixalus* sp1和*Kurixalus* sp2—应解释为两种不同的*Kurixalus*物种；同样的，LSU和SSU数据集中的蛙OTU—Megophryidae sp—应被解释为Megophryidae中的某一个物种。因此，在本研究接下来的分析中，我们将最终的OTUs匹配分类结果均称为物种。

在删除鉴定为人的序列后，最终LSU和SSU数据中分别包含18,502,593和84,951,011条序列。这些序列代表了LSU数据集在126个巡逻区的653份样本中的59个物种，以及SSU数据集在127个巡逻区的740份样本中的72个物种。为了评估我们的iDNA方法能够检测到的脊椎动物多样性的广度，我们将iDNA检测到的物种列表与昆明动物研究所其他研究人员的未发表的哀牢山调查物种列表进行了比较。

我们还在物种列表中附加了额外的数据：我们通过使用R软件包中的`rredlist` v0.6.0 [25]搜索PROTAX确定的物种学名，附加了国际自然保护联盟（IUCN）关于这些物种的数据。为此，根据最近的研究和国际自然保护联盟（IUCN）的最新评估[110, 111]，我们将*Capricornis milneedwardsii*视为*Capricornis sumatraensis*的同义词。对于哺乳类，我们使用PanTHERIA数据库[26]获取每个物种的成年体重数据；而在物种水平信息不可用的情况下，我们采用了该OTU在数据库中最近分类单位（如属、科等）中所有物种的成年体重的平均值。

5.5 位点占据模型

我们使用参数扩展的数据扩充（parameter-expanded data augmentation）来分别估计LSU和SSU数据集的多物种位点占据模型（multispecies site-occupancy models）[27, 28]。这些模型假设：LSU数据中检测到的59个物种($n_{\text{LSU}} = 59$)和SSU中检测到的72个物种($n_{\text{SSU}} = 72$)分别是哀牢山物种群落中能够通过蚂蝗iDNA捕捉到的子集。尽管N_{LSU}和N_{SSU}是未知的，但是我们可以通过将它们嵌入到一个固定大小为M的"超级群落"来进行建模。我们在最终模型中将群落大小设为200($M = 200$)。从$M = 150$到$M = 474$的值（后者是1984至1985年哀牢山调查工作[30]中获得的哺乳类、鸟类、爬行类和两栖类的总物种丰富度）得出了N_{LSU}和N_{SSU}的近似估计值。

对于超级群落中的每一个物种，我们的模型明确地捕捉到以下三方面的信息：（i）"群落过程"（community process）的信息，即确定某物种是否存在于哀牢山群落中；(ii)"生态过程"（ecological process）的信息，即假设某物种存在于哀牢山群落中，那么它在每个巡逻区中是否存在；以及（iii）"观察过程"（observation process）的信息，即假定某物种存在于某巡逻区内，那么我们能否在该巡逻区的样本中检测到该物种的DNA。通过采用群落级参数和先验值将每个物种的群落、生态和观测过程三方面信息联系在一起，具体步骤如下所述：

在群落过程中，假设每一个物种i在哀牢山群落中要么存在，要么不在。我们用w_i来表示这种未确定的状态，w_i被假定为被群落成员参数Ω_{g_i}所控制的伯努利随机变量（Bernoulli random variable），即物种i在哀牢山群落中存在的概率：

$$w_i \sim \text{Bernoulli}(\Omega_{g_i}). \tag{9}$$

在群落过程中，我们将物种分为两个自然组——恒温的哺乳类和鸟类，以及变温的两栖类和爬行类——这样就可以允许它们在哀牢山群落中具有不同的存在概率。这点由Ω_{g_i}参数的下标表示，其中g_i表示物种i属于这两类中的哪一类。这个方式的分析结果显示了我们的预期，即这两个自然组在群落中存在的概率上确实有差异，因此在接下来进

行生态和观测过程的分析中，我们采用了相同的分组法来设置参数（进一步讨论见下文）。

在生态过程中，假设每个物种i 在每个巡逻区j 中存在或不存在，我们使用z_{ij} 表示这种未确定的生态状态。我们假设从巡逻区j 采集的所有重复样本的z_{ij} 是恒定的，即在同一个时间点所采集到的重复样本中z_{ij} 是相同的。假设存在的任何物种都是哀牢山群落的成员（即$w_i = 1$），因此我们建模时将z_{ij} 设置为同时受w_i 和占据参数ψ_{ij}（群落中的物种i 存在于巡逻区j 的概率）控制的伯努利随机变量：

$$z_{ij}|w_i \sim \text{Bernoulli}(w_i\psi_{ij}). \tag{10}$$

在LSU数据中，我们在建模时将占据参数ψ_{ij} 设为海拔高度和到最近保护区边缘距离的函数

$$\text{logit}(\psi_{ij}) = \beta_{0i} + \beta_{1i}\text{elevation}_j + \beta_{2i}\text{reserve}_j \tag{11}$$

而在SSU数据集中只设置了海拔高度的函数

$$\text{logit}(\psi_{ij}) = \beta_{0i} + \beta_{1i}\text{elevation}_j \tag{12}$$

其中，elevation_j是巡逻区j 的海拔高度中间值，reserve_j是巡逻区j 的质心到最近保护区边缘的距离。我们通过为每个数据集运行包含所有5个环境协变量的“完整”模型来选择这些变量，并仅保留斜率系数不为0的贝叶斯置信区间在95%以内的协变量。

我们将观测过程建模为伯努利过程，并假定检测不完全，但没有假阳性：

$$y_{ijk}|z_{ij} \sim \text{Bernoulli}(z_{ij}p_{ijk}), \tag{13}$$

其中，y_{ijk} 是观察到的数据，即在巡逻区j 的重复样本k 中检测到或未检测到物种i 的DNA。

我们将条件检测概率p_{ijk} 设为r_i（即物种i 在每100只蚂蝗中被检测到的概率）和leeches_{jk}（重复样本中的蚂蝗数量）的函数：

$$p_{ijk} = 1 - (1 - r_i)^{\text{leeches}_{jk}/100} \tag{14}$$

$$\text{logit}(r_i) = \gamma_{0i} \tag{15}$$

我们允许r_i及其对数值γ_{0i} 在物种之间可以变动，以捕捉蚂蝗因为取食偏好在不同物种分类群中的变化。我们使用$\text{leeches}_{jk}/100$而不是leeches_{jk}来避免由于四舍五入而产生的计算问题。

需要注意的是，检测概率p_{ijk} 是由物种i 是否存在于巡逻区j 中决定的，而不是物种i 的DNA是否存在于该巡逻区的重复样本k 中决定的。因此，检测概率p_{ijk} 主要受到两大类因素的影响，一类是会造成样本中缺少物种i 的DNA的因素（例如，重复样本中的蚂蝗没有吸食到物种i 的血液，或者它们很久以前吸食了物种i 的血液，但是DNA已经被消化了），另一类是虽然存在物种i 的DNA，然而因为其它因素导致没有检测到的情况（例如PCR扩增失败、PCR或测序发生了错误、生物信息处理过程中出现了问题等）。我们对每个重复样本进行了多次PCR（具体见方法部分），这在原则上可以将p_{ijk} 做以下的分解（i）当物种i 存在于某巡逻区时，该物种的DNA存在于该巡逻区重复样本中的概率；（ii）当物种i 的DNA存在于重复样本中时，通过PCR检测到其DNA的概率，这个概率可以通过在模型中添加另一个分级层次来获得[112, 113, 114, 115]。我们首先使用DAMe [6]将每份样本多次重复PCR的结果结合起来，然后再进行建模，这是因为DAMe是专门设计用于检测和消除PCR和测序中所产生的错误，并为我们提供了专门适用于本项目数据的过滤选项。

最后，虽然方程式9到15仅定义了物种i 的位点占据模型，但我们将这些物种特异的模型和生态及检测过程的群落模型结合起来：

$$\beta_{1i} \sim \mathrm{N}(\mu_{\beta_1}, \sigma_{\beta_1}) \tag{16}$$

$$\beta_{2i} \sim \mathrm{N}(\mu_{\beta_2}, \sigma_{\beta_2}) \quad \text{(for the LSU model only)} \tag{17}$$

$$(\beta_{0i}, \gamma_{0i}) \sim \mathrm{MVN}([\mu_{\beta_0 g_i}, \mu_{\gamma_0 g_i}], \begin{bmatrix} \sigma^2_{\beta_0 g_i} & \rho\sigma_{\beta_0 g_i}\sigma_{\gamma_0 g_i} \\ \rho\sigma_{\beta_0 g_i}\sigma_{\gamma_0 g_i} & \sigma^2_{\gamma_0 g_i} \end{bmatrix}) \tag{18}$$

其中N( )和MVN( )分别表示正态分布和多元正态分布。这些分布以$\mu_\bullet$ 和$\sigma_\bullet$为群落超参数，每个参数的分布由第一个下标表示。我们使用多元正态先验$(\beta_{0i}, \gamma_{0i})$来允许物种占据和检测概率之间的非零协方差，例如，我们可以预测到物种丰度的变化是否会影响到这两种概率[27]。

这些群落模型可以允许稀有物种有效地从常见物种借用信息，从而产生更好的整体参数评估值，不过代价是个体参数的缩减[34, 33, 27]。至于在上述的群落过程分析中，我们将物种分为两组——恒温的哺乳类和鸟类，以及变温的两栖类和爬行类——并允许它们具有不同的群落分布。这点由占有率和检测率的截距—$\mu_\bullet$和$\sigma_\bullet$群落超参数的下标表示，其中g_i表示物种i属于这两组中的哪一组。这种方法反映了我们的预期，即这些群体在占据概率（例如，由于不同的栖息地偏好）和检测概率（例如，由于与蚂蝗的接触几率不同，或是由于蚂蝗的饮食偏好）方面会有系统上的不同。另一种分类也可以从生物学的角度进行论证：例如，根据许多哺乳类是陆生的，而许多鸟类是树栖的，将哺乳类和鸟类分开；或者将鸟类和爬行类组合在一起，以更好地反映系统发育关系。这些替代分组在我们的数据中表现不佳，因为大多数鸟类和爬行类的观察率太低，无法为这类分组提供充分的信息，但未来值得对这种分类法的建模重新进行研究。

我们使用JAGS v4.3.0 [36]的贝叶斯框架（参数：5 chains of 100,000 generations, including a burn-in of 50,000）对我们的模型进行了估算。除了需要保存z矩阵以进行beta多样性和聚类占有率计算的情况外，我们为后验样本保留了所有轮数（见下面的统计学分析）；由于内存的限制，我们无法为z矩阵保留所有后验样本，而为了使这些计算可行，我们将样本稀释了十倍。补充材料中提供了用于模型参数的先验分布的详细信息。根据模型结果，我们为所有涉及的模型参数计算了后验均值和分位数，并估算了每个巡逻区的物种丰富度和每个物种占据的位点数。

5.6 统计学分析

物种丰富度：

对每个基因数据，我们通过对w_i求和，直接从模型中获得了哀牢山所有物种丰富度的估计值。为了评估对M的选择，我们比较了$M = 100$、150和200的所有物种丰富度的估值。

在检查了每个物种的占有率和检测率估值后，我们使用直方图来可视化每个巡逻区估算物种丰富度的分布（通过对z_{ij}求和得到每个巡逻区的j）。我们计算了整个巡逻区的估算物种丰富度中位数，以和每个巡逻区和每个重复样本中观察到的物种丰富度中位数进行比较。我们绘制了choropleths分级着色图以可视化整个自然保护区内观察到和估算物种丰富度的空间分布。

我们检查了群落平均占有率和检测概率（参见[116]中的第11.7.2节），以帮助了解位点和样本协变量的影响。对于每个物种组$g = 1, 2$（分别代表哺乳类/鸟类和两栖类/爬行类），我们计算了群落平均占有率和检测率的后验平均值和95%贝叶斯置信区间，作为协变量的函数：

$$\psi_g(\text{elevation}) = logit^{-1}(\mu_{\beta_0 g} + \mu_{\beta_1}\text{elevation}) \tag{19}$$

$$\psi_g(\text{reserve}) = logit^{-1}(\mu_{\beta_0 g} + \mu_{\beta_2}\text{reserve}) \quad \text{(for the LSU model only)} \tag{20}$$

$$p_g(\text{leeches}) = 1 - (1 - \text{logit}^{-1}(\mu_{\gamma_0 g}))^{\text{leeches}/100} \tag{21}$$

这种方法有效地在函数ψ_g(elevation)中将到保护区边缘的距离保持为零，而在函数ψ_g(reserve)中将海拔高度保持为零，对应于我们数据中这些协变量的平均值，因为预测值在建模之前是标准化的。为了可视化物种间的占有率和检测率对协变量的反应，我们使用每个物种对β_0, β_1, β_2和γ_0的估计值取代群落超参数来重复这些计算，以获得每个物种的后验平均值。

为了评估两个基因数据对哀牢山物种丰富度变化的一致程度，我们比较了两个数据之间物种丰富度的三个度量。第一个是每个重复样本中观察到的物种丰富度；第二个是各巡逻区观察到的物种丰富度；第三个是每个巡逻区的估算物种丰富度（即根据z_{ij}计算的物种后验平均值）。对于每一个度量，我们计算了数据之间的皮尔逊相关性，并用t检验测试了相关系数与零的关系。我们还使用泊松广义线性模型（Poisson GLMs）检查了每个物种丰富度测量值与采样效率之间的关系：我们将每个重复样本的观察物种丰富度与每个重复样本的蚂蝗数的对数值进行回归分析，将每个巡逻区的观察和估算物种丰富度与每个巡逻区的样本数的对数值进行回归分析，并用t检验测试斜率系数的显著性。

群落组成：

我们使用基于估算占有率状态z_{ij}计算得到的Jaccard相似度后验均值来探索巡逻区之间脊椎动物群落组成的变化（该方法的其他示例见Dorazio[28] 和Kéry and Royle[116]）。使用非度量多维标度（NMDS）排序图可视化Jaccard距离矩阵（即distance = $(1 - \text{similarity})$），使用vegan中的Ordsurf函数覆盖了环境协变量。使用Ward标准（R函数`hclust(., method = "ward.D2")`）根据Jaccard距离对巡逻区进行聚类。基于聚类的结果，巡逻区被分成三组，分别对应于低海拔、中海拔和高海拔的区域。我们使用Cramer's V来量化两个基因数据的聚类匹配度。我们通过在哀牢山地图上根据这三个聚类对各个巡逻区进行着色，从而可视化保护区内的群落组成的空间变化。为了帮助理解脊椎动物群落在这些聚类区域之间是如何变化的，我们使用占有率状态z_{ij}的后验样本来计算每个物种在低海拔、中海拔和高海拔聚类区域中的占有率（即存在巡逻区的比例）的后验均值和95%贝叶斯置信区间。

为了评估这两个基因数据所鉴定的巡逻区之间群落组成变化模式的共享程度，我们使用R中的`ade4::coinertia` 对每个数据中每个巡逻区的预测物种矩阵进行了协惯性分析。我们使用RV系数[89]量化了协惯性，在`ade4::RV.rtest`中用999种排列测试其显著性。我们还使用999种排列的Mantel测试检验了Jaccard距离后验均值在两个基因数据之间的相关性。

6. 数据可获性

本工作所用的Illumina HiSeq/MiSeq测序数据均已上传至NCBI的SRA（Sequence Read Archive）数据库中，对应的BioProject accession number是PRJNA624712 [https://www.ncbi.nlm.nih.gov/bioproject/?term=PRJNA624712] 。处理后的数据以OTU-和宏数据表格在补充材料数据6中提供，同时也可以从GitHub网站我们的占据模型分析脚本中获得(https://github.com/bakerccm/leeches-public/releases/tag/v1.1; doi:10.5281/zenodo.5914708)。我们所用的MIDORI数据可以从http://www.reference-midori.info下载。增加的线粒体基因组参考数据则来自Salleh等人2017的论文(GigaScience 6(8): gix053)，具体见其文中的表1和表2所列的NCBI编号，(文章链接为https://academic.oup.com/gigascience/article/6/8/gix053/3958782)。PanTHERIA数据可以从https://doi.org/10.6084/m9.figshare.c.3301274.v1获得。昆明动物所相关工作的物种列表在补充材料数据2和数据3中提供。

7. 脚本可获性

我们的生物信息学分析（从原始数据处理得到OTUs）脚本可以从https://github.com/jiyinqiu/ailaoshan_leeches_method_code [107]中获得。物种分类鉴定脚本可见https://github.com/dougwyu/screenforbio-mbc-ailaoshan/releases/tag/1.3 [24]。位点占据模型的分析代

码请见https://github.com/bakerccm/leeches-public/releases/tag/v1.1 (doi:10.5281/zenodo.5914708) [31]。

References

[1] Ida Bærholm Schnell, Philip Francis Thomsen, Nicholas Wilkinson, Morten Rasmussen, Lars R.D. Jensen, Eske Willerslev, Mads F. Bertelsen, and M. Thomas P. Gilbert. Screening mammal biodiversity using DNA from leeches. *Current Biology*, 22(8):R262 – R263, 2012.

[2] P G Taylor. Reproducibility of ancient DNA sequences from extinct Pleistocene fauna. *Molecular Biology and Evolution*, 13(1):283–285, 1996.

[3] Tiayyba Riaz, Wasim Shehzad, Alain Viari, François Pompanon, Pierre Taberlet, and Eric Coissac. ecoPrimers: inference of new DNA barcode markers from whole genome sequence analysis. *Nucleic Acids Research*, 39(21):e145–e145, 2011.

[4] Matthieu Leray, Joy Y Yang, Christopher P Meyer, Suzanne C Mills, Natalia Agudelo, Vincent Ranwez, Joel T Boehm, and Ryuji J Machida. A new versatile primer set targeting a short fragment of the mitochondrial coi region for metabarcoding metazoan diversity: application for characterizing coral reef fish gut contents. *Frontiers in Zoology*, 10:34, 2013.

[5] Ida Bærholm Schnell, Kristine Bohmann, and M. Thomas P. Gilbert. Tag jumps illuminated – reducing sequence-to-sample misidentifications in metabarcoding studies. *Molecular Ecology Resources*, 15(6):1289–1303, 2015.

[6] M. L. Zepeda-Mendoza, K. Bohmann, A. Carmona Baez, and M. T. Gilbert. DAMe: a toolkit for the initial processing of datasets with PCR replicates of double-tagged amplicons for DNA metabarcoding analyses. *BMC Research Notes*, 9:255, 2016.

[7] Tom van der Valk, Francesco Vezzi, Mattias Ormestad, Love Dalén, and Katerina Guschanski. Index hopping on the Illumina HiseqX platform and its consequences for ancient DNA studies. *Molecular Ecology Resources*, 20(5):1171–1181, 2020.

[8] Mikkel Schubert, Stinus Lindgreen, and Ludovic Orlando. AdapterRemoval v2: rapid adapter trimming, identification, and read merging. *BMC Research Notes*, 9:88, 2016.

[9] J N Joshi and N A Fass. *Sickle: a sliding-window, adaptive, quality-based trimming tool for FastQ files (version 1.33)*, 2011.

[10] Heng Li. BFC: correcting illumina sequencing errors. *Bioinformatics*, 31(17):2885–2887, 2015.

[11] Andre P Masella, Andrea K Bartram, Jakub M Truszkowski, Daniel G Brown, and Josh D Neufeld. PANDAseq: paired-end assembler for Illumina sequences. *BMC Bioinformatics*, 13(1):1–7, 2012.

[12] Torbjørn Rognes, Tomáš Flouri, Ben Nichols, Christopher Quince, and Frédéric Mahé. VSEARCH: a versatile open source tool for metagenomics. *PeerJ*, 4:e2584, 2016.

[13] F. Mahe, T. Rognes, C. Quince, C. de Vargas, and M. Dunthorn. Swarm v2: highly-scalable and high-resolution amplicon clustering. *PeerJ*, 3:e1420, 2015.

[14] T. G. Frøslev, R. Kjøller, H. H. Bruun, R. Ejrnæs, A. K. Brunbjerg, C. Pietroni, and A. J. Hansen. Algorithm for post-clustering curation of DNA amplicon data yields reliable biodiversity estimates. *Nature Communications*, 8:1188, 2017.

[15] Jan Axtner, Alex Crampton-Platt, Lisa A Hörig, Azlan Mohamed, Charles C Y Xu, Douglas W Yu, and Andreas Wilting. An efficient and robust laboratory workflow and tetrapod database for larger scale environmental DNA studies. *GigaScience*, 8(4):giz029, 2019.

[16] P. Somervuo, S. Koskela, J. Pennanen, R. H. Nilsson, and O. Ovaskainen. Unbiased probabilistic taxonomic classification for DNA barcoding. *Bioinformatics*, 32(19):2920–2927, 2016.

[17] P. Somervuo, D. W. Yu, C. C. Y. Xu, Y. Q. Ji, J. Hultman, H. Wirta, and O. Ovaskainen. Quantifying uncertainty of taxonomic placement in DNA barcoding and metabarcoding. *Methods in Ecology and Evolution*, 8(4):398–407, 2017.

[18] Ryuji J. Machida, Matthieu Leray, Shian-Lei Ho, and Nancy Knowlton. Metazoan mitochondrial gene sequence reference datasets for taxonomic assignment of environmental samples. *Scientific Data*, 4:170027, 2017.

[19] Faezah Mohd Salleh, Jazmín Ramos-Madrigal, Fernando Peñaloza, Shanlin Liu, S Sinding Mikkel-Holger, P Patel Riddhi, Renata Martins, Dorina Lenz, Jörns Fickel, Christian Roos, Mohd Shahir Shamsir, Mohammad Shahfiz Azman, K Lim Burton, J Rossiter Stephen, Andreas Wilting, and M Thomas P Gilbert. An expanded mammal mitogenome dataset from Southeast Asia. *GigaScience*, 6(8):1–8, 2017.

[20] Scott Chamberlain, Eduard Szoecs, Zachary Foster, Zebulun Arendsee, Carl Boettiger, Karthik Ram, Ignasi Bartomeus, John Baumgartner, James O'Donnell, Jari Oksanen, Bastian Greshake Tzovaras, Philippe Marchand, Vinh Tran, Maëlle Salmon, Gaopeng Li, and Matthias Grenié. *taxize: taxonomic information from around the web*, 2019. R package version 0.9.7.

[21] Alexey M Kozlov, Jiajie Zhang, Pelin Yilmaz, Frank Oliver Glöckner, and Alexandros Stamatakis. Phylogeny-aware identification and correction of taxonomically mislabeled sequences. *Nucleic Acids Research*, 44(11):5022–5033, 2016.

[22] Torrey W Rodgers, Charles C Y Xu, Jacalyn Giacalone, Karen M Kapheim, Kristin Saltonstall, Marta Vargas, Douglas W Yu, Panu Somervuo, W Owen McMillan, and Patrick A Jansen. Carrion fly-derived DNA metabarcoding is an effective tool for mammal surveys: Evidence from a known tropical mammal community. *Molecular Ecology Resources*, 17(6):e133–e145, 2017.

[23] Szymon M Kiełbasa, Raymond Wan, Kengo Sato, Paul Horton, and Martin C Frith. Adaptive seeds tame genomic sequence comparison. *Genome Research*, 21(3):487–493, 2011.

[24] Doug Yu. Ailaoshan version with unweighted and weighted PROTAX and MIDORI 1.2, 2020.

[25] Scott Chamberlain. rredlist: 'IUCN' red list client, 2018. R package version 0.6.0.

[26] Kate E. Jones, Jon Bielby, Marcel Cardillo, Susanne A. Fritz, Justin O'Dell, C. David L. Orme, Kamran Safi, Wes Sechrest, Elizabeth H. Boakes, Chris Carbone, Christina Connolly, Michael J. Cutts, Janine K. Foster, Richard Grenyer, Michael Habib, Christopher A. Plaster, Samantha A. Price, Elizabeth A. Rigby, Janna Rist, Amber Teacher, Olaf R. P. Bininda-Emonds, John L. Gittleman, Georgina M. Mace, and Andy Purvis. PanTHERIA: a species-level database of life history, ecology, and geography of extant and recently extinct mammals. *Ecology*, 90(9):2648–2648, 2009.

[27] R. M. Dorazio, J. A. Royle, B. Soderstrom, and A. Glimskar. Estimating species richness and accumulation by modeling species occurrence and detectability. *Ecology*, 87(4):842–854, 2006.

[28] Robert M. Dorazio, Nicholas J. Gotelli, and Aaron M. Ellison. *Modern methods of estimating biodiversity from presence-absence surveys*, pages 277–302. InTech, Rijeka, Croatia, 2011.

[29] Darryl I MacKenzie, James D Nichols, Gideon B Lachman, Sam Droege, J Andrew Royle, and Catherine A Langtimm. Estimating site occupancy rates when detection probabilities are less than one. *Ecology*, 83(8):2248–2255, 2002.

[30] Investigation Group of Ailaoshan Nature Reserve. *Comprehensive survey of Ailaoshan Nature Reserve*. Yunnan Ethnic Press, Kunming, Yunnan, 1988.

[31] Christopher CM Baker, Yinqiu Ji, Viorel D Popescu, Jiaxin Wang, Chunying Wu, Zhengyang Wang, Yuanheng Li, Lin Wang, Chaolang Hua, Zhongxing Yang, Chunyan Yang, Charles CY Xu, Alex Diana, Qingzhong Wen, Naomi E Pierce, and Douglas W Yu. Measuring protected-area vertebrate biodiversity using leech iDNA. GitHub repository. [https://github.com/bakerccm/leeches-public/releases/tag/v1.1] [doi:10.5281/zenodo.5914708], 2021.

[32] B. J. Callahan, P. J. McMurdie, M. J. Rosen, A. W. Han, A. J. A. Johnson, and S. P. Holmes. DADA2: high-resolution sample inference from Illumina amplicon data. *Nature Methods*, 13(7):581–583, 2016.

[33] William A. Link and John R. Sauer. Extremes in ecology: avoiding the misleading effects of sampling variation in summary analyses. *Ecology*, 77(5):1633–1640, 1996.

[34] Donald B. Rubin. Bayesianly justifiable and relevant frequency calculations for the applied statistician. *The Annals of Statistics*, 12(4):1151–1172, 1984.

[35] Andrew Gelman. Prior distributions for variance parameters in hierarchical models. *Bayesian Analysis*, 1(3):515–533, 2006.

[36] Martyn Plummer. JAGS: A program for analysis of Bayesian graphical models using Gibbs sampling, 2017. Version 4.3.0.

[37] R Core Team. *R: A Language and Environment for Statistical Computing*. R Foundation for Statistical Computing, Vienna, Austria, 2019.

[38] Martyn Plummer. rjags: Bayesian graphical models using MCMC, 2018. R package version 4.8.

[39] Ken Kellner. *jagsUI: A wrapper around 'rjags' to streamline 'JAGS' analyses*, 2019. R package version 1.5.1.

[40] Andrew Gelman and Donald B. Rubin. Inference from iterative simulation using multiple sequences. *Statistical Science*, 7(4):457–472, 1992.

[41] Stephen P. Brooks and Andrew Gelman. General methods for monitoring convergence of iterative simulations. *Journal of Computational and Graphical Statistics*, 7(4):434–455, 1998.

[42] Gábor Csárdi and Tamás Nepusz. The igraph software package for complex network research. *InterJournal Complex Systems*, page 1695, 2006.

[43] Convention on Biological Diversity. Aichi Biodiversity Targets, 2010.

[44] Lauren Coad, James EM Watson, Jonas Geldmann, Neil D Burgess, Fiona Leverington, Marc Hockings, Kathryn Knights, and Moreno Di Marco. Widespread

shortfalls in protected area resourcing undermine efforts to conserve biodiversity. *Frontiers in Ecology and the Environment*, 17(5):259–264, 2019.

[45] James E. M. Watson, Emily S. Darling, Oscar Venter, Martine Maron, Joe Walston, Hugh P. Possingham, Nigel Dudley, Marc Hockings, Megan Barnes, and Thomas M. Brooks. Bolder science needed now for protected areas. *Conservation Biology*, 30(2):243–248, 2016.

[46] Sean L Maxwell, Victor Cazalis, Nigel Dudley, Michael Hoffmann, Ana S L Rodrigues, Sue Stolton, Piero Visconti, Stephen Woodley, Naomi Kingston, Edward Lewis, Martine Maron, Bernardo B N Strassburg, Amelia Wenger, Harry D Jonas, Oscar Venter, and James E M Watson. Area-based conservation in the twenty-first century. *Nature*, 586(7828):217–227, 2020.

[47] W. H. Xu, Y. Xiao, J. J. Zhang, W. Yang, L. Zhang, V. Hull, Z. Wang, H. Zheng, J. G. Liu, S. Polasky, L. Jiang, Y. Xiao, X. W. Shi, E. M. Rao, F. Lu, X. K. Wang, G. C. Daily, and Z. Y. Ouyang. Strengthening protected areas for biodiversity and ecosystem services in china. *PNAS*, 114(7):1601–1606, 2017.

[48] B. A. Bryan, L. Gao, Y. Q. Ye, X. F. Sun, J. D. Connor, N. D. Crossman, M. Stafford-Smith, J. G. Wu, C. Y. He, D. Y. Yu, Z. F. Liu, A. Li, Q. X. Huang, H. Ren, X. Z. Deng, H. Zheng, J. M. Niu, G. D. Han, and X. Y. Hou. China's response to a national land-system sustainability emergency. *Nature*, 559(7713):193–204, 2018.

[49] Ruidong Wu, Hugh P Possingham, Guangzhi Yu, Tong Jin, Junjun Wang, Feiling Yang, Shiliang Liu, Jianzhong Ma, Xi Liu, and Haiwei Zhao. Strengthening China's national biodiversity strategy to attain an ecological civilization. *Conservation Letters*, 68(2):e12660, 2019.

[50] Guopeng Ren, Stephen S. Young, Lin Wang, Wei Wang, Yongcheng Long, Ruidong Wu, Junsheng Li, Jianguo Zhu, and Douglas W. Yu. Effectiveness of China's National Forest Protection Program and nature reserves. *Conservation Biology*, 29(5):1368–1377, 2015.

[51] Ruidong Wu, S Zhang, Douglas W Yu, P Zhao, X Li, Longzhu Wang, Qian Yu, Jian Ma, Ai Chen, and Yongcheng Long. Effectiveness of China's nature reserves in representing ecological diversity. *Frontiers in Ecology and Evolution*, 9:383–389, 2011.

[52] Jonas Geldmann, Andrea Manica, Neil D Burgess, Lauren Coad, and Andrew Balmford. A global-level assessment of the effectiveness of protected areas at resisting anthropogenic pressures. *PNAS*, 116(46):23209–23215, 2019.

[53] William F Laurance, D Carolina Useche, Julio Rendeiro, Margareta Kalka, Corey J A Bradshaw, Sean P Sloan, Susan G Laurance, Mason Campbell, Kate Abernethy, Patricia Alvarez, Víctor Arroyo-Rodríguez, Peter Ashton, Julieta Benitez-Malvido, Allard Blom, Kadiri S Bobo, Charles H Cannon, Min Cao, Richard Carroll, Colin Chapman, Rosamond Coates, Marina Cords, Finn Danielsen, Bart De Dijn, Eric Dinerstein, Maureen A Donnelly, David Edwards, Felicity Edwards, Nina Farwig, Peter Fashing, Pierre-Michel Forget, Mercedes Foster, George Gale, David Harris, Rhett Harrison, John Hart, Sarah Karpanty, W John Kress, Jagdish Krishnaswamy, Willis Logsdon, Jon Lovett, William Magnusson, Fiona Maisels, Andrew R Marshall, Deedra McClearn, Divya Mudappa, Martin R Nielsen, Richard Pearson, Nigel Pitman, Jan van der Ploeg, Andrew Plumptre, John Poulsen, Mauricio Quesada, Hugo Rainey, Douglas Robinson, Christiane Roetgers, Francesco Rovero, Frederick Scatena, Christian Schulze, Douglas Sheil,

Thomas Struhsaker, John Terborgh, Duncan Thomas, Robert Timm, J Nicolas Urbina-Cardona, Karthikeyan Vasudevan, S Joseph Wright, Juan Carlos Arias-G, Luzmila Arroyo, Mark Ashton, Philippe Auzel, Dennis Babaasa, Fred Babweteera, Patrick Baker, Olaf Banki, Margot Bass, Inogwabini Bila-Isia, Stephen Blake, Warren Brockelman, Nicholas Brokaw, Carsten A Brühl, Sarayudh Bunyavejchewin, Jung-Tai Chao, Jerome Chave, Ravi Chellam, Connie J Clark, José Clavijo, Robert Congdon, Richard Corlett, H S Dattaraja, Chittaranjan Dave, Glyn Davies, Beatriz de Mello Beisiegel, Rosa de Nazaré Paes da Silva, Anthony Di Fiore, Arvin Diesmos, Rodolfo Dirzo, Diane Doran-Sheehy, Mitchell Eaton, Louise Emmons, Alejandro Estrada, Corneille Ewango, Linda Fedigan, François Feer, Barbara Fruth, Jacalyn Giacalone Willis, Uromi Goodale, Steven Goodman, Juan C Guix, Paul Guthiga, William Haber, Keith Hamer, Ilka Herbinger, Jane Hill, Zhongliang Huang, I-Fang Sun, Kalan Ickes, Akira Itoh, Natália Ivanauskas, Betsy Jackes, John Janovec, Daniel Janzen, Mo Jiangming, Chen Jin, Trevor Jones, Hermes Justiniano, Elisabeth Kalko, Aventino Kasangaki, Timothy Killeen, Hen-biau King, Erik Klop, Cheryl Knott, Inza Koné, Enoka Kudavidanage, José Lahoz da Silva Ribeiro, John Lattke, Richard Laval, Robert Lawton, Miguel Leal, Mark Leighton, Miguel Lentino, Cristiane Leonel, Jeremy Lindsell, Lee Ling-Ling, K Eduard Linsenmair, Elizabeth Losos, Ariel Lugo, Jeremiah Lwanga, Andrew L Mack, Marlucia Martins, W Scott McGraw, Roan McNab, Luciano Montag, Jo Myers Thompson, Jacob Nabe-Nielsen, Michiko Nakagawa, Sanjay Nepal, Marilyn Norconk, Vojtech Novotný, Sean O'Donnell, Muse Opiang, Paul Ouboter, Kenneth Parker, N Parthasarathy, Kátia Pisciotta, Dewi Prawiradilaga, Catherine Pringle, Subaraj Rajathurai, Ulrich Reichard, Gay Reinartz, Katherine Renton, Glen Reynolds, Vernon Reynolds, Erin Riley, Mark-Oliver Rödel, Jessica Rothman, Philip Round, Shoko Sakai, Tania Sanaiotti, Tommaso Savini, Gertrud Schaab, John Seidensticker, Alhaji Siaka, Miles R Silman, Thomas B Smith, Samuel Soares de Almeida, Navjot Sodhi, Craig Stanford, Kristine Stewart, Emma Stokes, Kathryn E Stoner, Raman Sukumar, Martin Surbeck, Mathias Tobler, Teja Tscharntke, Andrea Turkalo, Govindaswamy Umapathy, Merlijn van Weerd, Jorge Vega Rivera, Meena Venkataraman, Linda Venn, Carlos Verea, Carolina Volkmer de Castilho, Matthias Waltert, Benjamin Wang, David Watts, William Weber, Paige West, David Whitacre, Ken Whitney, David Wilkie, Stephen Williams, Debra D Wright, Patricia Wright, Lu Xiankai, Pralad Yonzon, and Franky Zamzani. Averting biodiversity collapse in tropical forest protected areas. *Nature*, 489(7415):290–294, 2012.

[54] Li Yiming and David S. Wilcove. Threats to vertebrate species in China and the United States. *BioScience*, 55(2):147–153, 2005.

[55] Paul J Ferraro, Toshihiro Uchida, and Jon M Conrad. Price premiums for eco-friendly commodities: are 'green' markets the best way to protect endangered ecosystems? *Environmental and Resource Economics*, 32(3):419–438, 2005.

[56] A Zabel and B Roe. Optimal design of pro-conservation incentives. *Ecological Economics*, 69:126–134, 2009.

[57] T Dietz, Elinor Ostrom, and Paul C Stern. The struggle to govern the commons. *Science*, 302(5652):1907–1912, 2003.

[58] Lydia Beaudrot, Jorge A Ahumada, Timothy O'Brien, Patricia Alvarez-Loayza, Kelly Boekee, Ahimsa Campos-Arceiz, David Eichberg, Santiago Espinosa, Eric Fegraus, Christine Fletcher, Krisna Gajapersad, Chris Hallam, Johanna Hurtado, Patrick A Jansen, Amit Kumar, Eileen Larney, Marcela Guimarães Moreira Lima, Colin Mahony, Emanuel H Martin, Alex McWilliam, Badru Mugerwa, Mireille

Ndoundou-Hockemba, Jean Claude Razafimahaimodison, Hugo Romero-Saltos, Francesco Rovero, Julia Salvador, Fernanda Santos, Douglas Sheil, Wilson R Spironello, Michael R Willig, Nurul L Winarni, Alex Zvoleff, and Sandy J Andelman. Standardized assessment of biodiversity trends in tropical forest protected areas: the end is not in sight. *PLoS Biology*, 14(1):e1002357, 2016.

[59] Paul D Meek, Guy A Ballard, Jess Sparkes, Mark Robinson, Brad Nesbitt, and Peter J S Fleming. Camera trap theft and vandalism: occurrence, cost, prevention and implications for wildlife research and management. *Remote Sensing in Ecology and Conservation*, 5:160–168, 2019.

[60] Paul Glover-Kapfer, Carolina A Soto-Navarro, and Oliver R Wearn. Camera-trapping version 3.0: current constraints and future priorities for development. *Remote Sensing in Ecology and Conservation*, 5(3):209–223, 2018.

[61] Andrew Tilker, Jesse F Abrams, An Nguyen, Lisa Hörig, Jan Axtner, Julie Louvrier, Benjamin M Rawson, Hoa Anh Quang Nguyen, Francois Guegan, Thanh Van Nguyen, Minh Le, Rahel Sollmann, and Andreas Wilting. Identifying conservation priorities in a defaunated tropical biodiversity hotspot. *Diversity and Distributions*, 10(1):100331–100315, 2020.

[62] K. Bohmann, A. Evans, M. T. P. Gilbert, G. R. Carvalho, S. Creer, M. Knapp, D. W. Yu, and M. de Bruyn. Environmental DNA for wildlife biology and biodiversity monitoring. *Trends in Ecology and Evolution*, 29(6):358–367, 2014.

[63] Kristine Bohmann, Ida B Schnell, and M Thomas P Gilbert. When bugs reveal biodiversity. *Molecular Ecology*, 22(4):909–911, 2013.

[64] Sébastien Calvignac-Spencer, Fabian H Leendertz, M Thomas P Gilbert, and Grit Schubert. An invertebrate stomach's view on vertebrate ecology. *BioEssays*, 35(11):1004–1013, 2013.

[65] Ida Bærholm Schnell, Rahel Sollmann, Sébastien Calvignac-Spencer, Mark E Siddall, Douglas W Yu, Andreas Wilting, and M Thomas P Gilbert. iDNA from terrestrial haematophagous leeches as a wildlife surveying and monitoring tool – prospects, pitfalls and avenues to be developed. *Frontiers in Zoology*, 12(1):302, 2015.

[66] Rosie Drinkwater, Ida Bærholm Schnell, Kristine Bohmann, Henry Bernard, Géraldine Veron, Elizabeth L Clare, M Thomas P Gilbert, and Stephen J Rossiter. Using metabarcoding to compare the suitability of two blood-feeding leech species for sampling mammalian diversity in North Borneo. *Molecular Ecology Resources*, 19(1):105–117, 2019.

[67] Jan F Gogarten, Ariane Düx, Benjamin Mubemba, Kamilla Pléh, Constanze Hoffmann, Alexander Mielke, Jonathan Müller Tiburtius, Andreas Sachse, Roman M Wittig, Sébastien Calvignac-Spencer, and Fabian H Leendertz. Tropical rainforest flies carrying pathogens form stable associations with social nonhuman primates. *Molecular Ecology*, 28(18):4242–4258, 2019.

[68] Arthur Kocher, Benoit de Thoisy, François Catzeflis, Sophie Valière, Anne-Laure Bañuls, and Jérôme Murienne. iDNA screening: disease vectors as vertebrate samplers. *Molecular Ecology*, 26(22):6478–6486, 2017.

[69] Ida Bærholm Schnell, Kristine Bohmann, Sebastian E Schultze, Stine R Richter, Dáithí C Murray, Mikkel-Holger S Sinding, David Bass, John E Cadle, Mason J Campbell, Rainer Dolch, David P Edwards, Thomas N E Gray, Teis Hansen, Anh Nguyen Quang Hoa, Christina Lehmkuhl Noer, Sigrid Heise-Pavlov, Adam F

Sander Pedersen, Juliot Carl Ramamonjisoa, Mark E Siddall, Andrew Tilker, Carl Traeholt, Nicholas Wilkinson, Paul Woodcock, Douglas W Yu, Mads Frost Bertelsen, Michael Bunce, and M Thomas P Gilbert. Debugging diversity - a pan-continental exploration of the potential of terrestrial blood-feeding leeches as a vertebrate monitoring tool. *Molecular Ecology Resources*, 18(6):1282–1298, 2018.

[70] M Tessler, S R Weiskopf, L Berniker Systematics and, and 2018. Bloodlines: mammals, leeches, and conservation in southern Asia. *Systematics and Biodiversity*, 16(5):488–496, 2018.

[71] Niccolo Alfano, Anisha Dayaram, Jan Axtner, Kyriakos Tsangaras, Marie-Louise Kampmann, Azlan Mohamed, Seth T. Wong, M. Thomas P. Gilbert, Andreas Wilting, and Alex D. Greenwood. Non-invasive surveys of mammalian viruses using environmental DNA. *bioRxiv*, page 2020.03.26.009993, 2020.

[72] Jesse F Abrams, Lisa Hörig, Robert Brozovic, Jan Axtner, Alex Crampton-Platt, Azlan Mohamed, Seth T Wong, Rahel Sollmann, Douglas W Yu, and Andreas Wilting. Shifting up a gear with iDNA: from mammal detection events to standardized surveys. *Journal of Applied Ecology*, 18(3):511–512, 2019.

[73] S. R. Weiskopf, K. P. McCarthy, M. Tessler, H. A. Rahman, J. L. McCarthy, R. Hersch, M. M. Faisal, and M. E. Siddall. Using terrestrial haematophagous leeches to enhance tropical biodiversity monitoring programmes in Bangladesh. *Journal of Applied Ecology*, 55(4):2071–2081, 2018.

[74] Conrad P D T Gillett, Andrew J Johnson, Iain Barr, and Jiri Hulcr. Metagenomic sequencing of dung beetle intestinal contents directly detects and identifies mammalian fauna. *bioRxiv*, page 074849, 2016.

[75] D L Wu and C C Luo. Effect of human activity on community structure of small mammals in Ailao Mountain. *Zoological Research*, 14(1):35–41, 1993.

[76] Z. J. Wang, C. Carpenter, and S. S. Young. Bird distribution and conservation in the Ailao Mountains, Yunnan, China. *Biological Conservation*, 92(1):45–57, 2000.

[77] Hongmei Li, Xin Zhang, Dingqi Rao, and Hongyu Zhang. Research on the reptiles diversity in the east of Xinping Ailaoshan Nature Reserve. *Hubei Agricultural Sciences*, 51(16):3557–3559, 2012.

[78] W S Luo, S Y Zhao, Z Q Luo, and Q Wang. Population and distribution of *Nomascus concolor* in Jingdong jurisdiction of Ailaoshan National Nature Reserve. *Sichuan Journal of Zoology*, 26(3):600–603, 2007.

[79] Hongmei Li, Huixian Zhu, Lin Wang, and Jiazhong Liu. Biological characteristics and protection of *Tylototriton shanjing* at Mount Ailao in Xinping. *Journal of Chongqing College of Education*, 23(6):16–18, 2010.

[80] Hongmei Li. The distribution and perniciousness of *Rhabdophis subminiatus* at Ailaoshan in Xinping County. *Hubei Agricultural Sciences*, 50(4):800–801, 2011.

[81] Guosong Li, Xianming Yang, Hongyu Zhang, and Wei Li. Population and distribution of western black crested gibbon (*Nomascus concolor*) at Ailao Mountain, Xinping, yunnan. *Zoological Research*, 32(6):675–683, 2011.

[82] Dejun Kong, Fei Wu, Pengfei Shan, Jianyun Gao, Dao Yan, Weixiong Luo, and Xiaojun Yang. Status and distribution changes of the endangered green peafowl (*Pavo muticus*) in china over the past three decades (1990s–2017). *Avian Research*, 9(1):427, 2018.

[83] X.L. He, K Luo, Z Y Lu, and L X Lin. Preliminary camera-trapping survey on wild mammals and birds in Ailaoshan National Nature Reserve, Yunnan Province, China. *Acta Theriologica Sinica*, 38(3):318–322, 2018.

[84] Andrew J Tyre, Brigitte Tenhumberg, Scott A Field, Darren Niejalke, Kirsten Parris, and Hugh P Possingham. Improving precision and reducing bias in biological surveys: estimating false-negative error rates. *Ecological Applications*, 13(6):1790–1801, 2003.

[85] Laurent Lellouch, Sandrine Pavoine, Frédéric Jiguet, Hervé Glotin, and Jérôme Sueur. Monitoring temporal change of bird communities with dissimilarity acoustic indices. *Methods in Ecology and Evolution*, 5(6):495–505, 2014.

[86] Y. Q. Ji, L. Ashton, S. M. Pedley, D. P. Edwards, Y. Tang, A. Nakamura, R. Kitching, P. M. Dolman, P. Woodcock, F. A. Edwards, T. H. Larsen, W. W. Hsu, S. Benedick, K. C. Hamer, D. S. Wilcove, C. Bruce, X. Y. Wang, T. Levi, M. Lott, B. C. Emerson, and D. W. Yu. Reliable, verifiable and efficient monitoring of biodiversity via metabarcoding. *Ecology Letters*, 16(10):1245–1257, 2013.

[87] K Y Zhang, Y P Zhang, Y H Liu, and Y R Li. Vertical distribution characteristics of rainfall in the Ailao mountain. *Scientia Geographica Sinica*, 14(2):144–150, 1994.

[88] Z Q Zhang. Status quo of the biodiversity of Ailaoshan Nature Reserve and countermeasures for protection and management. *Forest Inventory and Planning*, 32(3):68–70, 2007.

[89] Yves Escoufier. Le traitement des variables vectorielles. *Biometrics*, 29(4):751–760, 1973.

[90] Ronald W. Davies, L. R. Linton, and F. J. Wrona. Passive dispersal of four species of freshwater leeches (Hirudinoidea) by ducks. *Freshwater Invertebrate Biology*, 1(4):40–44, 1982.

[91] Christopher T. Rota, Robert J. Fletcher Jr, Robert M. Dorazio, and Matthew G. Betts. Occupancy estimation and the closure assumption. *Journal of Applied Ecology*, 46(6):1173–1181, 2009.

[92] M. Fahmy, K.M. Williams, M. Tessler, S.R. Weiskopf, E. Hekkala, and M.E. Siddall. Multilocus metabarcoding of terrestrial leech bloodmeal iDNA increases species richness uncovered in surveys of vertebrate host biodiversity. *Journal of Parasitology*, 106(6):843–853, 2020.

[93] Shanlin Liu, Xin Wang, Lin Xie, Meihua Tan, Zhenyu Li, Xu Su, Hao Zhang, Bernhard Misof, Karl M Kjer, Min Tang, Oliver Niehuis, Hui Jiang, and Xin Zhou. Mitochondrial capture enriches mito-DNA 100 fold, enabling PCR-free mitogenomics biodiversity analysis. *Molecular Ecology Resources*, 16(2):470–479, 2016.

[94] Cheri M Ackerman, Cameron Myhrvold, Sri Gowtham Thakku, Catherine A Freije, Hayden C Metsky, David K Yang, Simon H Ye, Chloe K Boehm, Tinna-Sólveig F Kosoko-Thoroddsen, Jared Kehe, Tien G Nguyen, Amber Carter, Anthony Kulesa, John R Barnes, Vivien G Dugan, Deborah T Hung, Paul C Blainey, and Pardis C Sabeti. Massively multiplexed nucleic acid detection with Cas13. *Nature*, 582(7811):277–282, 2020.

[95] Paul D. N. Hebert, Peter M. Hollingsworth, and Mehrdad Hajibabaei. From writing to reading the encyclopedia of life. *Philosophical Transactions of the Royal Society B: Biological Sciences*, 371(1702):20150321, 2016.

[96] Jan F Gogarten, Constanze Hoffmann, Mimi Arandjelovic, Andreas Sachse, Kevin Merkel, Paula Dieguez, Anthony Agbor, Samuel Angedakin, Gregory Brazzola, Sorrel Jones, Kevin E Langergraber, Kevin Lee, Sergio Marrocoli, Mizuki Murai, Volker Sommer, Hjalmar Kühl, Fabian H Leendertz, and Sébastien Calvignac-Spencer. Fly-derived DNA and camera traps are complementary tools for assessing mammalian biodiversity. *Environmental DNA*, 2(1):63–76, 2019.

[97] J. A. Royle and W. A. Link. Generalized site occupancy models allowing for false positive and false negative errors. *Ecology*, 87(4):835–841, 2006.

[98] D. A. Miller, J. D. Nichols, B. T. McClintock, E. H. C. Grant, L. L. Bailey, and L. A. Weir. Improving occupancy estimation when two types of observational error occur: non-detection and species misidentification. *Ecology*, 92(7):1422–1428, 2011.

[99] Jim E. Griffin, Eleni Matechou, Andrew S. Buxton, Dimitrios Bormpoudakis, and Richard A. Griffiths. Modelling environmental DNA data; Bayesian variable selection accounting for false positive and false negative errors. *Journal of the Royal Statistical Society: Series C (Applied Statistics)*, 69(2):377–392, 2020.

[100] Piero Visconti, Stuart H. M. Butchart, Thomas M. Brooks, Penny F. Langhammer, Daniel Marnewick, Sheila Vergara, Alberto Yanosky, and James E. M. Watson. Protected area targets post-2020. *Science*, 364:239–241, 2019.

[101] Vanessa M. Adams, Piero Visconti, Victoria Graham, and Hugh P. Possingham. Indicators keep progress honest: A call to track both the quantity and quality of protected areas. *One Earth*, 4(7):901–906, 2021.

[102] J. Andrew Royle and James D. Nichols. Estimating abundance from repeated presence–absence data or point counts. *Ecology*, 84(3):777–790, 2003.

[103] J. Andrew Royle. N-mixture models for estimating population size from spatially replicated counts. *Biometrics*, 60(1):108–115, 2004.

[104] Connor M. Wood, Viorel D. Popescu, Holger Klinck, John J. Keane, R.J. Gutiérrez, Sarah C. Sawyer, and M. Zachariah Peery. Detecting small changes in populations at landscape scales: a bioacoustic site-occupancy framework. *Ecological Indicators*, 98:492–507, 2019.

[105] Marie-Louise Kampmann, Ida Bærholm Schnell, Randi Holm Jensen, Jan Axtner, Adam F. Sander, Anders J. Hansen, Mads F. Bertelsen, Alex D. Greenwood, M. Thomas P. Gilbert, and Andreas Wilting. Leeches as a source of mammalian viral DNA and RNA - a study in medicinal leeches. *European Journal of Wildlife Research*, 63(2):36, 2017.

[106] Rory Gibb, David W. Redding, Kai Qing Chin, Christl A. Donnelly, Tim M. Blackburn, Tim Newbold, and Kate E. Jones. Zoonotic host diversity increases in human-dominated ecosystems. *Nature*, 584(7821):398–402, 2020.

[107] Yinqiu Ji. Ecec_ailaishan_leeches_bioinfo_pipeline, 2020.

[108] Antoine Guisan, Stuart B. Weiss, and Andrew D. Weiss. GLM versus CCA spatial modeling of plant species distribution. *Plant Ecology*, 143(1):107–122, 1999.

[109] Gentile Francesco Ficetola, Eric Coissac, Stéphanie Zundel, Tiayyba Riaz, Wasim Shehzad, Julien Bessière, Pierre Taberlet, and Francois Pompanon. An *in silico* approach for the evaluation of DNA barcodes. *BMC Genomics*, 11(1):434, 2010.

[110] Emiliano Mori, Luca Nerva, and Sandro Lovari. Reclassification of the serows and gorals: the end of a neverending story? *Mammal Review*, 49(3):256–262, 2019.

[111] T.D. Phan, S. Nijhawan, S Li, and L. Xiao. *Capricornis sumatraensis. The IUCN Red List of Threatened Species 2020*, page e.T162916735A162916910, 2020.

[112] James D. Nichols, Larissa L. Bailey, Allan F. O'Connell Jr., Neil W. Talancy, Evan H. Campbell Grant, Andrew T. Gilbert, Elizabeth M. Annand, Thomas P. Husband, and James E. Hines. Multi-scale occupancy estimation and modelling using multiple detection methods. *Journal of Applied Ecology*, 45(5):1321–1329, 2008.

[113] Benedikt R. Schmidt, Marc Kéry, Sylvain Ursenbacher, Oliver J. Hyman, and James P. Collins. Site occupancy models in the analysis of environmental DNA presence/absence surveys: a case study of an emerging amphibian pathogen. *Methods in Ecology and Evolution*, 4(7):646–653, 2013.

[114] Margaret E. Hunter, Sara J. Oyler-McCance, Robert M. Dorazio, Jennifer A. Fike, Brian J. Smith, Charles T. Hunter, Robert N. Reed, and Kristen M. Hart. Environmental DNA (eDNA) sampling improves occurrence and detection estimates of invasive Burmese pythons. *PLoS ONE*, 10(4):e0121655, 2015.

[115] Robert M. Dorazio and Richard A. Erickson. eDNAoccupancy: An R package for multiscale occupancy modelling of environmental DNA data. *Molecular Ecology Resources*, 18(2):368–380, 2018.

[116] Marc Kéry and J. Andrew Royle. *Applied Hierarchical Modeling in Ecology*, volume 1. Elsevier, London, UK, 2016.
