# Supplementary material for: Measuring protected-area effectiveness using vertebrate distributions from leech iDNA
Source: Nat Commun. 2022 Mar 23;13:1555. doi: 10.1038/s41467-022-28778-8 (PMC8943135; doi:10.1038/s41467-022-28778-8)
Supplement: Supplementary file 2 — Description of Additional Supplementary Files [file 41467_2022_28778_MOESM2_ESM.pdf]

Measuring Protected-Area Effectiveness using Vertebrate Distributions from Leech iDNA

Yinqiu Ji*, Christopher CM Baker*, Viorel D Popescu, Jiaxin Wang, Chunying Wu, Zhengyang Wang, Yuanheng Li, Lin Wang, Chaolang Hua, Zhongxing Yang, Chunyan Yang, Charles CY Xu, Alex Diana, Qingzhong Wen, Naomi E Pierce, and Douglas W Yu

*These authors contributed equally to this work.

Description of Additional Supplementary Files

File Name: Supplementary Data 1
Description: This file lists species detected in our study, including observed occupancy as well as their occupancy and detection estimates.

File Name: Supplementary Data 2
Description: This file provides a working list of species known to Kunming Institute of Zoology researchers from Ailaoshan, indicating whether each species was represented in our LSU or SSU reference sequence databases.

File Name: Supplementary Data 3
Description: This file compares working species lists for Ailaoshan from Kunming Institute of Zoology researchers against our matched and unmatched OTUs.

File Name: Supplementary Data 4
Description: This file contains representative sequences for each pre-OTU in the LSU dataset. Sequences are provided in FASTA format.

File Name: Supplementary Data 5
Description: This file contains representative sequences for each pre-OTU in the SSU dataset. Sequences are provided in FASTA format.

File Name: Supplementary Data 6
Description: This file provides tables of read counts in each OTU in each replicate sample, along with sample metadata. These tables represent the processed datasets immediately prior to occupancy modelling.
